# Toddler Screen Use Before Bed and Its Effect on Sleep and Attention: A Randomized Clinical Trial

**DOI:** 10.1001/jamapediatrics.2024.3997

**Published:** 2024-10-21

**Authors:** Hannah Pickard, Petrina Chu, Claire Essex, Emily J. Goddard, Katie Baulcombe, Ben Carter, Rachael Bedford, Tim J. Smith

**Affiliations:** 1 Centre for Brain and Cognitive Development, Birkbeck, University of London, London, United Kingdom; 2 Department of Biostatistics and Health Informatics, Institute of Psychology, Psychiatry and Neuroscience, King’s College London, London, United Kingdom; 3 King’s Clinical Trials Unit, Institute of Psychology, Psychiatry and Neuroscience, King’s College London, London, United Kingdom; 4 Department of Psychology, University of Bath, Bath, United Kingdom; 5 Centre for Brain and Behaviour, Department of Psychology, Queen Mary University of London, London, United Kingdom; 6 Creative Computing Institute, University of the Arts London, London, United Kingdom

## Abstract

**Importance:**

Toddler screen time has been associated with poorer sleep and differences in attention. Understanding the causal impact of screen time on early development is of the highest importance.

**Objective:**

To test (1) the feasibility of the 7-week parent-administered screen time intervention (PASTI) in toddlers (aged 16-30 months) who have screen time in the hour before bed and (2) the impact of PASTI on toddlers’ sleep and attention.

**Design, Setting, and Participants:**

This assessor-blinded, UK-based randomized clinical trial was conducted between July 2022 and July 2023. This was a single-site study that enrolled families with a toddler aged between 16 and 30 months, living within 75 miles of the Babylab, and with 10 minutes or more of screen time in the hour before bed on 3 or more days a week. Exclusion criteria were (1) a genetic or neurological condition, (2) premature birth (<37 weeks), and (3) current participation in another study.

**Interventions:**

Families were randomized (1:1:1) to (1) PASTI: caregivers removed toddler screen time in the hour before bed and used activities from a bedtime box instead (eg, reading, puzzles); (2) bedtime box (BB only): used matched before-bed activities, with no mention of screen time; or (3) no intervention (NI): continued as usual.

**Main Outcomes and Measures:**

Feasibility outcomes: participation rate, intervention adherence, retention, family experiences, and assessment acceptability. Efficacy outcomes: screen use, actigraphy-measured sleep, and eye-tracking attention measures.

**Results:**

A total of 427 families were screened, 164 were eligible (38.4%), and 105 families were randomized (mean [SD] age, 23.7 [4.6] months; 60 male [57%]). The trial was feasible, with 99% participant (104 of 105) retention and 94% of families (33 of 35) adhering to PASTI. PASTI showed reductions in parent-reported screen time (vs NI: Cohen *d* = −0.96; 95% CI, −1.32 to −0.60; vs BB only: Cohen *d* = −0.65; 95% CI, −1.03 to −0.27). PASTI showed small to medium improvements in objectively measured sleep efficiency (vs NI: Cohen *d* = 0.27; 95% CI, −0.11 to 0.66; vs BB only: Cohen *d* = 0.56; 95% CI, 0.17-0.96), night awakenings (vs NI: Cohen *d *= −0.28; 95% CI, −0.67 to 0.12; vs BB only: Cohen *d* = −0.31; 95% CI, −0.71 to 0.10), and reduced daytime sleep (vs NI: Cohen *d *= −0.30; 95% CI, −0.74 to 0.13) but no difference compared with BB only. There was no observable effect of PASTI on objective measures of attention. Compared with BB only, PASTI showed a difference on parent-reported effortful control (Cohen *d* = −0.40; 95% CI, −0.75 to −0.05) and inhibitory control (Cohen *d* = −0.48; 95% CI, −0.77 to −0.19), due to an increase in BB-only scores.

**Conclusions and Relevance:**

Results of this randomized clinical trial show that, supporting pediatric recommendations, removing screen time before toddler bedtime was feasible and showed modest preliminary beneficial effects on sleep. A future full confirmatory trial is needed before PASTI’s adoption by parents and pediatricians.

**Trial Registration:**

ISRCTN.org Identifier: ISRCTN58249751

## Key Points

**Questions:**

What is the feasibility and efficacy of conducting a randomized clinical trial of a parent-administered screen time intervention in the hour before bed on objectively measured toddler sleep and attention?

**Findings:**

In this randomized clinical trial including 105 families, the parent-administered screen time intervention proved highly feasible, and pilot efficacy findings suggest small to medium positive effects of screen time removal on objective sleep efficiency, night awakenings, and daytime nap duration but no effect on objective attention measures.

**Meaning:**

As currently recommended by pediatricians, parents were able to remove toddler screen time in the hour before bed, and this removal caused preliminary improvements in toddler sleep.

## Teaser

This randomized clinical trial investigates the effect of a parent-administered screen time intervention in the hour before bed on objectively measured toddler sleep and attention.

## Introduction

There has been a rapid increase in toddlers’ exposure to screens (eg, TV, tablets, smartphones),^1,2^ and screen use has been associated with poor sleep^3,4^ and differences in cognitive development (eg, attention).^5,6,7^ Current pediatric guidelines for toddlers recommend limiting screen time and avoiding it entirely in the hour before bed.^8,9^ However, the strength of evidence supporting this guideline in toddlers is weak.^9^ Given the potential impact on childhood health and cognitive function,^10^ there is a critical need for causal evidence on the impact of screen time in early development.

Sleep is crucial for brain maturation, and disruptions in sleep can have a significant impact on child development,^11^ leading to detrimental health outcomes.^12,13^ Negative associations between screen exposure and sleep problems, including sleep quantity and quality, in children are commonly reported.^3,4,14^ These associations are greatest when the screen exposure is before bed.^15^ Intervention studies in adults provide further support, with matched screen/nonscreen content interventions showing a direct causal impact of screens on sleep.^16^ A meta-analysis^17^ of screen time interventions in children demonstrated small improvements in sleep, although high-quality evidence is highly limited. One education-based, healthy behaviors intervention, which included reduced screen time in 2- to 5-year-olds, showed an increase in parent-reported sleep duration,^18^ whereas others have shown no effect.^19,20^ A similar healthy lifestyle program, including reduced screen time, showed no effect on actigraphy-measured sleep duration at 3-month follow-up, but there was a reduction in sleep at 6-month follow-up.^21^

Poor sleep is associated with children’s ability to focus their attention: insufficient sleep leads to reduced concentration,^22^ and sleep problems are common among children with attention problems.^23,24^ Exposure to screen content across childhood is associated with later attentional problems.^7,25,26,27^ Research using gaze-contingent experimental methods has demonstrated that 18-month-olds with high touch screen use show enhanced saliency-driven attention (eg, rapid orienting to the odd one out) and reduced voluntary, goal-directed attention,^5,6^ highlighting potentially important developmental differences in attention. However, the direction of effects cannot be interpreted without evidence from intervention studies modifying toddler screen time.

Previous parent/child education programs have found that behavior change interventions were effective in replacing screen time with other activities in school-aged children.^28,29,30^ For example, when educated on the benefits of removing screen time, parents of 4- to 6-year-olds reported decreased attention problems and increased sleep quality.^31^ To date and to our knowledge, no interventions have objectively measured the impact of removing screen time in the hour before bed on toddler sleep and attention or been able to disentangle the impact of screen use from the before-bed activities it may displace (eg, reading, calming play).

In this study, we evaluated the feasibility and pilot efficacy of a 7-week parent-administered screen time intervention (PASTI) in 16- to 30-month-old toddlers who have screen use in the hour before bed. PASTI was modeled on effective parent-education screen time interventions in older children,^32^ and cocreated with caregivers and early years practitioners. Parents in PASTI were instructed to avoid all screens in the hour before their child’s bedtime and were given a family bedtime box with alternative before-bed activities, including activity cards and age-appropriate toys. The effect of PASTI on toddler sleep and attention was objectively measured and compared with no intervention (NI) and bedtime box only (BB only; ie, active control group) in which parents were given similar before-bed activities to the PASTI group but were not instructed to remove screen time. Comparing PASTI with BB only allows the independent impact of screen time removal to be disentangled from the before-bed activities that replace it.

## Methods

### Trial Design

This study was a 3-arm blinded (assessor, investigators, and analyst) pilot and feasibility randomized clinical trial (RCT) in toddlers over a 7-week period. The study was conducted at the Birkbeck Babylab and in families’ homes. Ethical approval was from Birkbeck, University of London Research Ethics Committee (reference 2122037). The preregistered trial protocol^33^ and statistical analysis plan are in Supplement 1 and Supplement 2, respectively. This study followed the Consolidated Standards of Reporting Trials (CONSORT) reporting guidelines.

### Sample and Selection Criteria

The study enrolled families with a toddler aged between 16 and 30 months, living within 75 miles of the Babylab, and with 10 or more minutes of screen time in the hour before bed on 3 or more days a week. Exclusion criteria were as follows: (1) a genetic or neurological condition, (2) premature birth (<37 weeks), and (3) current participation in another study. Demographic data (eg, ethnicity; socioeconomic status [SES]), were collected via a parent-report prescreen questionnaire. Parent and child ethnicity were reported by the parent under the following categories: Asian or Asian British, Black or African or Caribbean or Black British, multiethnic, White, or other (including Arab). Ethnicity was gathered to identify the representativeness of our sample to the UK population.

A target sample size of 105 (35 per group) was found to be sufficient to estimate the key unknown parameters necessary to power a full confirmatory RCT.^34^ For example, we would be able to estimate a dropout rate of 20% to within a 95% CI of ±7.6.

### Randomization

After providing written informed consent, families were randomly assigned using the King’s Clinical Trials Unit (KCTU)^35^ web-based system to either PASTI, BB only, or NI (1:1:1). The sequence was generated using minimization^35^ by KCTU and used child sex, age at randomization (17-24.4 months vs 24.5-31 months), and SES (Index of Multiple Deprivation [IMD] quintiles, 1-5) as factors.

### Blinding

Families were blind to the purpose of the trial; they were initially told it was to investigate how bedtime activities impact toddler sleep and attention, with no mention of before-bed screen time (until either randomization to the PASTI arm or after-trial debrief). Assessors were blinded to allocation. The trial statistician (P.C.) was blinded until the trial steering committee approved the statistical analysis plan (Supplement 2), and the senior statistician (B.C.) was blinded until database lock. One researcher (H.P.) was unblind for arm allocation. All other researchers were blind until the database was locked and analyzed.

### Procedure

The trial procedure included a pretest and posttest measurement design, with baseline home assessments (2 weeks before randomization) and laboratory assessments (immediately before randomization), and follow-up home assessments (last 2 weeks of the intervention) and laboratory assessments (after the final day of the intervention) (eFigure 1 in Supplement 3). Baseline questionnaires included the Brief Infant Sleep Questionnaire–Revised (BISQ-R),^36^ Early Childhood Behavior Questionnaire (ECBQ),^37^ Vineland Adaptive Behavior Scales,^38^ State and Trait Anxiety Inventory^39^ and questions about daytime activity levels. Before-bed activities, including screen use, were measured using a biweekly bedtime activity diary on a weekday and weekend day. Same-day completion was encouraged, with a cutoff of 12 PM the next day (eAppendix 6 in Supplement 3 contains details of the steps taken to minimize reporter bias).

Toddler sleep was captured using a lightweight and unobtrusive actigraphy device (MotionWatch 8 [CamNtech]) previously used in children.^40,41^ The watch was worn on the ankle for 6 to 9 days before randomization. Actigraph activity is measured in counts defined as the peak acceleration recorded each second relative to a not-moving threshold. Each value per second is summed over the epoch and recorded as the epoch count. The Actigraph data were collected at 5- and 15-second epochs. Counts across the epochs were automatically summed to 30-second epochs for analysis. A parent-reported sleep diary was collected to aid the detection of daytime naps and apply exclusions (eg, watch removal, car/buggy movement, not typical day/night^42^).

During the baseline laboratory assessment, toddlers completed 3 gaze-contingent eye-tracking experiments using an EyeLink 1000 plus (SR Research Ltd) measuring visual attention: the visual search task (eFigure 2 in Supplement 3),^5,43^ antisaccade task (eFigure 3 in Supplement 3),^6^ and gap-overlap task (eFigure 4 in Supplement 3).^6^ Full task descriptions are available in eAppendix 1 in Supplement 3. In the visual search task, toddlers search for a target red apple among distractors. Saccadic reaction times (RTs) to fixate the target were recorded. In the antisaccade task, toddlers fixate a central stimulus and must ignore a peripheral salient distractor to locate the target animation on the opposite side of the screen. Saccadic RTs to fixate the distractor (prosaccade latency) and frequency of saccades toward the target (antisaccade proportion) were recorded. In the gap-overlap task, toddlers shift their attention from a central stimulus (CS) to a peripheral target (PT) under 3 conditions (baseline: PT appears as CS disappears; gap: 200 milliseconds between CS offset and PT onset; overlap: CS remains present after PT onset). Saccadic RTs to fixate the PT were recorded. The Mullen Scales of Early Learning^44^ was administered to measure global development. All baseline assessments were repeated at follow-up.

### Interventions

After the baseline laboratory assessment, families were randomized into 1 of 3 intervention arms and given instructions describing the 7-week trial (eAppendix 2 in Supplement 3). The intervention and materials were cocreated with parents and early-years practitioners through a series of workshops and focus groups (eAppendix 3 and 4 in Supplement 3).

#### PASTI

Families randomized to the PASTI group were asked to remove screen time from their child in the hour before bed. Families received a family bedtime box with tips on alternative before-bed activities, including activity cards and age-appropriate toys (eAppendix 3 and eFigure 5 in Supplement 3), to use with their child in the hour before bed. In week 1, families had a video/phone call with an unblinded researcher to reflect on their strategies for removing screen time. Throughout the trial, caregivers completed a daily Screen Time Questionnaire and biweekly bedtime activity diary that captured before-bed activities (including screen use) (eAppendix 5 in Supplement 3).

#### BB Only and NI

In the BB-only group, families received identical materials to PASTI (ie, family bedtime box) but without any mention of removing screen time. Families completed the biweekly bedtime activity diary. In the NI group, families received no materials and were asked to continue with their toddler’s before-bed activities as usual. Families completed the biweekly bedtime activity diary.

### Outcomes

#### Feasibility Outcomes

Feasibility outcomes include participation rate, intervention adherence, retention to the follow-up laboratory assessment, family experiences, and assessment acceptability. Intervention adherence was defined as the mean proportion of days with no parent-reported screen time in the hour before bed, calculated from the Screen Time Questionnaire and bedtime activity diary throughout the intervention (weeks 1-6). The acceptability of PASTI and assessment measures were determined through a debrief questionnaire. Feasibility was assessed using a traffic light system (depicted under Intervention Feasibility in the Results section).

#### Efficacy Outcomes

Screen use duration in the hour before bed was measured using the mean of a weekday and weekend bedtime activity diary. Sleep outcomes were captured using actigraphy and parent-reported questionnaires. Actigraph data were scored automatically for sleep/wake using the MotionWare software, version 1.1.20 (CamNtech), between the markers *lights out* and *got up*, which were set manually by blinded researchers using a data-driven approach to locate a drop in motion. A low-sensitivity threshold was used, ie, an activity score greater than 80 counts per epoch was scored as wake.^45,46,47^ Actigraphy-measured sleep metrics included the following: (1) total night-time sleep duration, using Actigraph wake/sleep categorization; (2) sleep efficiency, defined as total nighttime sleep as a percentage of time in bed; (3) total daytime sleep duration, using Actigraph wake/sleep categorization; and (4) number of night awakenings, defined as a period of 5 or more consecutive minutes with activity counts classified as wake, calculated using the epoch by epoch sleep/wake categorization. Night awakenings were collapsed if they appeared within 10 minutes of each other. Sleep onset latency was captured using the parent-reported BISQ. Eye-tracking attention outcomes included the following: (1) single search saccadic RT from the visual search task, (2) prosaccade saccadic RT and proportion of antisaccades in the antisaccade task, and (3) baseline saccadic RT and disengagement saccadic RT (baseline RT − overlap RT) from the gap-overlap task. Parent-reported effortful control and the subscale inhibitory control were captured using the ECBQ.

### Statistical Analysis

Data analysis was performed using Stata, version 18 (StataCorp). The feasibility analysis included all randomized families, and pilot efficacy analyses used complete cases. Demographic and efficacy outcomes were summarized by group using descriptive statistics. Rates/proportions and corresponding 95% CIs were reported for the feasibility parameters and assessed against the predefined success metrics. Adjusted mean differences (MDs) for efficacy outcomes were obtained using linear regression for each continuous outcome predicted by allocation arm, baseline values of outcomes, and minimization factors (child sex, age, and IMD). Cohen *d* is reported as a measure of effect size.

## Results

**Figure 1.**
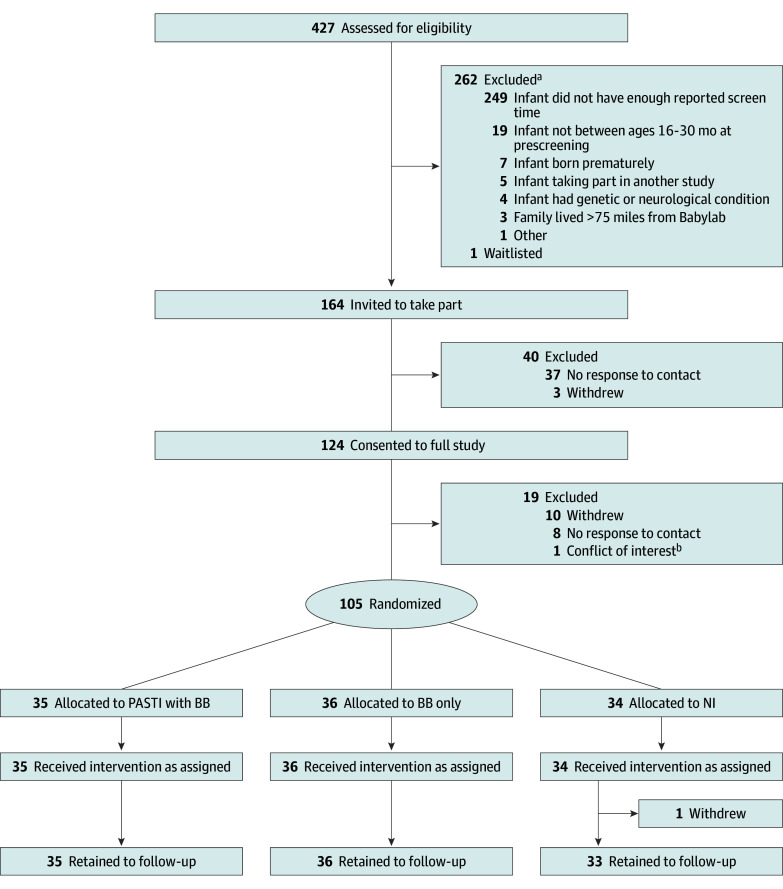
Consolidated Standards of Reporting Trials (CONSORT) Diagram BB indicates bedtime box; NI, no intervention; PASTI, parent-administered screen time intervention. ^a^Families may have been excluded for more than one reason. ^b^One family excluded at baseline laboratory visit due to conflict of interest.

Of the 427 families screened for eligibility, 164 were eligible (38.4%), and 105 families (mean [SD] age, 23.7 [4.6] months; 45 female [43%]; 60 male [57%]) were randomized to either PASTI (35 [33%]), BB only (36 [34%]), or NI (34 [32%]) (Figure 1). Our sample was socioeconomically and ethnically diverse: 47% of families (50 of 105) were from the 2 most disadvantaged IMD quintiles and 40% of toddlers (41 of 102; 3 missing) were from a non-White ethnic background. Specifically, child ethnicity was identified as 10 Asian or Asian British (10%), 4 Black or African or Caribbean or Black British (4%), 18 multiethnic (17%), 61 White (58%), 9 other (9%), and 3 missing (3%). Parent/caregiver ethnicity was identified as 16 Asian or Asian British (15%); 5 Black or African or Caribbean or Black British (5%); 6 multiethnic (6%); 67 White (64%); and 11 other (10%) (Table 1). The median (IQR) screen time before bed was 13 (4-23) minutes in the total sample (eTables 1 and 2 in Supplement 3 contain other before-bed activities). No adverse effects from the trial were reported.

**Table 1.** Study Sample Baseline Child and Parent Demographics

Minimization factors and baseline demographics	No. (%)			
	PASTI (n = 35)	BB only (n = 36)	NI (n = 34)	Overall (n = 105)
Child sex				
Female	15 (43)	15 (42)	15 (44)	45 (43)
Male	20 (57)	21 (58)	19 (56)	60 (57)
IMD Quintile				
1	5 (14)	4 (11)	5 (15)	14 (13)
2	12 (34)	13 (36)	11 (32)	36 (34)
3	8 (23)	8 (22)	8 (24)	24 (23)
4	5 (14)	6 (17)	6 (18)	17 (16)
5	5 (14)	5 (14)	4 (12)	14 (13)
Child age group at randomization, mo				
17-24.4	18 (51)	18 (50)	17 (50)	53 (50)
24.5-31	17 (49)	18 (50)	17 (50)	52 (50)
Child ethnicity				
Asian or Asian British	4 (11)	2 (6)	4 (12)	10 (10)
Black or African or Caribbean or Black British	2 (6)	1 (3)	1 (3)	4 (4)
Multiethnic	4 (11)	9 (25)	5 (15)	18 (17)
White	22 (63)	18 (50)	21 (62)	61 (58)
Other	3 (9)	5 (14)	1 (3)	9 (9)
Missing	0	1 (3)	2 (6)	3 (3)
Does your child have any medical conditions?				
No	32 (91)	34 (94)	32 (94)	98 (93)
Yes	3 (9)	2 (6)	2 (6)	7 (7)
Any siblings				
No siblings	18 (51)	23 (64)	23 (68)	64 (61)
≥1 Sibling	17 (49)	13 (36)	11 (32)	41 (39)
Younger siblings				
0	34 (97)	34 (94)	31 (91)	99 (94)
1	0	2 (6)	3 (9)	5 (5)
2	1 (3)	0	0	1 (1)
Older siblings				
0	19 (54)	25 (69)	25 (74)	69 (66)
1	13 (37)	7 (19)	8 (24)	28 (27)
≥2	3 (9)	4 (11)	1 (3)	8 (8)
Caregiver age, mean (SD), y	35 (5)	36 (5)	36 (4)	36 (5)
Who is filling out this questionnaire?				
Mother	35 (100)	34 (94)	34 (100)	103 (98)
Father	0	2 (6)	0	2 (2)
Respondent is sole caregiver				
No	29 (83)	30 (83)	30 (88)	89 (85)
Yes	6 (17)	6 (17)	4 (12)	16 (15)
Caregiver ethnicity				
Asian or Asian British	5 (14)	7 (19)	4 (12)	16 (15)
Black or African or Caribbean or Black British	2 (6)	2 (6)	1 (3)	5 (5)
Multiethnic	1 (3)	2 (6)	3 (9)	6 (6)
White	24 (69)	19 (53)	24 (71)	67 (64)
Other	3 (9)	6 (17)	2 (6)	11 (10)
Caregiver highest education				
School leaving qualification or equivalent	2 (6)	4 (11)	1 (3)	7 (7)
College or equivalent	3 (9)	5 (14)	3 (9)	11 (10)
University or equivalent	14 (40)	11 (31)	16 (47)	41 (39)
Post-graduate or equivalent	16 (46)	15 (42)	14 (41)	45 (43)
NA	0	1 (3)	0	1 (1)
Caregiver speaks fluent English?				
No	0	0	1 (3)	1 (1)
Yes	35 (100)	36 (100)	33 (97)	104 (99)
Do you live in greater/central London?				
No	6 (17)	0	7 (21)	13 (12)
Yes	29 (83)	36 (100)	27 (79)	92 (88)
Completed weeks of pregnancy, mean (SD)	39.4 (1.3)	39.5 (1.2)	39.7 (1.3)	39.5 (1.2)

Abbreviations: BB, bedtime box; IMD, Index of Multiple Deprivation; NI, no intervention.

### Intervention Feasibility

Our trial met all metrics for success, indicating that the trial was feasible (Table 2). Overall, 99% of families (104 of 105) were retained to follow-up. Adherence to PASTI was high, with 94% of families (33 of 35) reporting no screen time in the hour before bed on 60% or more of daily screen time questionnaires (mean proportion of nights without screen time during intervention period was 89%; 95% CI, 84%-94%). Furthermore, 94% of families (33 of 35) completed the PASTI debrief questionnaire, and of those, 97% (32 of 33) felt supported during the trial and 85% found the intervention easy to administer, with the majority of PASTI families (57% [19 of 33]) using the family bedtime box activities most days of the week (compared to 79% [26 of 33] in BB-only group).

**Table 2.** Traffic Light System to Assess Parent-Administered Screen Time Intervention (PASTI) Feasibility

Metric	Result (95% CI)	Red/amber/green (%)
Randomization (No. of participants randomized overall)	105 randomized	Green (≥105)
PASTI daily questionnaire completion (% of participants randomized to PASTI and retained to laboratory follow-up that complete ≥60% of daily screen time questionnaires)	31/35 is 89% (73%-97%)^a^	Green (≥80)
PASTI adherence to screen time removal (week 1 to week 6) (% of participants randomized to PASTI that report no screen time on ≥60% of daily screen time questionnaires completed)	33/35 is 94% (81%-99%)^a^	Green (≥70)
PASTI debrief questionnaire completion (% of participants randomized to PASTI that compete the debrief questionnaire)	33/35 is 94% (81%-99%)^a^	Green (≥75)
Retention (% of randomized participants attending follow-up laboratory visit)	104/105 is 99% (95%-99%)^a^	Green (≥75)

^a^ Red/amber/green metric of success is based on the point estimate. Performance metrics in the green zone indicate that a full trial is feasible. Amber indicates that the trial may be feasible but modifications/monitoring is required. The red zone indicates that the current trial may not be feasible.

### Intervention Efficacy and Screen Time

Table 3 shows descriptive statistics for baseline and follow-up outcomes and adjusted differences between PASTI and other groups for follow-up efficacy outcomes.

**Table 3.** Descriptive Statistics for Baseline and Follow-Up Outcomes and Adjusted Mean Difference Effect Estimates for Follow-Up Outcomes After Controlling for Minimization Factors (Child Sex, Child Age, and Index of Multiple Deprivation) and Baseline Efficacy Outcome

Efficacy outcomes	Mean (SD) [No.]						Adjusted estimates, mean difference (95% CI) [No.]	
	Baseline			Follow-up			PASTI vs BB-only	PASTI vs NI
	PASTI (n = 35)	BB only (n = 36)	NI (n = 34)	PASTI (n = 35)	BB-only (n = 36)	NI (n = 34)		
Screen use								
Mean screen use in hour before bed	15 (15) [29]	18 (14) [28]	14 (13) [30]	1 (3) [29]	10 (11) [27]	13 (10) [31]	−9.00 (−14.28 to −3.71) [75]	−13.33 (−18.34 to −8.31) [75]
Sleep								
Mean total night-time sleep duration	606 (49) [31]	606 (54) [32]	601 (39) [32]	596 (55) [27]	595 (51) [27]	590 (37) [27]	−0.96 (−20.45 to 18.53) [77]	2.38 (−16.57 to 21.33) [77]
Mean total day-time sleep duration	86 (49) [29]	74 (50) [24]	75 (34) [21]	82 (54) [23]	75 (50) [20]	84 (33) [22]	−2.30 (−22 to 17.39) [57]	−13.77 (−33.54 to 5.99) [57]
Mean frequency of night awakenings	1 (1) [31]	1 (0) [32]	1 (1) [32]	1 (0) [27]	1 (1) [27]	1 (0) [27]	−0.23 (−0.53 to 0.07) [77]	−0.21 (−0.50 to 0.09) [77]
Mean sleep efficiency	88 (3) [31]	89 (2) [32]	88 (2) [32]	88 (2) [27]	87 (2) [27]	87 (2) [27]	1.40 (0.42 to 2.38) [77]	0.68 (−0.27 to 1.63) [77]
BISQ-R sleep onset latency	35 (27) [35]	41 (25) [36]	29 (16) [34]	29 (29) [34]	31 (20) [36]	27 (17) [32]	0.99 (−9.03 to 11.01) [102]	0.09 (−10.24 to 10.41) [102]
Attention								
VST single search saccadic reaction time	1041 (316) [34]	1065 (384) [36]	1029 (327) [34]	1082 (491) [33]	1051 (395) [35]	1040 (401) [32]	52.20 (−152.38 to 256.77) [100]	59.29 (−150.39 to 268.96) [100]
AT prosaccade saccadic reaction time (preswitch)	317 (32) [31]	310 (35) [34]	318 (29) [33]	304 (33) [31]	303 (44) [31]	305 (38) [26]	−2.05 (−21.99 to 17.89) [85]	−0.43 (−21.1 to 20.25) [85]
AT proportion of antisaccades (preswitch)	25 (29) [33]	14 (19) [35]	22 (20) [33]	25 (25) [31]	19 (19) [31]	27 (30) [28]	4.84 (−7.58 to 17.25) [90]	−1.24 (−13.78 to 11.3) [90]
GT baseline saccadic reaction time	353 (76) [30]	338 (57) [31]	342 (61) [32]	348 (82) [31]	339 (101) [27]	339 (51) [25]	11.83 (−18.42 to 42.08) [78]	−7.95 (−38.4 to 22.5) [78]
GT disengagement saccadic reaction time	132 (96) [30]	142 (98) [31]	153 (87) [32]	116 (93) [31]	100 (118) [27]	107 (83) [25]	20.69 (−22.67 to 64.04) [78]	21.88 (−21.53 to 65.29) [78]
ECBQ–short form effortful control	3.8 (1.2) [35]	3.7 (1.2) [36]	3.8 (1.1) [34]	3.7 (1.2) [34]	4.2 (1.1) [36]	3.9 (0.7) [32]	−0.21 (−0.39 to −0.03) [102]	0.08 (−0.11 to 0.27) [102]
ECBQ–short form inhibitory control	4.7 (0.6) [35]	4.6 (0.5) [36]	4.6 (0.5) [34]	4.8 (0.7) [34]	4.9 (0.6) [36]	4.6 (0.4) [32]	−0.55 (−0.88 to −0.22) [102]	−0.17 (−0.51 to 0.17) [102]

Abbreviations: AT, antisaccade task; BB, bedtime box; BISQ-R, Brief Infant Sleep Questionnaire–Revised; ECBQ, Early Childhood Behavior Questionnaire; GT, gap-overlap task; NI, no intervention; PASTI, parent-administered screen time intervention; VST, visual search task.

**Figure 2.**
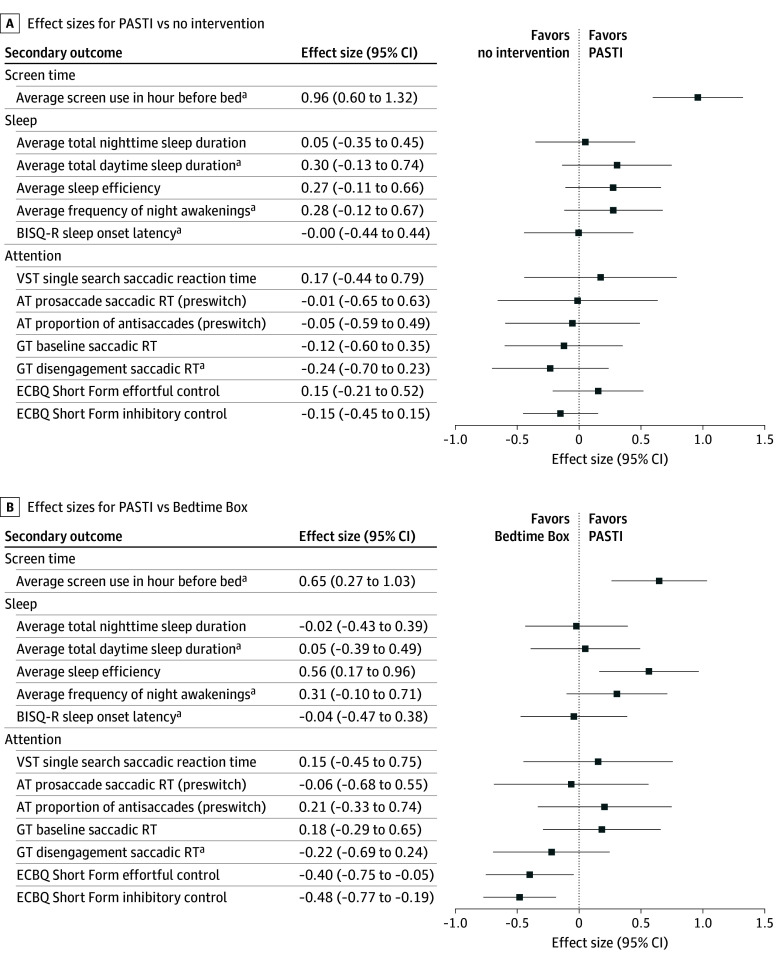
Forest Plots of Effect Sizes for 2 Comparisons A, Parent-administered screen time intervention (PASTI) vs no intervention (NI) comparison. B, PASTI vs bedtime box–only comparison. AT indicates antisaccade task; BISQ-R, Brief Infant Sleep Questionnaire–Revised; ECBQ, Early Childhood Behavior Questionnaire; GT, gap-overlap task; RT, reaction time; VST, visual search task. ^a^Denotes outcome measures for which the effect size is reversed in the plot as a lower value was better. The original direction of these reversed effect sizes is reported in the text.

There was a large effect of the PASTI intervention on parent-reported screen use in the hour before bed, with less screen time in PASTI compared with NI (adjusted MD = −13.33; Cohen *d* = −0.96; 95% CI, −1.32 to −0.60) and BB only (adjusted MD = −9.00; Cohen *d* = −0.65; 95% CI, −1.03 to −0.27) (Table 3 and Figure 2).

### Sleep

At follow-up, PASTI participants had shorter mean daytime sleep duration (adjusted MD = −13.77; Cohen *d *= −0.30; 95% CI, −0.74 to 0.13), fewer night awakenings (adjusted MD = −0.21; Cohen *d *= −0.28; 95% CI, −0.67 to 0.12), and higher sleep efficiency (adjusted MD = 0.68; Cohen *d* = 0.27; 95% CI, −0.11 to 0.66) compared with the NI group, with small to moderate effects and CIs crossing zero. Compared with the BB-only group, PASTI families had fewer night awakenings (adjusted MD = −0.23; Cohen *d* = −0.31; 95% CI, −0.71 to 0.10) with a clearer difference emerging for increased sleep efficiency (adjusted MD = 1.40; Cohen *d* = 0.56; 95% CI, 0.17-0.96).

### Attention

There was no clear difference between PASTI and NI for objective or parent-report attention measures (Figure 2 and Table 3). Compared with BB only, PASTI showed no difference on objective attention measures but a clear difference on parent-reported effortful control (adjusted MD = −0.21; Cohen *d* = −0.40; 95% CI, −0.75 to −0.05) and inhibitory control (adjusted MD = −0.55; Cohen *d* = −0.48; 95% CI, −0.77 to −0.19), due to an increase in BB-only scores. Comparisons between BB-only vs NI groups are available in eFigure 6 in Supplement 3.

## Discussion

To our knowledge, the current study presents the first RCT of before-bed toddler screen time on objectively measured sleep and attention. The trial demonstrated excellent feasibility, with 99% of families retained throughout the intervention period and 94% reporting adherence to screen removal. There was a reduction in parent-reported before-bed screen time between PASTI and other arms, confirming the feasibility of parent-education interventions previously used in older children.^32^

Pilot efficacy findings suggested an improvement in sleep efficiency in the PASTI arm compared with BB only and, to a lesser extent, compared with NI. Poor sleep efficiency is commonly observed among individuals with sleep problems,^48,49,50^ and therefore, this novel finding has important implications for supporting toddlers’ sleep quality. There was also a preliminary indication of fewer night awakenings for PASTI, although CIs crossed zero. The mechanism(s) by which before-bed screen time may negatively impact toddler sleep are not fully understood, but our preliminary results suggest that it may be due to the screen use itself, rather than displaced activities, as BB only encouraged the same before-bed activities as PASTI (eTable 2 in Supplement 3).

Alongside improved sleep quality, we hypothesized increased nighttime sleep duration and decreased daytime sleep for the PASTI arm, indicative of a more mature pattern of sleep.^51,52^ No clear differences for nighttime sleep were observed. Previous meta-analyses^17^ suggest that the impact of screen time removal on sleep duration is often small. Our reduction of before-bed screen time (9-13.3 minutes per day; 15%-22.2% of the before-bed hour) may be insufficient to change nighttime sleep duration. At this age, parents generally dictate when their toddler is put down to sleep, potentially limiting the impact of PASTI on nighttime sleep duration and making objectively measured sleep efficiency a better measure of intervention efficacy. A small effect, with CIs spanning zero, was seen for reduced daytime sleep in PASTI vs NI, although there was a reduced sample with nap data (n = 57). Further research should consider the broader effects of PASTI, including sleep regularity,^53^ given its importance for later health outcomes and cognitive function.^54,55,56^

There was no clear difference between PASTI and NI for objective or parent-reported attention measures. Previous longitudinal studies have demonstrated associations between high screen use and enhanced saliency driven attention/reduced goal-directed attention.^5,6^ In the current study, removing before-bed screen time did not change toddler attention. The large CIs observed for the attention efficacy metrics (eg, see CIs for visual search task single search saccadic reaction time in Figure 2) suggest that our sample size and sampling strategy may have been inadequate, signifying the need for a full confirmatory PASTI trial or for targeted sampling, eg, individuals scoring low on prescreen attention control.

In comparison with BB only, the PASTI arm showed no difference in objective attention measures but differed on parent-reported effortful control and inhibitory control. This unexpected finding was driven by an increase in BB-only scores; it may be due to greater use of the box activities (explained under Intervention Feasibility in the Results section) promoting better effortful/inhibitory control abilities, or to an increased opportunity for caregivers to observe their child’s effortful/inhibitory control skills during nightly dyadic play. This requires further investigation and objective replication in a confirmatory trial.

### Strengths and Limitations

Our findings support current pediatric guidance to avoid screen time in the hour before toddler bedtime, and our study has several strengths. PASTI is a low-cost, easy-to-implement intervention that is inclusive of diverse family profiles. These pilot efficacy findings require replication in a fully powered confirmatory trial. Future noninferiority trials are needed to determine which aspects of the intervention (week 1 call, text message reminders, bedtime box) are critical. Despite clear measurement strengths, the current study also has some limitations. One limitation is parent-reported screen use, which may be subject to reporter-bias; the field critically requires methods for unobtrusively capturing toddlers’ multiscreen exposure. Future studies must also engage with the rich variety of toddler screen use (eg, types of content, context of use) and differing neurodevelopmental profiles that may moderate the impact of removing before-bed screen time.

## Conclusions

Results of this RCT reveal that removing screen time before toddler bedtime was feasible and showed modest preliminary beneficial effects on sleep. A future full confirmatory trial is needed before PASTI’s adoption by parents and pediatricians.

## Notes

Supplement 1.
Trial Protocol.

Supplement 2.
Statistical Analysis Plan.

Supplement 3. **eFigure 1.** Participant Timeline **eAppendix 1.** Eye-Tracking Experiment Details **eFigure 2.** Stimulus Sequence for Experimental Trials in the Visual Search Task **eFigure 3.** Stimulus Sequence for Experimental Trials in the Antisaccade Task **eFigure 4.** Stimulus Sequence for Experimental Trials in the Gap-Overlap Task **eAppendix 2.** Intervention Delivery **eAppendix 3.** Family Bedtime Box **eFigure 5.** Family Bedtime Box Materials **eAppendix 4.** Parent-Administered Screen Time Intervention (PASTI) Development **eAppendix 5.** Screen Use Questions Used in the Screen Use Questionnaire and Bedtime Activity Diary **eAppendix 6.** Parent-Reported Screen Time Measure **eTable 1.** Descriptives for Baseline and Follow-Up Secondary Outcomes **eTable 2.** Descriptives for Alternative Activities in the Hour Before Bed From the Bedtime Activity Diary **eFigure 6.** Forest Plot of Effect Sizes for Bedtime Box vs No Intervention Comparison
eReferences

Supplement 4.
Data Sharing Statement.
