# Supplementary material for: Toddler Screen Use Before Bed and Its Effect on Sleep and Attention: A Randomized Clinical Trial
Source: JAMA Pediatr. 2024 Oct 21;178(12):1270–9. doi: 10.1001/jamapediatrics.2024.3997 (PMC11581737; doi:10.1001/jamapediatrics.2024.3997)
Supplement: Supplement 1. — Trial Protocol. [file jamapediatr-e243997-s001.pdf]

Parent-Administered Screen Time Intervention (PASTI): A 7-week three arm assessor blinded Feasibility and Pilot Randomised Controlled Trial, compared to Bedtime Box Intervention and No Intervention (1:1:1) in toddlers.

Hannah Pickard[1] PhD., Petrina Chu[2,3] MSc., Claire Essex[1] MSc., Emily Goddard[1] BSc., Katie Baulcombe[4]., Ben Carter[2,3] PhD., Rachael Bedford[4,5*] PhD., Tim J. Smith[1,6*] PhD.

[1] Centre for Brain and Cognitive Development, Birkbeck, University of London

[2] Department of Biostatistics and Health Informatics, Institute of Psychology, psychiatry and neuroscience, King's College London

[3] King's Clinical Trials Unit, Institute of Psychology, psychiatry and neuroscience, King's College London

[4] Department of Psychology, University of Bath

[5] Department of Psychology, Queen Mary University London

[6] Creative Computing Institute, University of the Arts London

[*]Joint senior authors

Table of contents

Abstract.......... 5
Administrative information.......... 6
Introduction.......... 8
Background and rationale {6a}.......... 8
Intervention description {11a}.......... 10
Explanation for choice of comparators {6b}.......... 11
Objectives {7}.......... 13
Trial design {8}.......... 14
Methods: Participants.......... 14
Study setting {9}.......... 14
Eligibility criteria {10}.......... 14
Recruitment {15}.......... 15
Methods: Interventions.......... 16
Criteria for discontinuing or modifying allocated interventions {11b}.......... 16
Strategies to improve adherence to interventions {11c}.......... 17
Relevant concomitant care permitted or prohibited during the trial {11d}.......... 17
Methods: Outcomes.......... 18
Primary feasibility outcomes:.......... 18
Metrics of feasibility study acceptability.......... 19
Secondary outcomes:.......... 20
Participant timeline {13}.......... 21
Sample size {14}.......... 23
Methods: Allocation.......... 23
Sequence generation {16a}.......... 23
Concealment mechanism {16b}.......... 23
Implementation {16c}.......... 24
Methods: Blinding.......... 25
Who will be blinded {17a}.......... 25
Procedure for unblinding if needed {17b}.......... 26
Methods: Data collection, management, and analysis.......... 27
Plans for assessment and collection of outcomes {18a}.......... 27
Pre-screen Online Questionnaire.......... 27
Baseline and follow up home assessments.......... 27
Baseline and follow up lab assessments.......... 29
Intervention based assessments.......... 33

Qualitative Debrief Questionnaire and Interview........ 34
Plans to promote participant retention and complete follow-up {18b}........ 34
Data management {19}........ 35
Data collection and processing........ 35
Data checks and double entry into a separate research database........ 37
Data storage and extraction........ 38
Qualitative data analysis........ 38
Statistical methods for primary and secondary outcomes {20a}........ 39
Descriptive statistics of study population........ 39
Secondary clinical feasibility outcomes........ 41
Populations under investigation........ 42
Handling of withdrawals, losses to follow-up, and missing data........ 42
Methods: Monitoring........ 43
Composition of the coordinating centre and trial steering committee {5d}........ 43
Composition of the data monitoring committee, its role and reporting structure {21a}........ 44
Interim analyses {21b}........ 45
Adverse event reporting and harms {22}........ 45
Frequency and plans for auditing trial conduct {23}........ 48
Ethics and dissemination........ 48
Research ethics approval {24}........ 48
Protocol amendments {25}........ 48
Consent or assent {26a}........ 49
Additional consent provisions for collection and use of participant data and biological specimen........ 49
Confidentiality........ 49
Declaration of interest........ 50
Access to data........ 50
Ancillary and post-trial care........ 50
Dissemination plans........ 50
Author’s contributions........ 51
Plans to give access to the full protocol, participant level-data and statistical code........ 51
References........ 52

Old version	New version	Date	Summary of changes
1	2	22 July 2022	• Updating child eligibility age range to maximum of 30 months at pre-screening, update language throughout to indicate that child is aged 17 to 31 months at randomisation. Child age group split at randomisation is now 17-24.4 months vs 24.5-31 months<br>• Minor updates to details of eye-tracking lab assessments<br>• Inclusion of Bedtime Box arm in online debrief survey<br>• Increase in participant compensation amounts<br>• Clarification of two-stage participant consenting process<br>• Adding questions to capture child use of sleep medications
2	3	29 Mar 2023	• Clarifying age at randomisation<br>• Added a secondary sleep outcome: Sleep efficiency, as measured using Actigraphy<br>• Change in Research Assistant staff member<br>• Adding debrief materials to the appendix (Appendix H)<br><br>At this time, data collection is ongoing and we have not looked at any data.

Abstract

Background: Childhood screen time is on the rise and has been linked to changes in several developmental factors including toddler sleep and attention, two critical determinants of childhood health and cognitive function. Understanding the potential causal influence of screen time on toddler sleep and cognitive development (e.g., attention) is thus of the highest importance. Here, we propose the first pilot trial to assess the feasibility of implementing a Parent-Administered Screen Time Intervention (PASTI) in toddlers who have parent-reported screen time in the hour before bed.

Methods: This is a blinded feasibility and pilot randomised control trial with three parallel intervention groups. PASTI will be compared to a bedtime box intervention and no intervention in toddlers. Feasibility outcomes including participation, intervention adherence and follow up rates will be assessed, as well as the acceptability of PASTI using quantitative and qualitative methods.

Discussion: This study will provide preliminary data to inform the design of a larger randomised control trial to investigate of the impact of PASTI on toddlers sleep and attention.

Trial registrations: 29/04/2022 (https://www.isrctn.com/ISRCTN58249751)

Keywords

Screen time, Sleep, Attention, Toddlers, Intervention, Feasibility

Administrative information

Note: the numbers in curly brackets in this protocol refer to SPIRIT checklist item numbers[1].

**Title {1}**	Parent-Administered Screen Time Intervention (PASTI): A 7-week three arm assessor blinded Feasibility and Pilot Randomised Controlled Trial, compared to Bedtime Box Intervention and No Intervention (1:1:1) in toddlers.
**Trial registration {2a and 2b}**	ISRCTN registry: ISRCTN58249751<br>Registered on 29/04/2022<br>Link: https://www.isrctn.com/ISRCTN58249751
**Protocol version {3}**	3 – 29/03/2023
**Funding {4}**	The study is funded by the Nuffield Foundation: FR-000022056
**Author details {5a}**	Centre for Brain and Cognitive Development, Birkbeck, University of London<br>Department of Psychology, University of Bath<br>Institute of Psychiatry, Psychology & Neuroscience, King's College London
**Chief Investigator**	Professor Tim Smith
**Co-applicants**	Dr Rachael Bedford

	Dr Hannah Pickard<br><br>Claire Essex
**Trial Statistician**	Dr Ben Carter<br><br>Petrina Chu
**Name and contact information for the trial sponsor {5b}**	Birkbeck, University of London.<br><br>Malet Street,<br><br>London,<br><br>WC1E 7HX
**Role of sponsor {5c}**	The sponsor will have no role in study design, collection, management, analysis or interpretation of data, report writing or decision to submit the report for publication.
**Role of the Funder**	The funder will have no role in study design, collection, management, analysis or interpretation of data, report writing or decision to submit the report for publication.

Introduction

Background and rationale {6a}

Childhood screen time (e.g., TV, videogames, smartphones) is increasing[2] and has been linked to changes in various developmental factors including toddler sleep[3] and attention[4,5], two critical determinants of childhood health and cognitive function. Understanding the potential causal influence of screen time on toddler sleep and cognitive development (e.g., attention) is thus of the highest importance. Recent screen time evidence-reviews led to specific UK recommendations of no screen time before bed (Royal College of Pediatrics and Child Health, RCPCH). However, there is a critical need for causal evidence on the impact of screen time before bed on development. Previous parent/child education interventions have demonstrated decreased screen time for school-aged children[6] but similar Randomised Control Trials (RCT) have not been run with toddlers nor tested the associated impacts on development. Unlike for older children, parents are the gatekeepers of screen time for toddlers (e.g., handing the child a smartphone, switching on the TV) so the potential to change a toddler's screen exposure via parent-education is high and may establish a family's long-term screen time culture. Here, we propose a feasibility and pilot trial of parents intervening on toddlers' screen time in the hour before bed. This recommendation has cross-agency consensus (World Health Organisation (WHO), RCPCH, CMO, American Academy of Pediatrics (AAP)[7]).

The negative association between screen exposure and sleep problems[8] is greatest immediately before bed[9]. Our Toddler Attention Behaviour and LEarning with Touchscreens (TABLET) project was the first to demonstrate this association during

toddlerhood[3]. The correlational associations between screen time and sleep problems have also been confirmed by interventions in adults with matched screen/non-screen content, showing direct causal impact of screens on sleep[10]. Sleep is vital for brain maturation and clearance of neurotoxins and insufficient quality sleep has an important impact on child development[11]. Poor sleep is also bidirectionally associated with a child's ability to focus attention: reduced concentration may follow insufficient sleep[12], as well as difficulties falling/staying asleep being higher in children with attention problems[13].

In the first two years of life, developing attentional control abilities are early precursors of later executive function, skills essential for educational success[14]. Exposure to intense screen content has been immediately[15] and habitually associated with later attention deficit hyperactivity disorder (ADHD) traits[16]. The cognitive resources required to inhibit distraction are most depleted in the evening, making it harder for children to ignore background screens. In our TABLET study, the use of novel, gaze-contingent experimental methods allowed us to precisely measure toddler attention, and our recent findings from 18-month-old high-touchscreen users shows an attention profile of enhanced saliency driven attention and reduced goal-directed driven attention[4,5].

Screen exposure may act directly to reduce sleep (e.g., melatonin suppression by blue light[10]) and attention control (e.g. executive attention suppression[15]), or indirectly, through displacement of other healthy bedtime activities (e.g. play/story-time, bath[17]). Teasing apart these hypotheses requires comparison between an

intervention that alters screen time before bed and one that encourages routine pre-bedtime activities without altering screen time.

Intervention description {11a}

<u>PASTI Arm</u>

The 7-week Parent Administered Screen-Time Intervention (PASTI) requires caregivers to remove screen time from their child (range 17 – 31 months) in the hour before bed. The PASTI is modelled on effective parent-education screen time interventions in older children (e.g. a 7-week intervention by Dennison et al.,[6,18]). A cover story for PASTI will be used to obscure the role of screen time to avoid self-selection of families interested in the topic and to minimise bias in caregiver-reported screen use across the intervention arms. As such, families will be recruited into our “Bedtime Boost” trial on toddler bedtime activities in the hour before bed. All families will be pre-screened to assess their eligibility to take part in the trial (see the Eligibility Criteria {10} section below for further details).

PASTI materials were created through an iterative process involving two co-creation workshops with Early Years Stakeholders (e.g. parent practitioners) and a focus group with caregivers of a one-year-old infant. At the start of the intervention, families will receive a booklet to educate them on “No screen time in the hour before bed” and a Family Bedtime Box with tips on alternative pre-bed activities to help them displace screen time in the hour before bed (e.g., activity cards with fun bath games, story time ideas, as well as a selection of age-appropriate toys, such as crayons, a bath toy, puzzle etc.). The activity cards for the PASTI arm contain a no

screen time symbol, as well as suggested times for each activity card to aid with displacing screen time in the hour before bed. Caregivers will be asked to use toys/activities from the bedtime box with their infant in the hour before bed. Every week, caregivers will receive a check-in message that contains suggested activity card ideas to promote continued participation in the trial.

During the first week of the intervention, families will be invited to take part in a phone/video call with an unblinded researcher to reflect on their strategies for removing screen time in the hour before bed and any challenges that they may face/have faced. Throughout the intervention, caregivers will be asked to complete a short daily Screen Time Questionnaire that will assess their infants use of screens in the hour before bed (see Appendix A). This questionnaire will capture their infant's duration of screen use in the hour before bed, and if screen use is reported, follow-up questions regarding the mode and reason for the screen use will be examined. Caregivers will also be asked to complete a bi-weekly (weekday and weekend) Bedtime Activity Diary to capture their infant's activities in the hour before bed (e.g. screen use, play), which will also include questions about their infant's sleep patterns in the last week using 4 items from the BISQ-R questionnaire[19], and caregiver anxiety using a 6-item state version of the STAI[20] (see Appendix B).

Explanation for choice of comparators {6b}

To examine the direct impact of screen exposure in the hour before bed on childrens' sleep and attention control, the PASTI Arm will be compared to two other modes of delivery.

Bedtime Box Arm

Families will receive identical materials (i.e. Family Bedtime Box) to the PASTI Arm, but ***without any screen time guidance.*** In addition, the selection of activity cards will not contain a screen time symbol or a time suggestion for each activity. Caregivers will be asked to use toys/activities from the bedtime box with their infant in the hour before bed. Every week, caregivers will receive a check-in message that contains suggested activity card ideas to promote continued participation in the trial. Throughout the intervention, caregivers will be asked to complete a bi-weekly (weekday and weekend) Bedtime Activity Diary to capture their infant's activities in the hour before bed (e.g. screen use, play), which will also include questions about their infant's sleep patterns in the last week using 4 items from the BISQ-R questionnaire[19], and caregiver anxiety using a 6-item state version of the STAI[20] (see Appendix B). Families in the Bedtime Box Arm will have no other contact with the researchers throughout the trial.

No Intervention Arm

Families will not receive the Family Bedtime Box or any instructions to change their child's activities in the hour before bed or avoid screen time. We expect pre-bedtime activities (such as feeding, playing, being read to, bathing and watching TV[11]) to continue as usual. Throughout the intervention, caregivers will also be asked to complete a bi-weekly (weekday and weekend) Bedtime Activity Diary to capture their infant's activities in the hour before bed (e.g. screen use, play), which will also include questions about their infant's sleep patterns in the last week using 4 items from the BISQ-R questionnaire[19], and caregiver anxiety using a 6-item state version

of the STAI[20] (see Appendix B). Caregivers in the No Intervention Arm will not receive weekly check-in messages and will have no other contact with the researchers throughout the trial. At the end of the trial, caregivers in the No Intervention Arm will receive a Family Bedtime Box with the toys and activity cards used for the Bedtime Box Arm.

Objectives {7}

The primary aim of the trial is to assess the feasibility of implementing a Parent-Administered Screen Time Intervention (PASTI), compared with a Bedtime Box Intervention and No Intervention, in toddlers who have parent-reported screen time in the hour before bed. To fulfil our primary aim, we will:

1. Assess the feasibility of conducting PASTI, including participation rate, participant randomisation, participant attrition, and intervention adherence.
2. Gain insight into parent experiences of PASTI and any barriers to adherence.
3. Estimate statistical parameters of the primary outcomes necessary to power a future large-scale, confirmatory trial, e.g. means and standard deviations of continuous outcome measures: total night-time sleep duration (actigraphy); exogenous attention control (singleton search saccadic reaction time from the Visual Search Task).

Our secondary aims and objectives are to collect preliminary effect size estimates for PASTI's impact on toddler sleep and attention, and collect preliminary effect size estimates for PASTI's impact on secondary sleep (e.g. average nap duration, frequency of night awakenings), attention (e.g. exogenous and endogenous attention

control) and screen time outcomes (e.g. average toddler screen use in the hour before bed).

Trial design {8}

This study is a three-arm randomised controlled trial in toddlers. Participants will be randomised to either the PASTI Arm, Bedtime Box Arm or No Intervention Arm, and will complete measures at baseline and follow up (weeks 6 and 7) home and lab assessment. A blinded assessor will collect baseline and follow up measures.

Methods: Participants

Study setting {9}

The study is set within the Centre for Brain and Cognitive Development (CBCD) Babylab and in families' homes. All baseline and follow up lab assessments will take place in the CBCD Babylab, and all baseline and follow up home assessments and the intervention will be completed in each family's home.

Eligibility criteria {10}

Inclusion criteria

After completing the pre-screen assessment, participants must meet the following criteria prior to randomisation:

1. A family with an infant between 16 and 30 months at pre-screening (enrolment).

2. A family that lives in Central/Greater London and surrounding areas (within 75 miles of the Birkbeck Babylab).
3. A family that reports that their infant uses screen time in the hour before bed at pre-screening. They must report that their infant uses ≥ 10 minutes of screen time in the hour before bed on ≥ 3 days of the week.
4. A caregiver is able to provide informed consent.

Exclusion criteria

1. A family with an infant who has a parent-reported genetic or neurological condition (e.g., Downs Syndrome).
2. A family with an infant who was born prematurely (< 37 weeks).
3. A family with an infant who is taking part in another trial or research study.

Recruitment {15}

Recruitment into the Bedtime Boost Trial will begin in spring 2022 and is expected to be completed by summer 2023. The target sample size is 105 families with a child aged 17 - 31-months old who has screen time in the hour before bed, with 35 recruited into each intervention group. Families will be recruited via several routes. Each route will advertise our trial using a physical/online poster that contains a link where families can sign up to take part.

a) The Early Years Alliance (EYA) will advertise our trial across their social media platforms (e.g. Facebook, Twitter, Instagram), their pre-schools/baby groups/child centres across London and their membership database.

b) The National Childbirth Trust (NCT) will advertise our trial across their social media platforms (e.g. Facebook, Twitter, Instagram) and through their membership database.
c) The Sleep Charity will advertise our trial across their social media platforms (e.g. Facebook, Twitter, Instagram).
d) Our trial will be advertised through the Centre for Brain and Cognitive Development (CBCD) Babylab Database.
e) Our trial will be advertised through various social media platforms (e.g. Facebook, Instagram, Twitter).

Recruitment will take place in tranches (i.e. April 2022, July 2022, November 2022 and March 2023). We will use probabilistic sampling based on the English Index of Multiple Deprivation (IMD) quintiles to target families across the full range of socioeconomic status (SES). Our partnering trusts and charities will be regularly reminded about the trial and posters made available.

<u>Methods: Interventions</u>

Criteria for discontinuing or modifying allocated interventions {11b}

Adverse events will be monitored and recorded. Adverse events will be monitored through a form embedded within our arm-specific participant web resource. Participants will be directed to this form via the frequently asked questions (FAQ) resource and directly by the researchers if necessary. Through this form families will be able to report adverse events directly to the research team and withdraw from the trial at any point. Furthermore, families will be asked to report any changes/concerns that have arisen during their participation in the trial when completing the online

debrief questionnaire. Families will be able to request to speak to an unblinded researcher in extreme circumstances.

Strategies to improve adherence to interventions {11c}

Intervention adherence will be monitored throughout the trial by tracking family's completion of intervention assessments, including daily caregiver-reported screen use in the hour before bed (PASTI Arm). The number of questionnaires completed will be recorded. Families will be sent automated reminders to complete questionnaires, with a maximum of two reminders per questionnaire sent. To improve intervention adherence, families (PASTI and Bedtime Box Arm) will also receive a Family Bedtime Box filled with toys and alternative pre-bed activities to use with their child. The Family Bedtime Box will act as an aid to support the removal of screen time in the PASTI Arm. Families (PASTI and Bedtime Box Arm) will also receive weekly check-in messages, which will contain activity card ideas. All arms will also have access to FAQs to support their continued participation in the trial. At the end of the trial, families in the PASTI Arm and Bedtime Box Arm will take part in an online debrief questionnaire to examine their experience of taking part in the trial. A subset of families from the PASTI Arm will also take part in a semi-structured interview to gain an in-depth view of their experiences.

Relevant concomitant care permitted or prohibited during the trial {11d}

Concomitant involvement in another trial or research study is prohibited during the trial.

Methods: Outcomes

Please refer to "Plans for assessment and collection of outcomes" for full details of the study outcome measures.

Primary feasibility outcomes:

(i) **Participation rate:** A CONSORT diagram will represent the number of families who consented, are eligible (based on pre-screener), probabilistically sampled (based on IMD quintiles) and randomised. We will examine the number of eligible participants by recruitment strategy (Centre for Brain and Cognitive Development (CBCD), National Childbirth Trust (NCT), Early Years Alliance (EYA), The Sleep Charity), and success of probabilistic sampling by comparing the socioeconomic profile of families enrolled relative to geographic norms.

(ii) **Intervention adherence:** Intervention adherence will be based on data collected over a period of 6 weeks (week 1 – week 6) during the intervention. For exploratory purposes adherence data will continue to be collected until either a) the follow up lab visit; or b) 2 weeks after the participants scheduled follow up lab visit; whichever is sooner. A quantitative measure of adherence to PASTI will be collected daily using 1) a short Screen Time Questionnaire specifically referring to the number of minutes of screen time in the hour before bed; and 2) the bi-weekly Bedtime Activity Diary. These questionnaires will be automatically delivered to the parents' phones in the evening. In the PASTI Arm this will ask the duration their child was exposed to a screen in the hour before bed (note, this is evening bedtime only, not daytime naps). In the PASTI Arm, a binary success (no screen time) vs. fail will be collected and collated by the unblinded researcher for trial steering committee adherence

monitoring. In the PASTI Arm, we will calculate and describe the average proportion of days with no screen time in the hour before bed, and the average duration of screen use in the hour before bed. These metrics will determine the design features for a future confirmatory study.

(iii) **Participant retention:** Participation and data completion rates at the follow up lab timepoint.

(iv) **Participant experiences:** Acceptability of PASTI for PASTI Arm participants will be determined through quantitative (e.g. frequency and proportion of satisfaction rating responses) and qualitative investigation by examining experiences, including any benefits/challenges of taking part.

(v) **Assessment acceptability:** Acceptability of assessment measures to PASTI Arm participants, which will be determined through quantitative (e.g. frequency and proportion of satisfaction rating responses) and qualitative investigation at the end of the trial.

Metrics of feasibility study acceptability

We have chosen to implement a ‘traffic light’ system to assess the feasibility of implementing a full-scale PASTI trial. Performance metrics that fall in the Red zone indicate that a full trial following the current design may not be feasible, metrics that fall in the Amber zone indicate that a full trial may be feasible but the protocol should be modified or the situation monitored closely, and metrics that fall in the Green zone indicate that a full trial is feasible and we may continue without modifications to the current study design.

Table 1. Traffic light system to assess PASTI feasibility

Metric	Red zone	Amber zone	Green zone
**Randomisation**<br>(Number of participants randomised overall)	≤ 73	74 to 104	≥ 105
**PASTI daily questionnaire completion**<br>(% of participants randomised to PASTI and retained to lab follow up that complete ≥ 60% of daily screen time questionnaires)	< 65%	65% to 79%	≥ 80%
**PASTI adherence to screen time removal (week 1 – week 6)**<br>(% of participants randomised to PASTI that report no screen time on ≥ 60% of daily screen time questionnaires completed)	< 50%	50% to 69%	≥ 70%
**Retention**<br>(% of randomised participants attending follow-up Lab visit)	< 70%	70% to 74%	≥ 75%
**PASTI debrief questionnaire completion**<br>(% of participants randomised to PASTI that complete the debrief questionnaire measuring participant experience and assessment acceptability – see section {20a})	< 65%	65% to 74%	≥ 75%

Secondary outcomes:

Estimate the following at week 6 and 7 follow up:

Preliminary PASTI effect sizes on

i. Total infant night-time sleep duration, using actigraphy.

ii. Exogenous attention control (single search saccadic reaction time from the Visual Search Task).

iii. Average toddler screen use (minutes) in the hour before bed, measured through an average of one weekday and one weekend bi-weekly bedtime activity diary directly preceding the lab visits.

iv. *Additional sleep outcomes:* average nap duration using actigraphy, frequency of night awakenings using actigraphy, sleep efficiency (defined as the total night time sleep expressed as a percentage of time in bed) using actigraphy, and parent-reported sleep onset latency from the Brief Infant Sleep Questionnaire – Revised (BISQ-R).

v. *Additional attention control outcomes:* prosaccade saccadic reaction time and proportion of antisaccades in the Antisaccade Task, baseline saccadic reaction time and disengagement saccadic reaction time from the Gap-Overlap Task, and parent-reported effortful control and the inhibitory control subscale from the Early Childhood Behaviour Questionnaire (ECBQ).

Participant timeline {13}

Please see the participant timeline outlined below. Continuous measures are marked with a continuous line. Please see our sample CONSORT diagram in Appendix C.

	STUDY PERIOD												
	Prescreen	Enrolment		Allocation	Post-allocation							Close-out	Adverse events
**Timepoint**	**(-)tx***	**(-)t1**	**t0**	**t0**	**t1**	**t2**	**t3**	**t4**	**t5**	**t6**	**t7**	**~ t8+**	**Anytime**
**ENROLMENT (ALL ARMS):**													
Informed consent (stage 1 and 2)	X												
Eligibility screening	X												
Probabilistically sampled	X												
Allocation (randomised)				X									
**INTERVENTIONS:**													
PASTI Arm					X	X	X	X	X	X	X		
Bedtime Box Arm					X	X	X	X	X	X	X		
No Intervention Arm					X	X	X	X	X	X	X		
**HOME ASSESSMENTS (ALL ARMS):**													
Daytime Activity Levels Questionnaire		X								X			
Vineland Adaptive Behavior Scale (VABS)		X								X			
Brief Infant Sleep questionnaire (BISQ)		X								X			
Early Childhood Behaviour Questionnaire (ECBQ)		X								X			
State and Trait Anxiety Inventory (STAI)		X								X			
Sleep Actigraphy Watch		X	X							X	X		
Sleep and Motion Watch Diary		X	X							X	X		
**LAB ASSESSMENTS (ALL ARMS):**													
Mullen Scales of Early Learning			X								X		
Visual Search Task			X								X		
Gap-Overlap Task			X								X		
Antisaccade Task			X								X		
**INTERVENTION ASSESSMENTS:**													
Caregiver Daily Screen Time Questionnaire (PASTI Arm only)					X	X	X	X	X	X	X		
Bedtime Activity Diary (All Arms) - including 6-item STAI and 4-item BISQ		X	X		X	X	X	X	X	X	X		
Parent Debrief Questionnaire (PASTI and Bedtime Box Arm only)												X	X
Parent Debrief Interview (PASTI Arm only)												X	

* week < -t1

Sample size {14}

In line with guidance on feasibility studies, no power calculation has been carried out[21]. To estimate key unknown parameters necessary to inform the design of a full RCT (e.g., participation rates, attrition, intervention adherence), a sample size of 60 to 100 has been shown to be sufficient in a feasibility trial[22]. A target sample size of 105 (N=35/arm) will be recruited.

Methods: Allocation

Sequence generation {16a}

The King's Clinical Trials Unit (KCTU) web-based randomisation system based at King's College London will be used to generate the allocation sequence. The randomisation system will be designed by the trial management group and will be implemented by the KCTU for the duration of the project. It will be hosted on a dedicated server within King's College London. The sequence will be generated using an equal 1:1:1 allocation ratio to one of the three trial arms. To ensure balance between trial arms, the randomisation system will utilise minimisation with respect to child sex, socioeconomic status and child age at randomisation (17-24.4 months old vs 24.5-31 months old; binary variable). Socioeconomic status (categorical variable; 1-5) will be derived from the Index of Multiple Deprivation (IMD) quintiles using the household postcode for families.

Concealment mechanism {16b}

Trial arm allocation will be generated using a web-based randomisation sequence and the trial team will be masked for the sequence. The chief investigator (CI) or

delegate will request usernames and passwords from the KCTU. System access will be strictly restricted through user-specific passwords to the authorised research team member (the unblinded researcher responsible for randomisation). It is a legal requirement that passwords to the randomisation system are not shared, and that only those authorised to access the system are allowed to do so. If new staff members join the study, a user-specific username and password will be requested via the trial management group from the KCTU team and a request for access to be revoked will be submitted when staff members leave the project.

Implementation {16c}

Baseline lab measures will be collected by blinded research staff. After baseline lab measures have been collected, a blinded researcher will be removed from the room to ensure that they remain blind to the randomisation process. Following this, the unblinded researcher will input the minimisation factors into the KCTU web-based randomisation system to determine trial arm allocation. Information on minimisation factors (child age, SES, child sex) will be stored in a secure user-controlled area, and access to the data must be requested through a controlled process. Participant email addresses, names and full postcodes will not be entered into the randomisation system. No data will be entered into the randomisation system unless a participant has signed a consent form to participate in the trial. Upon randomisation, an email confirmation will be generated and sent to the blinded trial researcher without details of trial arm allocation. Only the unblinded research staff member will receive notification of trial arm allocation in order to inform participants of their study intervention and provide intervention materials at the end of the baseline lab visit. A full audit trail of data entry will be automatically date and time

stamped, alongside information about the user making the entry within the system. The follow-up lab assessments will be conducted by a blinded researcher. Upon request, KCTU will provide a copy of the final exported dataset to the appropriate research staff member in .csv format.

<u>Methods: Blinding</u>

Who will be blinded {17a}

For each allocation to the PASTI, Bedtime Box or No Intervention Arm, the participant and the unblinded researcher, who delivers the intervention materials, will be aware of the arm allocation. After the baseline lab visit, the blinded researcher will be removed from the room to ensure blinding to the group allocation is maintained. The blinded researcher will not have access to the allocation database. The blinded researcher will collect measures at the follow up lab visit. The unblinded researcher will coordinate the debrief online questionnaires and conduct the debrief qualitative interview, as it is likely that information shared would lead to unblinding. The Junior Statistician will be blind to group allocation until after the initial statistical analysis plan is approved. The PI and Co-Is will be blind to the group allocation until the end of the trial after the database lock.

Procedure for unblinding if needed {17b}

Table 2. Procedure for unblinding the PASTI trial team

**Trial Role**	**Blinding Status**	**Additional details**
Chief Investigators	Blinded	To be blinded until review of trial report.
Senior Trial Statistician	Blinded	To be blinded until review of final database extract after database lock.
Junior Trial Statistician	Unblinded	To be blinded until approval of statistical analysis plan by the TSC.
Postdoctoral Research Associate (PDRA) – Baseline outcome assessor	Unblinded	PDRA will perform initial baseline activities blinded and then randomise the participants, thus becoming unblinded to group allocation.
Trial Steering Committee (TSC)	Blinded	All members will receive open reports during regular updates given by the PDRA/RA/Junior Trial Statistician. The TSC Chair can request a closed unblinded report if there are safety concerns.
Baseline and follow up outcome assessor (e.g. Research Assistant (RA) and students)	Blinded	Testing will be done blind to group allocation.

Trial Participants	Unblinded	Participants will be informed of their group allocation by the unblinded researcher at the end of the baseline lab visit.

Methods: Data collection, management, and analysis

Plans for assessment and collection of outcomes {18a}

Pre-screen Online Questionnaire

This is a caregiver-reported, online questionnaire that will be used to capture family demographics (e.g. child age, sex, ethnicity, caregiver education), activities in the hour before bed (including screen time) and medical conditions. Household postcode captured within the consent form will be used to deduce a metric of social economic status (SES) using the Index of Multiple Deprivation. This questionnaire will be used to screen participants for inclusion into our PASTI (see Appendix D).

Baseline and follow-up home assessments

Baseline home assessments will be collected by blinded researchers prior to the assignment of participants to the trial arm. These will take place over a two-week period. Baseline questionnaire measures will be completed by a caregiver via a secure online survey platform (e.g. Redcap). At the start of the baseline home assessment, caregivers will be asked to complete the questionnaires outlined below. These questionnaires must be completed before being randomised into an intervention group at the baseline lab assessment.

- **Brief Infant Sleep Questionnaire – Revised (BISQ-R[19]).** This is a caregiver-report measure designed to capture infant sleep patterns and bedtime routine.
- **Daytime Activity Levels Questionnaire.** This is a caregiver-report measure designed to capture infants' daily activities, including screen time. Please see Appendix E.
- **Early Childhood Behaviour Questionnaire – Very Short Form (ECBQ[23]).** This is a caregiver-report measure of infant temperament. The effortful control and inhibitory control subscales from the ECBQ short form will also be added.
- **Vineland Adaptive Behaviour Scales (VABS[24]).** This is a caregiver-report measure designed to capture broad domains of adaptive functioning (e.g. communication, socialisation and motor skills).
- **State and trait anxiety inventory (STAI[25]).** This is a caregiver-report measure designed to capture current caregiver anxiety.

During the baseline home assessment, caregivers will also be asked to complete an online bi-weekly **Bedtime Activity Diary** for their child. This is a caregiver-report measure designed to capture an infant's activities in the hour before bed (e.g. screen use, play). This measure will be used to capture the types of activities the infant engages in, as well as the duration and mode of use for some activities, if selected. In addition, the weekday Bedtime Activity Diary will contain a **6-item state version of the STAI**[20] to capture caregiver anxiety throughout the trial. Furthermore, the weekend bedtime activity diary will contain **4 items from the BISQ-R questionnaire** to capture the child's sleep patterns over the past week. Please see Appendix B.

Finally, caregivers will be asked to capture a measure of their infant's physical activity and sleep over a 7- to 9-day period using an **Actigraphy Watch** (MotionWatch8, CamNtech, Cambridge, UK). Actigraphy is a reliable and valid method for unobtrusively and naturalistically assessing sleep/wake cycles in children, with >80% agreement with overnight polysomnography[26]. Caregivers will be asked to fit the actigraphy watch to their infant's ankle to gather physical activity and sleep data. The watch is lightweight, waterproof, body-worn and cable-free, and safe to use for a prolonged time. Alongside this, caregivers will be asked to complete a short **Sleep and Motion Watch Diary** each day their child wears the actigraphy watch to report on their child's sleep routine (e.g., when their child napped/slept) and whether the watch was removed (see Appendix F).

All baseline home assessments, including online questionnaire, bedtime activity diary, and physical and sleep activity logging via the actigraphy watch will be repeated at the follow-up home assessment. The follow-up home assessments will take place during weeks six and seven of the trial (please see the participant timeline for further details).

Baseline and follow up lab assessments

The Centre for Brain and Cognitive Development (CBCD) within the Department of Psychological Sciences, Birkbeck, University of London, will be the coordinating site and location for lab-based assessments. Baseline lab assessments will be collected by blinded researchers prior to the assignment of participants to the trial arm. Follow-up lab assessments will be collected by the blinded researcher during week seven of

the trial, with a two-week visit window for lab assessments. During the baseline and follow-up lab assessment, caregivers will bring their infant to the Birkbeck Babylab where they will take part in several gaze-contingent eye-tracking experiments using an EyeLink 1000 Plus (https://www.sr-research.com/eyelink-1000-plus/) and Experiment Builder on a Windows PC. Stimuli will be presented on a 24” widescreen monitor with stereo speakers via custom scripts using Experiment Builder. The infant will be sitting on the parent’s lap approximately 60cm from the screen. Participants will be video-recorded using a web camera, for data quality considerations. The eye-tracking experiments will be administered in the following order and include:

1. ***Visual Search Task.*** The task was adapted from Kaldy and colleagues[27] and has been used to measure individual differences in visual attention and perception in toddlers. In this gaze-contingent version[4] the subject is presented with a search array until they fixate on the target (a red apple) or until 4 seconds has elapsed. The array can be a single feature array (mix of red and blue apples; set sizes 5 and 9), or a conjunction array (mix of red and blue apples and cropped apples; set sizes 5, 9 and 13). Some arrays will also contain a salient distractor stimulus (e.g. bright blue apple). To grab participants’ attention and guarantee fixation at the centre of the screen, each trial starts with the target ‘flying in’ (800ms). When the infant attends to the central target it fades (750ms) and the trial array is presented. At the end of the trial the target spins and an audio reward is played (clapping) (1300ms). Trials will be presented continuously, grouped into three blocks: (1) 3 single feature arrays, fixed order; (2) 1 single feature array, 9 conjunction arrays, randomised; (3) 4 single feature arrays, 9 conjunction arrays, randomised; and (4) 8 single feature distractor arrays, randomised. Saccadic reaction

times to look at the target stimulus (red apple) for each correct trial will be summed and divided by the total number of correct trials, for each set size (5, 9 and 13), array type (single – one feature search, exogeneous attentional control; conjunction – two feature search, endogenous attentional control) and distractor type (salient distractor vs no salient distractor).

2. ***Antisaccade Task.*** In this gaze-contingent anti-saccade paradigm[5] the infant is encouraged to look to a position located in the opposite direction of the visual cue being displayed. Each trial starts with the presentation of a central animation (i.e. a star) to attract the infant's attention to the centre of the screen. Once the infant looks at the central stimulus (for 500ms), a distractor stimulus (black circle) will appear on the left or right side of the screen for 200ms. 1000ms after the distractor disappears a target stimulus (red circle) will appear on the opposite side of the screen. If the infant looks at the target stimulus, an attractive animal animation with sound will replace it (2500ms) and the trial will end. If the infant looks at the target side before the target appears, then the animation automatically plays. If the infant doesn't look at the target/target side no animation plays and the trial ends after 2000ms. The task will be presented as a series of 2 consecutive blocks of 15 trials. Within participant, the distractor and target stimulus position (left/right) is fixed per block, and randomised across participants. In each trial, we will determine (1) whether the infant looked at the distractor and (2) whether the infant looked at the target location before the target stimulus onset (anticipatory looking). The following metrics will be calculated across all trials: (1) the proportion of looks towards the distractor not followed by an anticipatory look (i.e. pro-saccades); (2) the proportion of looks towards the distractor followed by an anticipatory

look (i.e. corrective saccades); (3) the proportion of looks to the target in the absence of looks to the distractor (anti-saccades, where inhibition of pro-saccades, as well as the production of contralateral saccades is required); and (4) average saccadic latency for prosaccades.

3. ***Gap-overlap Task.*** The Gap-Overlap Task contains six blocks of 11 trials interleaved with free-viewing of static and dynamic scenes. In this task a central stimulus is presented followed by a peripheral target that appears either on the left or the right-hand side[5]. All trials begin with a central stimulus animation to attract the infant's attention to the centre of the screen. Once the infant has fixated on the central stimulus, a peripheral target will be presented to the left- or right-hand side of the screen. There are three different trial conditions: (1) baseline trials, where the peripheral target appears immediately after the central stimulus disappears; (2) gap trials, where the central stimulus disappears and is followed by a gap (200ms) before the peripheral target appears; and (3) overlap trials, where the central stimulus remains on the screen when the peripheral target is also present. For 10% of trials the peripheral target will be presented either on bottom or on top of the central stimulus to avoid anticipated looking (vertical trials), however, these will not be used for analysis. When the infant looks at the peripheral target or after 4 seconds has elapsed, a novel animated stimulus will play (reward stimulus) and the trial will end. All trial conditions will be presented pseudo-randomly within block: 30% of the trials will be baseline trials, 30% of the trials will be gap trials and 40% of the trials will be overlap trials. A maximum of 60 trials (ignoring the vertical trials) will be presented. The central stimulus and background colour will change for every block. Saccadic latencies are defined

as the time from the presentation onset of the peripheral target to the first look to the peripheral target. The following outcome metrics will be calculated: (1) Baseline, by looking at the average saccadic latency on baseline trials; (2) Overlap, by looking at the average saccadic latency on overlap trials; (3) Gap, by looking at the average saccadic latency on gap trials; and (4) Disengagement, by subtracting the baseline latency from the overlap latency.

During the lab assessment, infants will also be assessed using the **Mullen Scale of Early Learning**[28] to examine global development in language, motor and perceptual abilities. The Mullen Scale of Early Learning include five scales: Visual Reception, Gross Motor, Fine Motor, Expressive Language and Receptive Language. All scales will be administered to gain a measure of full scale IQ. Parents will also be asked about any medication their child takes to help them sleep.

<u>Intervention based assessments</u>

Throughout PASTI, participants in all arms (PASTI Arm, Bedtime Box Arm and No Intervention Arm) will be asked to complete a bi-weekly **Bedtime Activity Diary**, which is identical to the measure used in the baseline and follow up home assessments (see Appendix B). Participants in the PASTI Arm will also be asked to complete a short daily **Screen Time Questionnaire** to assess their infant's use of screen time in the hour before bed (see Appendix A). This questionnaire will capture their infant's duration of screen use in the hour before bed, and if screen use is reported, follow up questions regarding the mode and reason for the screen use will be examined.

The intervention will continue to be followed until either a) the follow up lab visit; or b) 2 weeks after the participants scheduled follow up lab visit; whichever is sooner.

<u>Qualitative Debrief Questionnaire and Interview</u>

At the end of the intervention, all caregivers in the PASTI and Bedtime Box Arm will be asked to take part in an online **Debrief Survey** to examine their thoughts, feelings, and experiences of taking part in the trial. A series of questions will be asked to capture a) caregivers' confidence with implementing the intervention, b) whether parents felt the intervention was feasible (e.g. removing screen time in the hour before bed), and c) whether parents experienced any challenges/barriers taking part in the intervention. All questions regarding experiences of removing screen time will be removed from the questionnaire given to the Bedtime Box Arm. A random subset of families (n=15) in the PASTI Arm will also be asked to take part in a semi-structured **Debrief Interview**. The interview will be conducted via Microsoft Teams or over the phone and will involve a set of pre-specified questions with follow-up prompts to gather in-depth information regarding their views and experiences related to the trial feasibility.

Plans to promote participant retention and complete follow-up {18b}

Participants who complete the pre-screen questionnaire but are not eligible to take part in the study will be entered into a prize draw to win a £100 voucher. Participants who are eligible and attend the baseline lab visit will be given a £30 voucher and reimbursed for their travel expenses. Participants who attend the follow-up lab visit will be given a further £50 voucher and reimbursed for their travel expenses. All

participants will be encouraged to take part in the final follow-up home and lab-based assessments regardless of treatment completion. All participants who complete the follow-up assessments will also be given the Family Bedtime Box (if they have not received one as part of the trial; worth approximately £13 per box), a Babylab t-shirt or bag, and a children's recipe book from one of our charity partners (The Sleep Charity) as a thank you for participating. Furthermore, to promote completion of trial home-based assessments (e.g. online questionnaires) reminders will be sent via SMS, with a maximum of two reminders per questionnaire.

Data management {19}

Data collection and processing

Participant consent will be collected in two stages: 1) participants will consent to completing our pre-screen questionnaire; and 2) if eligible, participants will consent to take part in our trial. The participant consent and pre-screen questionnaire will be completed electronically (via REDCap). All online home-based questionnaire data will be collected via REDCap and linked with the participants unique ID number. Questionnaire data will be processed locally on a password-protected computer and subsequently exported and stored on the secure Birkbeck data server. Once the questionnaire data has been exported, we will remove it from the survey platforms servers. The sleep and motion watch diary will be completed by the participant using pen and paper, and the diary will be given to the research team during the lab visit. The diary will be scanned and stored on the secure Birkbeck data server. Once the diary has been electronically stored, the paper copy will be securely destroyed (e.g. shredded and put in a secure paper disposal bin). Experimental data and physical

activity and sleep logging data will be processed locally on a Windows testing laptop (with an encrypted hard disk, password protected and stored within a locked lab space) and linked with the participants unique ID number. Once the data has been processed it will be exported and stored on the secure server. No questionnaire or experimental data will be stored locally apart from during processing. All data access will be restricted to the research team.

Qualitative interview data will be collected for a subset of participants in the PASTI Arm. Participants will be interviewed via Microsoft Teams or over the phone and their discussion will be video/audio recorded, depending on the mode of delivery for the interview. The video/audio recording will be transferred immediately onto the secure Birkbeck data server. Transcripts of the interview audio recordings will be generated, and after removing all identifying information these will be uploaded onto the secure Birkbeck data server. Once the audio data files have been transcribed, all audio files will be deleted. The transcripts will be securely shared with Flow Associates, a partner company who are supporting qualitative data analysis, via Birkbeck secure OneDrive.

PASTI Arm participants will be asked to complete online daily adherence questionnaires throughout the intervention period. PASTI Arm and Bedtime Box Arm participants will be asked to complete a debrief survey at the end of trial participation. Data from these online questionnaires will be processed by the unblinded researcher and stored in a separate password-protected folder on the

secure Birkbeck data server. Only the unblinded researcher will have access to this folder during study enrolment.

Data checks and double entry into a separate research database

As mentioned above, source questionnaire and experimental data will be collected and stored electronically. During data processing, designated blinded study researchers will perform range and valid value checks. Access to the secure Birkbeck data server will be restricted to Birkbeck project researchers.

Processed and pseudo-anonymised data (excluding the questionnaires completed only by the PASTI Arm participants) will be entered by designated Birkbeck researchers into a separate REDCap research database that can be accessed by external collaborators for data analysis purposes (BC and PC at King's College London; RB at University of Bath; Flow Associates). Access to this research database will be read-only for external collaborators and restricted via user-specific passwords. After the statistical analysis plan has been approved, the Junior Statistician will get read-only access to pseudo-anonymised PASTI Arm data from daily adherence questionnaires, and PASTI Arm and Bedtime Box Arm debrief survey data.

The pseudo-anonymised data entered in this research database will contain the participant's unique pin generated by the randomisation software as the unique identifier. The unique pin from the KCTU randomisation system will be used in these files, instead of the unique ID number created by the participant, to add a layer of

security when sharing the data across institutions, as the unique pin and participant identifying information (e.g. name, email) are never stored together.

Data storage and extraction

Processed data is stored on secure Birkbeck servers. The pseudo-anonymised data entered in the REDCap research database will also be stored on secure Birkbeck servers. A copy of the REDCap research database will be made for back-up purposes every three months and stored on the secure Birkbeck server and an encrypted external hard drive. At database lock, a copy of the research database will be extracted by the trial statistician for analysis. The trial statistician will also request a randomisation extract from KCTU, along with the final dataset for PASTI Arm participant questionnaires. After the primary analysis has been completed, a pseudo-anonymised dataset will be retained at Birkbeck for a period of 10 years. After 10 years, personal data (e.g. name, email address) will be deleted and an anonymised dataset will be maintained indefinitely at Birkbeck.

END OF THE TRIAL – This will be defined as database lock e.g. removal of user access of the REDCAP database.

Qualitative data analysis

Qualitative data will be analysed by a member of the research team using thematic analysis[29] to assess whether parents felt empowered by implementing the intervention, along with feasibility and barriers to adherence.

Statistical methods for primary and secondary outcomes {20a}

Quantitative data analysis will be performed by the trial statistician under the supervision of the senior trial statistician at King's College London, using a password-protected computer in a secure office. A statistical analysis plan will be prepared and finalised before outcome data is assessed. All analyses will be performed on an intention to treat basis. There will be no formal interim analysis for this study, and no subgroup analyses have been planned.

Descriptive statistics of study population

Trial flow data will be reported as outlined by the CONSORT diagram. The socioeconomic profiles of enrolled families will be compared to geographic norms to assess the success of probabilistic sampling. Baseline demographics, baseline and follow up outcome measures (e.g. Total toddler night-time sleep duration, single search saccadic reaction time from Visual Search Task), along with debrief questionnaire responses will be summarised using descriptive statistics. Continuous variables will be described using means and standard deviations (SDs), and categorical variables will be described using frequencies and proportions. The distribution of continuous variables will be checked, and if data is skewed, medians and quartiles will also be reported.

Primary feasibility outcomes

To assess the feasibility of a larger PASTI trial, descriptive statistical analyses will be used to assess participation rate, participant retention, and intervention adherence in addition to drafting the CONSORT participant flowchart as previously described (i.e.

number of families who are eligible, probabilistically sampled, randomised, etc). The following details will be reported:

1. Participation rate:
    - Number of consented participants
    - Number of eligible participants
    - Proportion of consented participants who are eligible to take part
    - Distribution of socioeconomic profiles for families enrolled relative to geographic norms (e.g. Central/Greater London)
2. Intervention adherence (in the PASTI Arm):
    - Average proportion of caregivers that reported no screen time in the hour before bed during the intervention period (week 1 – week 6; see table 1)
3. Participant retention:
    - Number and proportion of participants attending the follow-up lab visit out of those randomised overall and by trial arm
    - Number and proportion of participants that complete all follow-up outcome home and lab assessments out of those randomised overall and by trial arm
4. Participant experiences (in the PASTI Arm):
    - Distribution of parent experience ratings from the Debrief Survey and Debrief Interview completed by PASTI Arm participants
5. Assessment acceptability (in the PASTI Arm):
    - Distribution of study assessment acceptability ratings from the Debrief Survey and Debrief Interview completed by PASTI Arm participants

Secondary clinical feasibility outcomes

To obtain preliminary effect size estimates for PASTI on infant sleep, attention, and screen time measures at the follow-up timepoint, linear models will be used for each continuous outcome predicted by Intervention group (PASTI vs. No Intervention, PASTI vs. Bedtime Box), corresponding baseline measures of the outcome, and minimisation factors. Effect sizes and 95% confidence intervals (CIs) for the following outcomes will be presented:

1. Total night-time sleep duration, collected using triaxial accelerometer-based actigraphy
2. Exogenous attention control, a measure of single search saccadic reaction time from the Visual Search Task
3. Average toddler screen use (minutes) in the hour before bed, measured through an average of one weekday and one weekend bi-weekly bedtime activity diary directly preceding the lab visits.
4. Average nap duration using actigraphy
5. Frequency of night awakenings using actigraphy
6. Sleep efficiency using actigraphy
7. Parent-reported sleep onset latency from the BISQ-R
8. Prosaccade saccadic reaction time from the Antisaccade Task
9. Proportion of antisaccades in the Antisaccade Task
10. Baseline saccadic reaction time from the Gap-Overlap Task
11. Disengagement saccadic reaction time from the Gap-Overlap Task
12. Parent-reported effortful control subscale from the ECBQ
13. Parent-reported inhibitory control subscale from the ECBQ

Statistical analysis of clinical secondary outcomes

Modelling assumptions will be checked (e.g., normally distributed residuals). P-values will not be reported.

Populations under investigation

- The primary analysis population is defined as all randomised participants with a post-baseline follow-up assessment stated as the intention-to-treat approach (ITT) population.
- A per protocol population (PPP) will only include participants that follow the protocol. Participants that breach the protocol will be recoded as protocol violators (PV). A protocol violator will be defined as a breach of the protocol that may lead to a change in the study findings. Participants that breach the protocol in a manner that does not impact of the study findings will be recoded as protocol deviators, these will be included in the PPP.

A modified intention to treat (mITT) approach may be taken where analysis for secondary toddler sleep and attention control outcomes only includes participants that have completed the follow-up lab visit.

Handling of withdrawals, losses to follow-up, and missing data

Every effort will be made to reduce loss to follow-up and to collect outcome data from participants who have withdrawn should they give permission to do so.

The frequency and proportion of missing data at baseline and follow-up timepoints will be briefly described for the total sample and by trial arm. Completion rates for the daily adherence questionnaires in the PASTI Arm will be reported.

If published questionnaires do not have any guidelines on how to handle missing observations, calculating total pro-rata scores for subscales or scales may be considered if appropriate (e.g. if the participant has completed at least 80% of questionnaire items).

If there is missing baseline data for our linear models, an appropriate approach as recommended by literature will be taken (e.g. Mean imputation, or inverse probability weighting). Missing outcome data will not be imputed and an mITT approach will be taken as stated above.

Methods: Monitoring

Composition of the coordinating centre and trial steering committee {5d}

The Trial Management Group (TMG) comprises:

- Professor Tim Smith – Chief Investigator (CI)
- Dr Rachael Bedford – Co-Investigator
- Dr Ben Carter – Co-Investigator and Senior Trial Statistician
- Dr Hannah Pickard – Postdoctoral Research Associate (PDRA)
- Petrina Chu – Trial Statistician
- Claire Essex – Research Assistant (RA; December 2021 – December 2022)
- Emily Goddard – RA (January 2023 – Present)

The TMG have inputted into the protocol.

Trial Steering Committee (TSC):

- 1 Clinician (Chair)
- 1 Developmental Psychologist
- 1 Statistician
- 1 Parent Practitioner
- 1 expert by experience
- 1 Data Protection Officer

The TSC will meet before the beginning of the trial and every 6-12 months to examine data and adverse events.

An Early Years Stakeholder (EYS) Group have met periodically to co-create PASTI materials. This group was also contacted via email to provide feedback on the created materials prior to launching the trial. All members will be contacted again towards the end of the trial to discuss future directions.

Composition of the data monitoring committee, its role and reporting structure {21a}

An independent TSC has been established to examine the conduct of the trial; they will meet before the beginning of the trial and then every 6-12 months to oversee the trial. A trial report is prepared in advance. A charter for the group is available from the corresponding author. The TSC are acting in place of a data monitoring committee.

Interim analyses {21b}

No formal interim analysis is planned.

Adverse event reporting and harms {22}

Definitions of adverse events:

Adverse Event (AE): Any untoward medical occurrence in a participant to whom an intervention has been administered including occurrences which are not necessarily caused by or related to that intervention.

Adverse Reaction (AR): Any untoward and unintended response in a subject to an intervention which is related to any duration of intervention administered to that subject.

Unexpected Adverse Reaction (UAR): An adverse reaction the nature and severity of which is not consistent with the information known about the intervention in question in the view of the investigator.

Suspected Unexpected Serious Adverse Reactions (SUSAR): Any serious adverse event that is deemed to be related to the trial intervention and unexpected (not listed in the protocol as an expected side effect of the intervention).

In clinical trials for non-medicinal investigational products, a **serious adverse event (SAE)** is defined as an untoward occurrence that:

A) results in death;

B) is life-threatening

C) requires hospitalisation or prolongation of existing hospitalisation

D) results in persistent or significant disability or incapacity;

E) consists of a congenital anomaly or birth defect, or;

F) is otherwise considered medically significant by the investigator.

Upon randomisation, participants will be given verbal and written instructions of how to report adverse events throughout the trial. Participants in all arms will be able to report potential adverse events to the trial team using an online form embedded within the frequently asked questions support resource. Participants in the PASTI Arm and Bedtime Box Arm will also be able to report potential adverse events in the debrief online questionnaire at the end of the trial. The record will include a brief description of the event, any clinical symptoms, and a date when the event started and stopped, and who was involved (e.g. the infant, a parent, or other family member). Completed adverse event report forms will be reviewed by the research team to identify any SUSARs and request additional information from participants if needed. Adverse events will be monitored, recorded, and summarised. Should a serious adverse event be reported, then the intervention will be stopped for the relevant participant and a list of support services will be offered.

Serious adverse events that are related and unexpected (SUSARs) will be reported and closely monitored according to Birkbeck, University of London Research Ethics Committee reporting guidelines. The Trial Steering Committee will be made aware of all serious adverse reactions/SUSARs.

PASTI is not a pharmacological intervention, and so physical adverse reactions are not expected. The following list describes expected adverse events:

- Increased infant tantrums

- Infant sleep related-problems
- Changes to infant appetite
- Loss of interest in previously enjoyed activities
- Increased caregiver anxiety/stress
- Increased stress on household relationships

The assessment of whether or not an adverse event is related to study participation will be based on all available information at the time of the adverse event report completion. The following guidelines will be used to assess adverse event relationship:

- Definitely related: there is clear evidence suggesting a relationship and other factors are unlikely
- Probably related: there is evidence suggesting a relationship and other factors are unlikely
- Possibly related: there is some evidence suggesting a relationship but other factors may be possible
- Unlikely to be related: there is little evidence suggesting a relationship and other factors are more reasonable
- Unrelated: no evidence of any relationship

The following guidelines will be used to help assess adverse event severity:

- Mild: an event resulting in minimal or temporary discomfort, not interfering

with daily activities

- Moderate: an event resulting in sufficient discomfort that interferes with daily activities and that may require interventional treatment (e.g. aspirin for headaches)
- Severe: an event resulting in significant discomfort that prevents normal everyday activities

Frequency and plans for auditing trial conduct {23}

The trial steering committee will monitor the trial conduct. There are no formal plans for an audit, however, we will adhere to the King's Clinical Trials Unit Standard Operating Procedures.

Ethics and dissemination

Research ethics approval {24}

Ethical approval was given by Birkbeck, University of London Research Ethics Committee (Reference: 2122037)

Protocol amendments {25}

Any protocol amendments will be communicated to all involved parties by email. The Trial Protocol will be uploaded to the Open Science Framework and amendments will be tracked through uploading modified versions of the protocol.

Consent or assent {26a}

Electronic informed consent will be obtained from all potential trial participants (see Appendix G). Informed consent will be collected in two stages: 1) participants will consent to completing our pre-screen questionnaire; and 2) if eligible, participants will consent to take part in our trial. The unblinded researcher will also gather verbal informed consent from trial participants post randomisation.

Additional consent provisions for collection and use of participant data and biological specimens {26b}

Not applicable, as no biological specimens will be collected.

Confidentiality {27}

Participants research data (questionnaire and experimental data) will be pseudo-randomised using the participants unique ID number and stored in a password-protected database on the secure Birkbeck server, as described above. All video and audio data will be identified using the participant's unique ID number only. Participants personal identifying information (e.g. name, email address and contact number) will be kept in a separate password-protected master file in the separate location on the secure Birkbeck server. When participants are randomised into intervention arm, the randomisation software generates a unique pin for each participant. The pin will be used as the unique identifier for the pseudo-anonymised data entered into the research database (REDCap), and used by the trial statisticians for data analysis. The link between the participants unique ID number and unique pin number will be stored in a password-protected file on the secure

Birkbeck server. The unique pin will never be stored with any participant identifying information (e.g. name, email).

Declaration of interest {28}

We report no declarations of interest.

Access to data {29}

The datasets generated and/or analysed during the study will not be made publicly available. At the end of the trial necessary pseudo-anonymised data will be made publicly available via the Birkbeck Research Data repository.

Ancillary and post-trial care {30}

Families wishing to seek further support for their child post-trial will be signposted to appropriate services where required (e.g. GP).

Dissemination plans {31a}

1. Results of the study will be disseminated to participants via a study newsletter.
2. Findings from the study will be published in several peer reviewed papers. These will include journals targeted at academics and early years professionals (e.g. practitioners).

3. Study results will also be presented at academic and early years conferences. For example, British Psychological Society (BPS) Developmental conference and International Congress of Infant Studies (ICIS).
4. Findings from the study will be disseminated to the public and professionals through invited talks at charities and trusts (e.g. EYA, NCT, The Sleep Charity), as well as through blogs/podcasts via social media.
5. Study results will be published via a public report on the Nuffield Foundation website.

Author's contributions {31b}

We have no plans to employ professional medical writers.

Plans to give access to the full protocol, participant level-data and statistical code {31c}

The protocol will be published on the trials registry website. At the end of the trial all outcome data, trial information, and necessary pseudo-anonymised personal data (e.g. family demographic, child age, gender, etc) will be made publically available via the Birkbeck Research Data repository. Statistical code will be shared on a public data repository.

References

1. Chan AW, Tetzlaff JM, Altman DG, Laupacis A, Gøtzsche PC, Krleža-Jerić K, Hróbjartsson A, Mann H, Dickersin K, Berlin JA, Doré CJ, Parulekar WR, Summerskill WS, Groves T, Schulz KF, Sox HC, Rockhold FW, Rennie D, Moher D. SPIRIT 2013 statement: defining standard protocol items for clinical trials. *Annals of internal medicine.* 2013; 158(3), 200–207. 10.7326/0003-4819-158-3-201302050-00583
2. Ofcom. Children and parents: media use and attitudes report. Ofcom.org.uk. 2019. Accessed December 2021.
3. Cheung C, Bedford R, Saez De Urabain IR, Karmiloff Smith A, Smith TJ. Daily touchscreen use in infants and toddlers is associated with reduced sleep and delayed sleep onset. *Sci Rep.* 2017; 7, 46104. 10.1038/srep46104
4. Portugal AM, Bedford R, Cheung CH, Gliga T, Smith TJ. Saliency-driven visual search performance in toddlers with low–vs high–touch screen use. JAMA pediatrics. 2021;175(1):96-7. 10.1001/jamapediatrics.2020.2344
5. Portugal AM, Bedford R, Cheung CH, Mason L, Smith TJ. Longitudinal touchscreen use across early development is associated with faster exogenous and reduced endogenous attention control. Scientific Reports. 2021;11(1):2205. 10.1038/s41598-021-81775-7
6. Schmidt ME, Haines J, O'brien A, McDonald J, Price S, Sherry B, Taveras EM. Systematic review of effective strategies for reducing screen time among young children. *Obesity.* 2012;20(7):1338-54. 10.1038/oby.2011.348
7. American Academy of Pediatrics. Media and Young Minds. https://publications.aap.org/pediatrics/article/138/5/e20162591/60503/Media-

and-Young-Minds?autologincheck=redirected. November 2016. Accessed October 2023.

8. Carter B, Rees P, Hale L, Bhattacharjee D, Paradkar MS. Association between portable screen-based media device access or use and sleep outcomes: a systematic review and meta-analysis. *JAMA pediatrics.* 2016;170(12):1202-8. 10.1001/jamapediatrics.2016.2341
9. Mireku MO, Barker MM, Mutz J, Dumontheil I, Thomas MS, Röösli M, Elliott P, Toledano MB. Night-time screen-based media device use and adolescents' sleep and health-related quality of life. *Environment international.* 2019;124:66-78. 10.1016/j.envint.2018.11.069
10. Chang AM, Aeschbach D, Duffy JF, Czeisler CA. Evening use of light-emitting eReaders negatively affects sleep, circadian timing, and next-morning alertness. *Proceedings of the National Academy of Sciences.* 2015;112(4):1232-7. 10.1073/pnas.1418490112
11. Mindell JA, Sadeh A, Kohyama J, How TH. Parental behaviors and sleep outcomes in infants and toddlers: A cross-cultural comparison. *Sleep Medicine*. 2010; 11(4), 393–399. 10.1016/j.sleep.2009.11.011
12. Hoyniak CP, Petersen IT, McQuillan ME, Staples AD, Bates JE. Less Efficient Neural Processing Related to Irregular Sleep and Less Sustained Attention in Toddlers. *Dev Neuropsychol.* 2015;40(3):155-166. 10.1080/87565641.2015.1016162
13. Stein MA, Weiss M, Hlavaty L. ADHD treatments, sleep, and sleep problems: complex associations. *Neurotherapeutics.* 2012;9(3):509-517. 10.1007/s13311-012-0130-0

14. Hendry A, Jones EJ, Charman T. Executive function in the first three years of life: Precursors, predictors and patterns. *Developmental Review*. 2016;42:1-33. 10.1016/j.dr.2016.06.005
15. Lillard AS, Peterson J. The immediate impact of different types of television on young children's executive function. *Pediatrics.* 2011;128(4):644-9. 10.1542/peds.2010-1919
16. Nikkelen SW, Valkenburg PM, Huizinga M, Bushman BJ. Media use and ADHD-related behaviors in children and adolescents: A meta-analysis. *Developmental psychology*. 2014;50(9):2228. 10.1037/a0037318
17. Przybylski AK. Digital screen time and pediatric sleep: Evidence from a preregistered cohort study. *The Journal of Pediatrics.* 2019; *205,* 218-223.
18. Dennison BA, Russo TJ, Burdick PA, Jenkins PL. An intervention to reduce television viewing by preschool children. *Archives of pediatrics & adolescent medicine*. 2004;158(2):170-6. 10.1001/archpedi.158.2.170
19. Sadeh A. A brief screening questionnaire for infant sleep problems: validation and findings for an Internet sample. *Pediatrics.* 2004;113(6):e570-7. 10.1542/peds.113.6.e570
20. Marteau TM, Bekker H. The development of a six-item short-form of the state scale of the Spielberger State–Trait Anxiety Inventory (STAI). *British Journal of Clinical Psychology*. 1992; 31(3), 301–306. 10.1111/j.2044-8260.1992.tb00997.x
21. Arain M, Campbell MJ, Cooper CL, Lancaster GA. What is a pilot or feasibility study? A review of current practice and editorial policy. *BMC Medical Research Methodology.* 2010; 10(1), 67. 10.1186/1471-2288-10-67

22. Teare MD, Dimairo M, Shephard N, Hayman A, Whitehead A, Walters SJ. Sample size requirements to estimate key design parameters from external pilot randomized controlled trials: a simulation study. *Trials.* 2014;15:1-3. 10.1186/1745-6215-15-264

23. Putnam SP, Gartstein MA, Rothbart MK. Measurement of fine-grained aspects of toddler temperament: The Early Childhood Behavior Questionnaire. *Infant behavior and development.* 2006;29(3):386-401. 10.1016/j.infbeh.2006.01.004

24. Sparrow S, Cicchetti DV. Saulnier CA. Vineland Adaptive Behavior Scales, Third Edition (Vineland-3). San Antonio, TX: Pearson. 2016.

25. Spielberger CD, Gorsuch RL, Lushene R, Vagg R, Jacobs GS. Manual for the Stait-Trait Anxiety Inventory. Palo Alto, CA: Consulting Psychologists Press; 1983.

26. Sadeh A, Hauri PJ, Kripke DF, Lavie P. The role of actigraphy in the evaluation of sleep disorders. *Sleep.* 1995; 18(4), 288–302. 10.1093/sleep/18.4.288

27. Kaldy Z, Kraper C, Carter AS, Blaser E. Toddlers with autism spectrum disorder are more successful at visual search than typically developing toddlers. *Developmental science*. 2011(5):980-8. 10.1111/j.1467-7687.2011.01053.x

28. Mullen EM. Mullen scales of early learning. Circle Pines, MN: AGS; 1995.

29. Clarke V, Braun V. Thematic analysis. In Encyclopedia of critical psychology. Springer, New York, NY; 2014.

Appendices

Appendix A – Caregiver Screen Time Questionnaire

Please answer the following questions about your **child's use of screen time in the hour before bed today.**	
How long did your child have any screen time for in the hour before bed today (e.g., watching, playing-with, or looking-at a screen including TV, tablets, phones, laptops, etc.)?<br><br>The screen could be on for them or someone else in the room.	0-60 minutes
Which screen time device(s) did your child view in the hour before bed today?<br><br>Please tick all that apply.	□ TV – played for them<br>□ Background TV – played for somebody else<br>□ Tablet/iPad<br>□ Smartphone<br>□ Computer/laptop<br>□ Other________________
What did your child have screen time for in the hour before bed today?	□ Passively watch tv/videos<br>□ Scroll or touch the screen<br>□ Call someone (e.g. phone call)<br>□ Video call someone (e.g. Zoom, FaceTime)<br>□ Play games<br>□ Use educational apps<br>□ Use art/creative apps such as drawing<br>□ Listen to music<br>□ Look at photos<br>□ Take photos<br>□ Web search (e.g. Google)<br>□ Text message (e.g. WhatsApp)<br>□ Social media (e.g. Instagram)<br>□ Other:_____________________

Appendix B – Bedtime Activity Diary

Weekday diary

Please answer the following questions about your **child's hour before bed today.**<br><br>*The hour before bed means the hour before you put your child down to sleep. For example, if you put your child down to sleep at 8pm, then please think about the time between 7pm and 8pm.*	
What time did you put your child down to sleep today?<br><br>HH:MM (e.g. 19:00)	HH:MM
**Play**	
How long did your child play for in the hour before bed today (e.g., crafts, colouring, puzzles, toys)?	0-60 minutes
Which play activities did your child do in the hour before bed today?	□ Crafts (e.g. making a picture)<br>□ Play with toys<br>□ Do a puzzle<br>□ Play a game with family member<br>□ Physical play<br>□ Construction (e.g. building blocks)
**Screen time**	
How long did your child have any screen time for in the hour before bed today (e.g., watching, playing-with, or looking-at a screen including TV, tablets, phones, laptops, etc.)?<br><br>The screen could be on for them or someone else in the room.	0-60 minutes
Which screen time device(s) did your child view in the hour before bed today?<br><br>Please tick all that apply.	□ TV – played for them<br>□ Background TV – played for somebody else<br>□ Tablet/iPad<br>□ Smartphone<br>□ Computer/laptop<br>□ Other________________
What did your child have screen time for in the hour before bed today?	□ Passively watch tv/videos<br>□ Scroll or touch the screen<br>□ Call someone (e.g. phone call)

	☐ Video call someone (e.g. Zoom, FaceTime)<br>☐ Play games<br>☐ Use educational apps<br>☐ Use art/creative apps such as drawing<br>☐ Listen to music<br>☐ Look at photos<br>☐ Take photos<br>☐ Web search (e.g. Google)<br>☐ Text message (e.g. WhatsApp)<br>☐ Social media (e.g. Instagram)<br>☐ Other:____________________
**Eat**	
How long did your child spend <u>eating</u> in the hour before bed today (e.g. main evening meal, bedtime snack, etc.)?	0-60 minutes
**Read books**	
How long did you and your child <u>read books</u> for in the hour before bed today?	0-60 minutes
**Wind down activities**	
How long did you do <u>wind down activities</u> with your child in the hour before bed today (e.g. listening to music, singing, rocking, massage, etc.)?	0-60 minutes
How long did you spend getting your child ready for bed today (e.g. having a bath, brushing teeth, putting on pyjamas, etc.)?	0-60 minutes
How long did it take your child to fall asleep?	_________ minutes
Between putting your child down to sleep and them falling asleep, which activities did your child do?	☐ Went straight to sleep<br>☐ Was massaged<br>☐ Was rocked or cuddled<br>☐ Had a snack<br>☐ Had a bottle, drink, or being breastfeed<br>☐ Physical play (e.g. running, jumping, bouncing)<br>☐ Played with toys<br>☐ Read books/was read to<br>☐ Had screen time (e.g. watch or play with a tablet, phone, or TV)<br>☐ Said prayers

	☐ Sung songs<br>☐ Listened to music<br>☐ Laid in bed quietly<br>☐ Other _________
Please answer the following questions about **yourself.**	
A number of statements which people have used to describe themselves are given below. Read each statement and then chose an answer to indicate how you feel right now, that is, at this moment. There are no right or wrong answers. Do not spend too much time on any one statement but give the answer which seems to describe your present feeling best.	
I feel calm	Not at all, somewhat, moderately so, very much so
I am tense	Not at all, somewhat, moderately so, very much so
I feel upset	Not at all, somewhat, moderately so, very much so
I am relaxed	Not at all, somewhat, moderately so, very much so
I feel content	Not at all, somewhat, moderately so, very much so
I am worried	Not at all, somewhat, moderately so, very much so

Weekend diary

Please answer the following questions about your **child's hour before bed today.**<br><br>*The hour before bed means the hour before you put your child down to sleep. For example, if you put your child down to sleep at 8pm, then please think about the time between 7pm and 8pm.*	
What time did you put your child down to sleep today?<br><br>HH:MM (e.g. 19:00)	HH:MM
**Play**	
How long did your child play for in the hour before bed today (e.g., crafts, colouring, puzzles, toys)?	0-60 minutes
Which play activities did your child do in the hour before bed today?	☐ Crafts (e.g. making a picture)<br>☐ Play with toys<br>☐ Do a puzzle<br>☐ Play a game with family member<br>☐ Physical play<br>☐ Construction (e.g. building blocks)

**Screen time**	
How long did your child <u>have any screen time</u> for in the hour before bed today (e.g., watching, playing-with, or looking-at a screen including TV, tablets, phones, laptops, etc.)?<br><br>The screen could be on for them or someone else in the room.	0-60 minutes
Which screen time device(s) did your child view in the hour before bed today?<br><br>Please tick all that apply.	□ TV – played for them<br>□ Background TV – played for somebody else<br>□ Tablet/iPad<br>□ Smartphone<br>□ Computer/laptop<br>□ Other________________
What did your child have screen time for in the hour before bed today?	□ Passively watch tv/videos<br>□ Scroll or touch the screen<br>□ Call someone (e.g. phone call)<br>□ Video call someone (e.g. Zoom, FaceTime)<br>□ Play games<br>□ Use educational apps<br>□ Use art/creative apps such as drawing<br>□ Listen to music<br>□ Look at photos<br>□ Take photos<br>□ Web search (e.g. Google)<br>□ Text message (e.g. WhatsApp)<br>□ Social media (e.g. Instagram)<br>□ Other:_____________________
**Eat**	
How long did your child spend <u>eating</u> in the hour before bed today (e.g. main evening meal, bedtime snack, etc.)?	0-60 minutes
**Read books**	
How long did you and your child <u>read books</u> for in the hour before bed today?	0-60 minutes
**Wind down activities**	
How long did you do <u>wind down activities</u> with your child in the hour before bed today (e.g. listening to music,	0-60 minutes

singing, rocking, massage, etc.)?	
How long did you spend getting your child ready for bed today (e.g. having a bath, brushing teeth, putting on pyjamas, etc.)?	0-60 minutes
How long did it take your child to fall asleep?	__________ minutes
Between putting your child down to sleep and them falling asleep, which activities did your child do?	□ Went straight to sleep<br>□ Was massaged<br>□ Was rocked or cuddled<br>□ Had a snack<br>□ Had a bottle, drink, or being breastfeed<br>□ Physical play (e.g. running, jumping, bouncing)<br>□ Played with toys<br>□ Read books/was read to<br>□ Had screen time (e.g. watch or play with a tablet, phone, or TV)<br>□ Said prayers<br>□ Sung songs<br>□ Listened to music<br>□ Laid in bed quietly<br>□ Other __________
Please think about your **<u>child's sleep during the past week</u>** when answering the following questions.	
On a typical day in the past week, how much total time did your child spend sleeping during the DAY (between when your child wakes for the day and goes to bed at night)?<br>Example: If your child took 2 naps and slept 1 hour each time, your child's total time spent sleeping during the day is 2 hours.	Hours and minutes
On a typical day in the past week, how long did it usually take your child to fall asleep?<br><br>Example: If you put your child to bed at 6:30 pm and your child falls asleep at 8:00 pm, it	Hours and minutes

takes 1 hour and 30 minutes for your child to fall asleep.	
On a typical day in the past week, how many times did your child usually wake during the night?	__________times per night
On a typical day in the past week, how much total time did your child spend sleeping during the NIGHT (between when your child goes to bed and wakes for the day)?<br><br>Example: If your child sleeps for 3 hours, wakes up, then sleeps for 5 hours and 30 minutes more, your child sleeps for 8 hours and 30 minutes total.	Hours and minutes

Appendix C – Example Consort Diagram

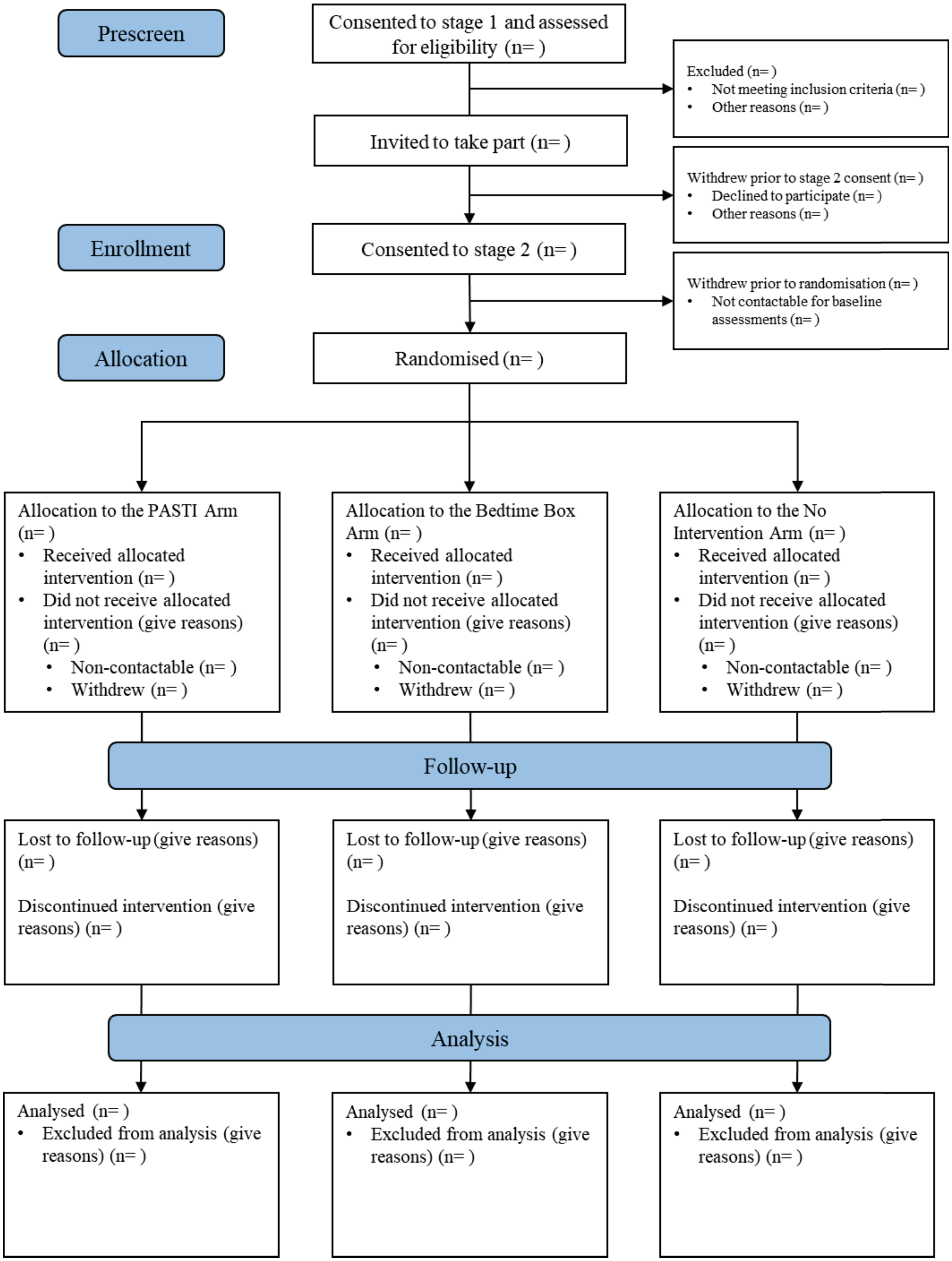

Appendix D – Pre-screen Questionnaire

Please answer the following questions about **your child.**	
What is your child's sex?	□ Male<br>□ Female<br>□ Other:__________
What is your child's date of birth?	DD/MM/YYYY
How old is your child in months?	12-30 months
What is your child's ethnicity?<br><br>[Categories taken from the Office of National Statistics]	**White**<br>English/Welsh/Scottish/Northern Irish/British<br>Irish<br>Gypsy or Irish Traveler<br>Any other White background, please describe<br><br>**Mixed/Multiple ethnic groups**<br>White and Black Caribbean<br>White and Black African<br>White and Asian<br>Any other Mixed/Multiple ethnic background, please describe<br><br>**Asian/Asian British**<br>Indian<br>Pakistani<br>Bangladeshi<br>Chinese<br>Any other Asian background, please describe<br><br>**Black/ African/Caribbean/Black British**<br>African<br>Caribbean<br>Any other Black/African/Caribbean background, please describe<br><br>**Other ethnic group**<br>Arab<br>Any other ethnic group, please describe
Does your child have any medical conditions?	□ Yes<br>□ No
If yes, please tell us about the medical conditions your child has?	_________________

How many days a week is your child in nursery/childcare?<br><br>Please count each partial day as 1.	0-7
How many younger siblings does your child have?	__________
How many older siblings does your child have?	__________
Who is filling out this questionnaire?	□ Mother<br>□ Father<br>□ Other caregiver:______________
Please answer the following questions about **<u>yourself.</u>**	
What gender do you most identify with?	□ Male<br>□ Female<br>□ I prefer to self-describe as: ______________
What is your age (in years)?	________ years old
What is your ethnicity?<br><br>[Categories taken from the Office of National Statistics]	**White**<br>English/Welsh/Scottish/Northern Irish/British<br>Irish<br>Gypsy or Irish Traveler<br>Any other White background, please describe<br><br>**Mixed/Multiple ethnic groups**<br>White and Black Caribbean<br>White and Black African<br>White and Asian<br>Any other Mixed/Multiple ethnic background, please describe<br><br>**Asian/Asian British**<br>Indian<br>Pakistani<br>Bangladeshi<br>Chinese<br>Any other Asian background, please describe<br><br>**Black/ African/Caribbean/Black British**<br>African<br>Caribbean<br>Any other Black/African/Caribbean background, please describe<br><br>**Other ethnic group**<br>Arab

	Any other ethnic group, please describe
What is your highest level of education?	□ None<br>□ School leaving qualification or equivalent<br>□ College or equivalent<br>□ University or equivalent<br>□ Post-graduate or equivalent<br>□ Not applicable, please specify____________
Do you speak fluent English?	□ Yes<br>□ No, please describe________________
Do you live in Greater/Central London?	□ Yes<br>□ No, please describe________________
Are you the only parent/caregiver?	□ Yes<br>□ No
If no: Please answer the following questions about **parent/caregiver #2**	
Which gender does parent/caregiver #2 most identify with?	□ Male<br>□ Female<br>□ They prefer to self-describe as: _____________
What is parent/caregiver #2 age (in years)?	_______ years old
Please answer the following questions about **your child's birth.**	
Please tell us the number of completed weeks of pregnancy when your child was born (i.e. full term = 40 weeks)	23 - 44 weeks
Please answer the following questions about your **child's hour before bed.** Please think about a typical week for you.<br><br>*The hour before bed means the hour before you put your child down to sleep. For example, if you put your child down to sleep at 8pm, then please think about the time between 7pm and 8pm.*	
**Play**	
In a typical week, how many days does your child play in the hour before bed (e.g., crafts, colouring, puzzles, toys)?	0-7

On these days, how long does your child typically play for in the hour before bed?	0-60
**Screen time**	
In a typical week, how many days does your child have any screen time in the hour before bed (e.g., watching, playing-with, or looking-at a screen including TV, tablets, phones, laptops, etc)?<br><br>The screen could be on for them or someone else in the room.	0-7
On these days, how long does your child have any screen time for in the hour before bed (e.g., watching, playing-with, or looking-at a screen including TV, tablets, phones, laptops, etc)?<br><br>The screen could be on for them or someone else in the room.	0-60
**Eat**	
In a typical week, how many days does your child eat in the hour before bed (e.g. main evening meal, bedtime snack, etc)?	0-7
On these days, how long does your child spend eating in the hour before bed (e.g. main evening meal, bedtime snack, etc)?	0-60
**Read books**	
In a typical week, how many days do you and your child read books in the hour before bed?	0-7

On these days, how long do you and your child read books for in the hour before bed?	0-60
**Wind down activities**	
In a typical week, how many days do you do wind down activities with your child in the hour before bed (e.g. listening to music, singing, rocking, massage, etc)?	0-7
On these days, how long do you do wind down activities for in the hour before bed (e.g. listening to music, singing, rocking, massage, etc)?	0-60
On a typical day, how long do you spend getting your child ready for bed (e.g. having a bath, brushing teeth, putting on pyjamas, etc)?	0-60
Who usually puts your child to bed?<br><br>Please tick all that apply.	□ Mother<br>□ Father<br>□ Other caregiver:________________
Please think about your **child's sleep during the past two weeks** when answering the following questions.	
How much total time does your child spend sleeping during the DAY (between when your child wakes for the day and goes to bed at night)?<br><br>Example: If your child took 2 naps and slept 1 hour each time, your child's total time spent sleeping during the day is 2 hours.	Hours and minutes
How long does it usually take your child to fall asleep?	Hours and minutes

Example: If you put your child to bed at 6:30 pm and your child falls asleep at 8:00 pm, it takes 1 hour and 30 minutes for your child to fall asleep.	
How many times does your child usually wake during the night?	__________times per night
How much total time does your child spend sleeping during the NIGHT (between when your child goes to bed and wakes for the day)?<br><br>Example: If your child sleeps for 3 hours, wakes up, then sleeps for 5 hours and 30 minutes more, your child sleeps for 8 hours and 30 minutes total.	Hours and minutes
Is your child currently taking part in another trial/research study?	□ Yes<br>□ No
Where did you hear about this study?<br><br>Please tick all that apply.	□ Early Years Alliance (EYA)<br>□ National Childbirth Trust (NCT)<br>□ The Sleep Charity<br>□ Nursery/daycare<br>□ Child Centre<br>□ Social media (Facebook, Twitter, Instagram)<br>□ Centre for Brain and Cognitive Development (CBCD) Babylab Database<br>□ Word of mouth<br>□ Other

Appendix E – Daytime Activity Levels Questionnaire

We will now ask some questions about **<u>your child's general daytime activity levels</u>** in the **<u>past week</u>.**	
In the past week, how many days was your child in nursery/childcare?<br><br>Please count each partial day as 1.	_______ days
On a typical weekday in the past week, how long did your child spend doing active play (e.g., running, dancing)?	Hours and minutes
On a typical weekend day in the past week, how long did your child spend doing active play (e.g., running, dancing)?	Hours and minutes
On a typical weekday in the past week, how long did your child spend doing quiet play (e.g., puzzles, being read to)?	Hours and minutes
On a typical weekend day in the past week, how long did your child spend doing quiet play (e.g., puzzles, being read to)?	Hours and minutes
On a typical weekday in the past week, how long did your child have any screen time for (e.g., watching, playing-with, or looking-at a screen including TV, tablets, phones, laptops, etc.)?<br><br>The screen could be on for them or someone else in the room.	Hours and minutes
On a typical weekend day in the past week, how long did your child have any screen time for (e.g., watching, playing-with, or looking-at a screen including TV, tablets, phones, laptops, etc.)?	Hours and minutes

The screen could be on for them or someone else in the room.	

Appendix F – Sleep and Motion Watch Diary

Birkbeck UNIVERSITY OF LONDON

Sleep and Motion Watch Diary

*Use 24hr time	Example	Thursday	Friday	Saturday	Sunday	Monday	Tuesday	Wednesday	Thursday*	Friday*	Saturday*
Time child woke up	07:00										
Time and length of daytime naps	12.30, 60 minutes										
Where did your child nap?	In the car										
Time child put down to sleep	19:00										
Time child fell asleep	19:30										
Time and length of night awakenings	23:30, 30 minutes<br>03:50, 20 minutes										
Where did your child sleep at night?	In their own bed										
Time and length the motion watch was removed during the day/night	Not removed										
Was this a typical day for your child?	Yes										
Was this a typical night for your child?	No, they became unwell										

*You can stop completing the diary on the day of your Babylab visit. Please remember to bring your diary and the Motion Watch with you to your Babylab visit.

Appendix G – Informed consent materials {32}

<u>Consent Stage 1</u>

Please check the box below to confirm that you have read and understood the information above and have had the opportunity to ask any questions (by emailing bb-trial@bbk.ac.uk). By checking this box you agree to share your contact details and to be contacted if you are selected to take part in the Bedtime Boost Trial.

□ I agree to take part in stage 1 of this study

Contact details

Caregiver Name (participant):……………………………….

Date:…………………………………………………………….

Email address:…………………………………………………

Contact number (mobile):…………………………………….

Postcode:……………………………………………………….

Please create a unique ID number.

To create your unique ID number please use the following information: Your initials (e.g. MD), the last two digits of your year of birth (e.g. 87) and the last two digits of your phone number (e.g. 58), for example MD8758.

………………………………………………………………

Consent Stage 2

The Bedtime Boost Trial

Please read each statement below carefully.

- I have read the details of the study and willingly consent to my child and I taking part. Any questions I've asked have been answered to my satisfaction and I understand that I may ask further questions at any time (by emailing bb-trial@bbk.ac.uk).
- I understand that the information my child and I give will be used for this study and future follow up studies.
- I understand that I am able to withdraw myself and my child from this study at any time without giving a reason and can decline to answer particular questions. I understand that I will be able to withdraw mine/my child's data up until the data has been committed to being published e.g. 1st November 2023.
- I understand that audio/video recordings will be made during the study. These recordings will be identifiable by my unique participant ID and will only be used for the research project.
- I understand that all information given will be kept confidential. All data and videos will be stored securely within the Babylab facilities. To maintain confidentiality mine/my child's data will be given a unique ID number rather than a name for the researchers to identify it. Any link between my/my child's personal information (e.g. name) and unique ID number will be kept in Birkbeck's facilities for 10 years, but I can request to have it removed at any time.
- My involvement in the study will remain confidential unless I share information with the researcher which raises a serious concern about a child protection issue.
- I understand that the results of this project will be used for scientific publications and shared with the public and professionals (e.g. through talks and newsletters). Results will be presented at a group level, not at the individual level. If any individual data are presented, the data will be completely pseudo-anonymous, without any way of identifying the individuals involved.
- I understand that my final pseudo-anonymised dataset (including my pseudo-anonymised data) will be shared with members of the research team who are outside of Birkbeck, including King's College Trials Unit (KCTU), University of Bath, and Flow Associates. The data will never be used for commercial purposes.
- I understand that the research team may upload the final pseudo-anonymised dataset to a data repository.
- I confirm that I am over 16 years of age
- I'd like to be contacted for project updates (optional) – Yes/No
- I'd like to be contacted for future studies e.g. follow up studies to this project, or future studies of a similar nature (optional) – Yes/No

Caregiver Name (participant):……………………………….

Date:…………………………………………………………………….

Please tell us your home address.

Address line 1:…………………………………………………………..

Address line 2:…………………………………………………………..

Town/City:……………………………………………………………….

County (if applicable):……………………………………………..

Postcode:…………………………………………………………………..

Appendix H – Debrief Materials

Screen time arm

Debrief Sheet

Dear Caregiver,

Thank you for taking part in "The Bedtime Boost Study". In this study you may have been asked to change some of the activities your child does in the hour before bed. The overall aim of this study was to test the possibility of carrying out a **Parent-Administered Screen Time intervention** with children. This is the first trial of its kind with children, and we hope it will pave the way for more research looking at the impact of screen time on infant sleep and attention.

<u>Additional Information we could not share with you at the start of the study:</u>

Your child was selected for the study because

- They were 17-30 months old
- You live in London or surrounding areas
- They have no known neurological or genetic conditions
- They were born full term (37 weeks+)
- They were not currently involved in another research study or trial

Your child was also selected for this study because ***you reported that they used 10+ minutes of screen time in the hour before bed at least 3 days per week***. After you and your child were selected, you were randomly assigned to one of three groups.

The three groups were:

- ***Screen Time group.*** Families were instructed to help their child avoid all screen time in the hour before bed. They were given a 'family bedtime box' which contained some calming activities and toys to help them avoid screen time in the hour before bed.
- ***Bedtime Box group.*** Families were provided with a 'family bedtime box' which contained some calming activities and toys to be used in the hour before bed.
- ***Control group.*** Families were not given any materials. They were asked to continue their child's normal routine in the hour before bed.

For all families the trial period lasted for 7 weeks.

<u>Your child was randomly assigned to the ***Screen Time group.***</u>

<u>What did we expect to find?</u>
We expect that replacing screen time with other calming bedtime activities in the hour before bed should help improve children's sleep and attention.

We would like to thank you again for your family's contribution to this study, we really appreciate the time you have given to take part in this project. We believe this trial marks a significant move forward in the investigation of causal links between children's screen time and their sleep and attention. We look forward to being able to share our results with you in the future.

If you asked to be contacted with information on the findings of the study, we will be in touch again once the trial is complete and we have results to share.

Would you like to be involved in further research?
If you would like to be involved in future studies at the Babylab please visit our website (https://cbcd.bbk.ac.uk/babylab) to register.
If you have any questions/concerns about the study or the information provided in this debrief form, please do not hesitate to contact us. Contact details are provided below.

If you have any questions, please email our research team at bb-trial@bbk.ac.uk

If you have concerns about this study that have not been answered by the chief investigators, please contact the School's Ethics Officer at: ethics@psychology.bbk.ac.uk

School Research Officer

School of Science, Department of Psychological Sciences,

Birkbeck,

University of London,

London

WC1E 7HX

You also have the right to submit a complaint to the Information Commissioner's Office https://ico.org.uk/

You can withdraw your consent at any point without giving a reason. If you would like to withdraw, please contact the research team.

Bedtime Box Arm

Debrief Sheet

Dear Caregiver,

Thank you for taking part in "The Bedtime Boost Study". In this study you may have been asked to change some of the activities your child does in the hour before bed. The overall aim of this study was to test the possibility of carrying out a **Parent-Administered Screen Time intervention** with children. This is the first trial of its kind with children, and we hope it will pave the way for more research looking at the impact of screen time on infant sleep and attention.

Additional Information we could not share with you at the start of the study:

Your child was selected for the study because

- They were 17-30 months old
- You live in London or surrounding areas
- They have no known neurological or genetic conditions
- They were born full term (37 weeks+)
- They were not currently involved in another research study or trial

Your child was also selected for this study because ***you reported that they used 10+ minutes of screen time in the hour before bed at least 3 days per week***. After you and your child were selected, you were randomly assigned to one of three groups.

The three groups were:

- ***Screen Time group.*** Families were instructed to help their child avoid all screen time in the hour before bed. They were given a 'family bedtime box' which contained some calming activities and toys to help them avoid screen time in the hour before bed.
- ***Bedtime Box group.*** Families were provided with a 'family bedtime box' which contained some calming activities and toys to be used in the hour before bed.
- ***Control group.*** Families were not given any materials. They were asked to continue their child's normal routine in the hour before bed.

For all families the trial period lasted for 7 weeks.

Your child was randomly assigned to the ***Bedtime Box group.***

What did we expect to find?

We expect that replacing screen time with other calming bedtime activities in the hour before bed should help improve children's sleep and attention.

We would like to thank you again for your family's contribution to this study, we really appreciate the time you have given to take part in this project. We believe this trial marks a significant move forward in the investigation of causal links between children's screen time and their sleep and attention. We look forward to being able to share our results with you in the future.

If you asked to be contacted with information on the findings of the study, we will be in touch again once the trial is complete and we have results to share.

Would you like to be involved in further research?
If you would like to be involved in future studies at the Babylab please visit our website (https://cbcd.bbk.ac.uk/babylab) to register.
If you have any concerns about the study or the information provided in this debrief form, please do not hesitate to contact us. Contact details are provided below.

If you have any questions, please email our research team at bb-trial@bbk.ac.uk

If you have concerns about this study that have not been answered by the chief investigators, please contact the School's Ethics Officer at: ethics@psychology.bbk.ac.uk

School Research Officer

School of Science, Department of Psychological Sciences,

Birkbeck,

University of London,

London

WC1E 7HX

You also have the right to submit a complaint to the Information Commissioner's Office https://ico.org.uk/

You can withdraw your consent at any point without giving a reason. If you would like to withdraw, please contact the research team.

No intervention arm

Debrief Sheet

Dear Caregiver,

Thank you for taking part in "The Bedtime Boost Study". In this study you may have been asked to change some of the activities your child does in the hour before bed. The overall aim of this study was to test the possibility of carrying out a **Parent-Administered Screen Time intervention** with children. This is the first trial of its kind with children, and we hope it will pave the way for more research looking at the impact of screen time on infant sleep and attention.

Additional Information we could not share with you at the start of the study:

Your child was selected for the study because

- They were 17-30 months old
- You live in London or surrounding areas
- They have no known neurological or genetic conditions
- They were born full term (37 weeks+)
- They were not currently involved in another research study or trial

Your child was also selected for this study because ***you reported that they used 10+ minutes of screen time in the hour before bed at least 3 days per week***. After you and your child were selected, you were randomly assigned to one of three groups.

The three groups were:

- ***Screen Time group.*** Families were instructed to help their child avoid all screen time in the hour before bed. They were given a 'family bedtime box' which contained some calming activities and toys to help them avoid screen time in the hour before bed.
- ***Bedtime Box group.*** Families were provided with a 'family bedtime box' which contained some calming activities and toys to be used in the hour before bed.
- ***Control group.*** Families were not given any materials. They were asked to continue their child's normal routine in the hour before bed.

For all families the trial period lasted for 7 weeks.

Your child was randomly assigned to the ***Control group.***

You will be given the option of receiving the Family Bedtime Box. There is no obligation to take the box.

What did we expect to find?

We expect that replacing screen time with other calming bedtime activities in the hour before bed should help improve children's sleep/attention.

We would like to thank you again for your family's contribution to this study, we really appreciate the time you have given to take part in this project. We believe this trial marks a significant move forward in the investigation of causal links between children's screen time and their sleep and attention. We look forward to being able to share our results with you in the future.

If you asked to be contacted with information on the findings of the study, we will be in touch again once the trial is complete and we have results to share.

Would you like to be involved in further research?
If you would like to be involved in future studies at the Babylab please visit our website (https://cbcd.bbk.ac.uk/babylab) to register.

If you have any concerns about the study or the information provided in this debrief form, please do not hesitate to contact us. Contact details are provided below.

If you have any questions, please email our research team at bb-trial@bbk.ac.uk

If you have any concerns about the study or the information provided in this debrief form that have not been answered by the chief investigators, please contact the School's Ethics Officer at: ethics@psychology.bbk.ac.uk

School Research Officer

School of Science, Department of Psychological Sciences,

Birkbeck,

University of London,

London

WC1E 7HX

You also have the right to submit a complaint to the Information Commissioner's Office https://ico.org.uk/

You can withdraw your consent at any point without giving a reason. If you would like to withdraw, please contact the research team.

Biological specimens {33}

Not applicable
