# Supplementary material for: Toddler Screen Use Before Bed and Its Effect on Sleep and Attention: A Randomized Clinical Trial
Source: JAMA Pediatr. 2024 Oct 21;178(12):1270–9. doi: 10.1001/jamapediatrics.2024.3997 (PMC11581737; doi:10.1001/jamapediatrics.2024.3997)
Supplement: Supplement 2. — Statistical Analysis Plan. [file jamapediatr-e243997-s002.pdf]

Parent-Administered Screen Time Intervention (PASTI): A 7-week three arm assessor blinded Feasibility and Pilot Randomised Controlled Trial, compared to Bedtime-Box Intervention and No Intervention (1:1:1) in toddlers

Statistical Analysis Plan

Version 1.0

31 May 2023

ISRCTN58249751

Junior Trial Statistician: Petrina Chu

Signed: Date: 01 / 06 / 2023

Senior Trial Statistician: Dr Ben Carter

Signed: Date: 01 / June / 2023

Independent TSC Statistician: Joana Vasconcelos

Signed: Joana Vasconcelos Date: 01/06/2023

Chief Investigator: Prof Tim Smith

Signed: Date: 01/06/2023

Table of Contents

1. Description of the trial 5
1.1 Principal research objectives to be addressed 5
1.1.1 Primary objectives 5
1.1.2 Secondary objectives 5
1.2 Trial design 6
1.2.1 Participant enrolment 6
1.2.2 Post-randomisation 6
1.3 Eligibility criteria 7
1.4 Randomisation and blinding 7
1.4.1 Randomisation 7
1.4.2 Blinding and planned unblinding 8
1.5 Description of intervention period and frequency of follow-up 8
1.6 Visit windows 8
1.7 Data collection 8
1.8 Sample size estimation (including clinical significance) 9
1.9 Primary feasibility and secondary outcome measures 9
1.9.1. Primary feasibility outcome measures: 9
1.9.2 Secondary outcomes: 10
2. Quantitative Analysis Plan 11
2.1 Overview 11
2.2 Recruitment and representativeness of recruited patients 11
Figure 1. Template CONSORT diagram for PASTI feasibility trial 12
2.3 Description of sample characteristics at baseline 13
2.4 Estimation of feasibility parameters 13
2.5 Description of follow-up outcome measures 15
2.6 Secondary outcomes 15
2.6.1 Descriptive analysis 15
2.6.2 Treatment Effects 15
2.6.3 Estimands 15
2.6.4 Primary comparisons of between-group differences 16
2.7 Populations under investigation 16
2.8 Loss to follow-up and other missing data 16
2.8.1 Missing demographic and questionnaire data 16
2.8.2 Missing items in scales and subscales 16
2.8.3 Missing items in lab and actigraphy measures 17
2.8.4 Withdrawals and losses to follow-up 17
2.9 Pre-processing of actigraphy and eye-tracking data 17
2.9.1 Actigraphy data 17
2.9.2 Eye-tracking data 18
2.10 Adverse event reporting 19
2.11 Sensitivity and other analyses 20
2.11.1 Interim and subgroup analyses 20
2.12 Protocol adherence 20
2.12.1 Protocol violators 20
2.12.2 Protocol deviators 20
2.13 Data monitoring 21
2.14 Data checking 21
2.15 Software 21
2.16 Quality Control and assurance 22

Reference list **23**
Appendix 1: Description of study measures **24**
Pre-screening demographic measures 24
Baseline and follow-up home assessment measures 24
Baseline and follow-up lab assessment measures 25
Additional measures 25

Chief Investigator
Prof Tim Smith
Professor of Cognitive Psychology
Centre for Brain and Cognitive Development
Department of Psychological Sciences
Birkbeck, University of London

Co-Investigators
Dr Rachael Bedford
Senior Lecturer/Associate Professor
Psychology Department
University of Bath

Dr Ben Carter
Reader in Medical Statistics
Institute of Psychiatry, Psychology & Neuroscience
Department of Biostatistics and Health Informatics
King's College London

Dr Hannah Pickard
Postdoctoral Researcher
Centre for Brain and Cognitive Development
Department of Psychological Sciences
Birkbeck, University of London

Claire Essex
PhD Student/Research Assistant
Centre for Brain and Cognitive Development
Department of Psychological Sciences
Birkbeck, University of London

Emily Goddard
Research Assistant
Centre for Brain and Cognitive Development
Department of Psychological Sciences
Birkbeck, University of London

Trial statistician
Petrina Chu
Institute of Psychiatry, Psychology & Neuroscience
Department of Biostatistics and Health Informatics
King's College London

1. Description of the trial

This is a single-centre three arm randomised controlled feasibility pilot comparing Parent-Administered Screen Time Intervention (PASTI) against a dummy Bedtime-Box arm and a No-Intervention arm in 17-31-month-old infants who have parent-reported screen time in the hour before bed. Participating families will be recruited from Central/Greater London and surrounding areas and undergo baseline assessments at home and in the lab at the Centre for Brain and Cognitive Development (CBCD) at Birkbeck, University of London. Participants will be randomly allocated (1:1:1) to one of three treatment arms: PASTI, Bedtime-Box, or No-Intervention. Follow-up home and lab assessments will be collected 6-7 weeks after baseline. The main objective of this feasibility trial is to estimate parameters required for planning a definitive trial (e.g. recruitment and randomisation rates). Secondary objectives include obtaining preliminary effect estimates of PASTI on toddler screen time, sleep, and attention control outcomes. More details are provided in the trial protocol.

This statistical analysis plan (SAP) describes the quantitative analyses that will be carried out by the trial statisticians. This does not include qualitative analysis from interviews that will be analysed separately. The statistical analysis plan was drafted and reviewed by blinded trial statisticians per the King's Clinical Trials Unit (KCTU) Standard Operating Procedures (SOP) related to statistical analysis plans [ST-02 Version 3.0]. The trial team will follow KCTU SOPs.

1.1 Principal research objectives to be addressed

1.1.1 Primary objectives

The primary aim of the trial is to assess the feasibility of implementing PASTI, compared with a Bedtime Box intervention and No Intervention, in 17-31-month-old infants who have parent-reported screen time in the hour before bed. To fulfil our primary aim, we will:

1. Assess the feasibility of conducting PASTI, including participation rate, participant randomisation, participant attrition, and intervention adherence.
2. Gain insight into parent experiences of PASTI and any barriers to adherence.
3. Estimate statistical parameters of the primary outcomes necessary to power a future large-scale, confirmatory trial, e.g. means and standard deviations of continuous outcome measures: total night-time sleep duration (actigraphy); exogenous attention control (singleton search saccadic reaction time from the Visual Search Task)

1.1.2 Secondary objectives

Our secondary aims and objectives are to collect preliminary effect size estimates for PASTI's impact on toddler sleep and attention, as well as preliminary effect size estimates for PASTI on secondary sleep (e.g. average nap duration, frequency of night awakenings, sleep onset-latency), attention

(e.g. exogenous and endogenous attention control measures) and screen time outcomes (e.g. average toddler screen use in the hour before bed).

1.2 Trial design

This is a single-centre 1:1:1 randomised controlled trial with three parallel intervention groups, assessing the feasibility of implementing a 7-week Parent-Administered Screen Time Intervention (PASTI) compared to a dummy Bedtime-Box arm and a No-Intervention arm. Descriptions of the trial interventions can be found in the protocol. This feasibility trial will aid in planning a future definitive trial estimating the effect of PASTI on infant attention control and sleep outcomes.

The study assessments take place within the CBCD Babylab and in families' homes. All baseline and follow-up lab assessments will take place in the CBCD Babylab, and all baseline and follow-up home assessments will be completed in each families' home. Families will be allocated to an intervention group and provided with any study material which will be used in each families' home.

1.2.1 Participant enrolment

Families will be recruited via several routes (e.g. CBCD, Early Years Alliance child centres, National Childbirth Trust and Sleep Charity social media platforms and membership databases). Each route will advertise the trial using a physical or online poster that contains a link where families can sign up to take part. Interested families will provide electronic informed consent and complete a pre-screening questionnaire. Study researchers will review the pre-screening questionnaires and contact eligible families to provide full consent for the study and schedule the baseline lab visit.

In the week before the baseline lab visit, participants will undergo baseline home and actigraphy assessments, followed by a baseline lab visit at CBCD. Participants will be randomised at the end of the baseline lab visit by the unblinded researcher.

1.2.2 Post-randomisation

During weeks 1 to 7 after randomisation, participants will complete a bedtime activity diary twice weekly: once on a weekday, and once on a weekend. Participants in the PASTI arm will complete a screen time adherence questionnaire every night during weeks 1 to 7 (except on nights that the bedtime activity diary is administered).

During week 6, after randomisation, participants will complete follow-up home assessments (questionnaires and actigraphy). Participants will attend a follow-up lab visit 7 weeks after randomisation (with a two-week visit window of the expected date).

After the follow-up lab visit in week 7, participants in the PASTI and Bedtime Box arms will be sent a debrief questionnaire.

Baseline and follow-up lab assessments will be conducted by blinded outcome assessors. See Section 1.4 below for further details regarding blinding or refer to the trial protocol.

1.3 Eligibility criteria

After completing the pre-screen questionnaire, participants must meet the following criteria prior to randomisation:

1. A family with an infant between 16 and 30 months at pre-screening (enrolment)
2. A family that lives in Central/Greater London and surrounding areas (within 75 miles of the Birkbeck Babylab)
3. A family who reports that their infant uses screen time in the hour before bed. They must report that their infant uses ≥ 10 minutes of screen time in the hour before bed ≥ 3 days of the week
4. Caregiver able to provide informed consent.

Reports of screen time use in the hour before bed from baseline questionnaires will not be used to exclude participants that had met eligibility requirements at pre-screening.

Exclusion criteria:

1. An infant with any known genetic or neurological condition (e.g., Downs Syndrome)
2. An infant born prematurely (< 37 weeks)
3. An infant who is taking part in another trial or research study

1.4 Randomisation and blinding

1.4.1 Randomisation

At the end of the baseline lab visit, participants will be randomised to one of the three treatment arms by the unblinded researcher. Randomisation will be done using an Elsevier web-based randomisation system set up by the King's Clinical Trials Unit (KCTU) at King's College London following KCTU standard operating procedures.

The sequence will be generated using minimisation to ensure balance between trial arms. Minimisation will balance across: child sex, socioeconomic status (IMD quintile), and child age at randomisation (17-24.4 months or 24.5-31 months).

The sequence will be allocated by the unblinded researcher who will enter the participant randomisation details (e.g. child's age, sex, IMD quintile) into the randomisation system and will receive details of the treatment allocation along with a unique participant identifier from the randomisation reference ID that will be used to identify participants in the research database. The unblinded researcher will then inform the participating family of their treatment allocation. Details of treatment allocation will not be shared with the other research staff members. Follow-up outcomes will be assessed by a blinded researcher.

1.4.2 Blinding and planned unblinding

Due to the nature of the interventions, participants will not be blind to treatment allocation. Participants will be asked not to discuss treatment groups with the blinded outcome assessor.

The junior trial statistician will be blind to the group allocation until trial analysis begins (and at the point of the Statistical Analysis Plan sign off). The chief investigators and the senior statistician will be blind until review of the draft trial report after database lock. The trial steering committee will be blinded and only receive open reports during meetings. The trial steering committee chair may request a closed unblinded report if there are safety concerns.

1.5 Description of intervention period and frequency of follow-up

The Intervention period in all arms starts from the Monday following the baseline Lab visit and ends at the follow-up Lab visit 7 weeks later. Starting from the week of baseline home assessments and up to the week of the follow-up lab visit, participants will complete twice-weekly activity diaries. Participants in the PASTI arm will also complete brief daily adherence questionnaires in between the baseline and follow-up Lab visits.

1.6 Visit windows

The follow-up lab visit should occur during week seven of the trial, with a two-week visit window for lab assessments. Questionnaires from baseline and follow-up home assessments (e.g. Brief Infant Sleep Questionnaire, Vineland Adaptive Behavior Scale) should be completed before the corresponding Lab visit.

1.7 Data collection

All questionnaires will be administered electronically through a web survey platform (REDCap). Data from actigraphy watches and eye-tracking lab assessments will also be captured electronically and processed as described in the protocol. Adverse events are collected through a web-form completed by participants at any point during study participation.

Data collection timepoints are outlined below. Further details of specific measures can be found in the trial protocol, a summary is provided in the Appendix.

	STUDY PERIOD											
	Prescreen	Enrolment		Allocation	Post-allocation							Close-out
Timepoint/Week (W) #	before W0	W-1	W0*	W0	W1	W2	W3	W4	W5	W6	W7	W7+
**ENROLMENT (ALL ARMS)**												
Informed consent	X											
Eligibility screening	X											
Probabilistically sampled	X											
Allocation (randomised)				X								
**INTERVENTIONS**												
PASTI Arm					X	X	X	X	X	X	X	
Bedtime Box Arm					X	X	X	X	X	X	X	
No Intervention Arm					X	X	X	X	X	X	X	
**HOME ASSESSMENTS (ALL ARMS)**												
Daytime Activity Levels Questionnaire		X								X		
Vineland Adaptive Behavior Scale (VABS)		X								X		
Brief Infant Sleep Qestionnaire (BISQ)		X								X		
Early Childhood Behaviour Questionnaire (ECBQ)		X								X		
State and Trait Anxiety Inventory (STAI)		X								X		
Sleep Actigraphy watch		X	X							X	X	
Sleep and Motion Watch Diary		X	X							X	X	
**LAB ASSESSMENTS (ALL ARMS)**												
Mullen Scales of Early Learning			X								X	
Visual Search Task			X								X	
Gap-Overlap Task			X								X	
Antisaccade Task			X								X	
**OTHER ASSESSMENTS**												
Daily Screen Time questionnaire (PASTI only)					X	X	X	X	X	X	X	
Bedtime Activity Diary (all arms)		X	X		X	X	X	X	X	X	X	
Parent Debrief Questionnaire (PASTI and Bedtime Box arms only)												X
Parent Debrief Interview (subset of PASTI arm only)												X
Adverse Events (all arms)		X	X	X	X	X	X	X	X	X	X	X

*Lab measures conducted at baseline lab before randomisation occurs at the end of the visit

1.8 Sample size estimation (including clinical significance)

In line with guidance on feasibility studies, no power calculation has been carried out [1]. A sample size of 60 to 100 has been shown to be sufficient in a feasibility trial [2]. A target sample size of 105 (N=35/arm) will be enrolled and randomised.

1.9 Primary feasibility and secondary outcome measures

To fulfil the study objectives outlined in Section 1.1, the following outcome measures will be assessed (excluding qualitative analyses for #4 and #5):

1.9.1. Primary feasibility outcome measures:

1. **Participation rate:**
    - Number of eligible participants
    - Number of participants consenting to the full study
    - Proportion of consented participants who are eligible to take part
    - Distribution of socioeconomic profiles for families enrolled relative to geographic norms (e.g. Central/Greater London)

In addition, the proportion randomised out of those eligible will be reported.

2. **Intervention adherence (in the PASTI arm):**

    - Average proportion of caregivers that reported no screen time in the hour before bed during the intervention period
3. **Participant retention:**
    - Number and proportion of participants attending the follow-up lab visit out of those randomised overall and by trial arm
    - Number and proportion of participants that complete all follow-up home and lab assessments out of those randomised overall and by trial arm
4. **Participant experiences:** The distribution of parent experience ratings from debrief surveys completed by PASTI arm participants.
5. **Assessment acceptability:** The distribution of study assessment acceptability ratings from debrief surveys completed by PASTI arm participants.

The distribution of parent experience ratings and study assessment acceptability ratings from debrief surveys completed by Bedtime Box arm participants will also be summarised.

1.9.2 Secondary outcomes:

We will estimate preliminary PASTI effect sizes on the following outcomes at the Weeks 6-7 follow-up home and lab visits:

1. Total infant night-time sleep duration (minutes) using actigraphy (averaged across the 7-9 day actigraphy period immediately preceding the lab visit)
2. Exogenous attention control (single search saccadic reaction time from the Visual Search Task)
3. Average toddler screen use (minutes) in the hour before bed (measured using responses from one weekday and one weekend bedtime activity diary directly preceding the lab visits and with the following formula: [average weekday screentime *5]+[average weekend screentime *2] / 7)
4. Additional sleep outcomes:
    a. Average day-time nap duration (minutes) using actigraphy
    b. Frequency of night awakenings using actigraphy (averaged across the 7-9 day actigraphy period immediately preceding the lab visit)
    c. Sleep efficiency (total night time sleep duration expressed as a percentage of time in bed, averaged across the 7-9 day actigraphy period immediately preceding the lab visit) using actigraphy
    d. Parent-reported sleep onset latency (minutes) from the BISQ-R
5. Additional attention control outcomes (reaction times in milliseconds):
    a. Prosaccade saccadic reaction time from the Antisaccade Task
    b. Proportion of antisaccades from the Antisaccade Task
    c. Baseline saccadic reaction time from the Gap-Overlap Task
    d. Disengagement saccadic reaction time from the Gap-Overlap Task
    e. Parent-reported inhibitory control subscale score from the ECBQ
    f. Parent-reported effortful control global score from the ECBQ

2. Quantitative Analysis Plan

This plan covers any quantitative analyses for the feasibility trial. The trial will also undertake some qualitative research which will not be covered in this plan.

2.1 Overview

This feasibility trial will estimate parameters needed for planning an efficacy trial including various rates (e.g. recruitment, randomisation, losses to follow-up) and the within-trial arm means and standard deviations for possible outcome measures to inform the sample size calculation of a future randomised controlled trial. Scores for published questionnaires will be summarised for each measure, and outcome measures will be described by trial arm. Intervention effect sizes (e.g. mean differences for continuous outcome measures at follow-up between arms) will be estimated for all secondary outcome measures as previously described, but no formal hypothesis testing will be carried out. All analyses will be based on the modified intention-to-treat principle. The final statistical report will be produced according to KCTU SOP ST-07 (Statistical Reports, V3.0).

2.2 Recruitment and representativeness of recruited patients

In addition to the participation outcomes listed in section 1.9.1, we will require the following information to construct the CONSORT flowchart (see template flowchart in Figure 1):

- Number of potentially eligible participants completing pre-screening questionnaire
- Number of participants deemed ineligible, and reasons why
- Number of participants withdrawing and/or lost to follow-up between pre-screening and baseline home assessments
- Number of participants continuing through trial
- Number of participants withdrawing and reasons why
- Number of participants excluded from analysis and reasons why

The representativeness of recruited patients will be shown through descriptive summaries of socio-economic/demographic data.
The trial will be reported according to the CONSORT extension guidelines for pilot trials [3].

Figure 1. Template CONSORT diagram for PASTI feasibility trial

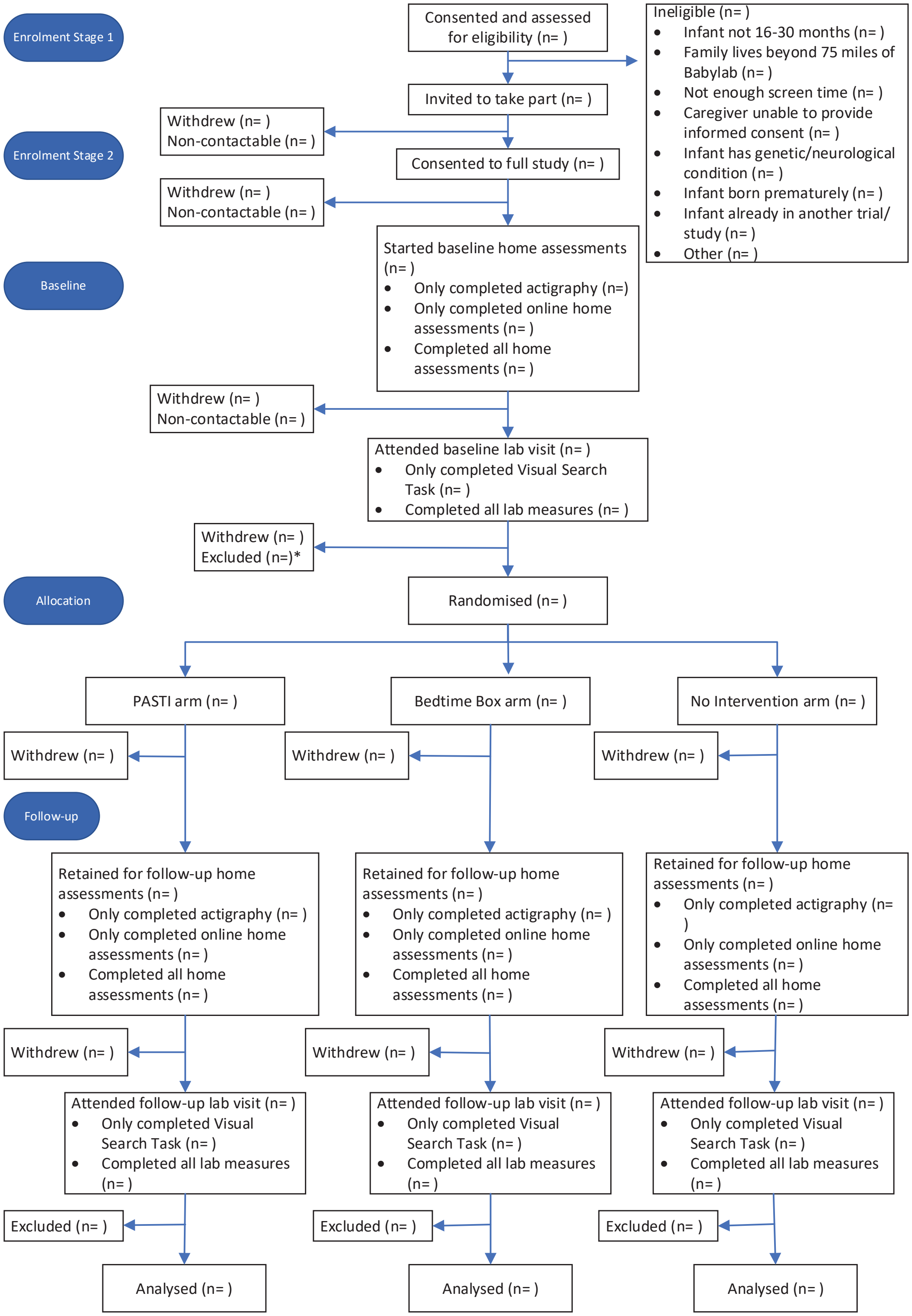

2.3 Description of sample characteristics at baseline

To describe the study sample and check that the trial arms are balanced, demographic and baseline variables will be summarised by trial arm and across the total study sample. Summary statistics will be calculated (means and standard deviations [SDs] for continuous outcomes if normally distributed, medians and quartiles if skewed; frequencies and proportions for discrete outcomes). The randomisation of intervention groups to participants should have ensured that any imbalance over all measured and unmeasured baseline characteristics is due to chance. Therefore, no significance testing will be conducted to assess evidence of group differences for these baseline variables.

2.4 Estimation of feasibility parameters

In addition to drafting the CONSORT participant flowchart, the following details will be reported along with appropriate 95% confidence intervals (CIs):

1. **Participation rate:**
   - Number and proportion of participants consenting to the full trial out of those eligible and invited to take part.
   - Distribution of socioeconomic profiles for families enrolled relative to geographic norms (e.g. Central/Greater London)

   The number and proportion of randomised participants out of those deemed eligible and were invited to take part will also be reported.
2. **Intervention adherence (in the PASTI arm):**
   - Average proportion of parents that reported no screen time in the hour before bed during the intervention period
     - First, the proportion of ‘no screen time’ reported out of the number of daily adherence questionnaires completed will be calculated for each PASTI arm participant. Then, this proportion will be averaged across all participants randomised to PASTI
3. **Participant retention:**
   - Number and proportion of participants attending the follow-up lab visit out of those randomised overall and by trial arm
   - Number and proportion of participants that complete all follow-up outcome home and lab assessments out of those randomised overall and by trial arm
4. **Parent experiences (in the PASTI arm):**
   - Frequency and proportion of parent experience ratings from debrief survey completed by PASTI arm participants
5. **Assessment acceptability (in the PASTI arm):**
   - Frequency and proportion of parent study assessment acceptability ratings from debrief survey completed by PASTI arm participants

The distribution of parent experience ratings and study assessment acceptability ratings from debrief surveys completed by Bedtime Box arm participants will also be summarised.

We have chosen to implement a ‘traffic light’ system to assess the feasibility of a full-scale PASTI trial. Performance metrics that fall in the Red zone

indicate that a full trial following the current design may not be feasible, metrics that fall in the Amber zone indicate that a full trial may be feasible but the protocol should be modified or the situation monitored closely, and metrics that fall in the Green zone indicate that a full trial is feasible and we may continue without substantial modifications to the current study design.

Metric	Red zone	Amber zone	Green zone
**Randomisation** (Number of participants randomised overall)	≤ 73	74 to 104	≥ 105
**PASTI daily questionnaire completion** (% of participants randomised to PASTI and retained to lab follow-up that complete ≥ 60% of daily screen time questionnaires)	< 65%	65% to 79%	≥ 80%
**PASTI adherence to screen time removal (week 1 – week 6)** (% of participants randomised to PASTI that report no screen time on ≥ 60% of daily screen time questionnaires completed)	< 50%	50% to 69%	≥ 70%
**PASTI debrief questionnaire completion** (% of participants randomised to PASTI that complete the debrief questionnaire measuring participant experience and assessment acceptability)	< 65%	65% to 74%	≥ 75%
**Retention** (% of randomised participants attending follow-up Lab visit)	< 70%	70% to 74%	≥ 75%

2.5 Description of follow-up outcome measures

Follow-up outcome measures (see Appendix for details) will be summarised by trial arm and for the total sample using descriptive statistics.

2.6 Secondary outcomes

2.6.1 Descriptive analysis

The secondary outcomes listed in Section 1.9.2 will be summarised using descriptive statistics at baseline and follow-up timepoints.

2.6.2 Treatment Effects

2.6.2.1 Continuous outcome data

Outcomes will be analysed with crude and an adjusted multivariable linear regression analysis. Fixed effects included will be: dummy treatment variables; baseline values of continuous outcomes; minimisation factors (child age group, child sex, family IMD quintile).

The treatment group mean difference (MD) for unstandardised effect sizes and 95% confidence intervals will be presented for both crude and adjusted estimates. Given the sample size and purpose of this feasibility trial, these estimates will not the basis for inferential statements and p-values will not be reported. The purpose of our effect size estimates is to illustrate the size of effects across a number of possible outcomes.

2.6.2.2 Binary outcome data

Any binary outcomes will be analysed with crude and an adjusted multivariable logistic regression analysis. Fixed effects will include the baseline values of binary outcomes; minimisation factors (child age group, child sex, family IMD quintile). Summary effects will be reported as crude odds ratio and adjusted odds ratio (aOR) with 95% CI. No p-values will be reported.

2.6.2.3 Count outcome data

Frequency of night awakenings is likely to be a highly skewed count variable (measured as the average number of night awakenings over the week-long actigraphy period). The distribution of night awakenings will be examined to determine which zero-inflated regression model would be most appropriate (i.e. if count data is overdispersed, a negative binomial model may be used instead of a Poisson regression model). Incidence rate ratios (IRRs) will be reported with 95% CIs. No p-values will be reported.

2.6.3 Estimands

Defining an estimand framework was considered for this trial. Since this is an early-phase feasibility trial where the primary aim is to assess the feasibility of implementing a full PASTI trial, we will not focus on defining an estimand framework at this time. The results of this feasibility trial will help inform definitions of populations, intercurrent events, and other estimand framework attributes for a full PASTI trial.

2.6.4 Primary comparisons of between-group differences

The following primary comparisons will be reported for each secondary outcome:

1. PASTI vs Bedtime Box
2. PASTI vs No Intervention

2.6.5 Distributional assumption checks

Modelling assumptions will be checked using visual methods (i.e. plots of residuals to check if residuals are normally distributed, scatterplots of standardised residuals to check homoscedasticity).

2.7 Populations under investigation

The primary population under investigation will be the modified intention to treat population. This will include participants who have completed at least one post baseline assessment time-point. Missing data will be explored and may be dealt with in a manner consistent with Jakobsen et al [4].

2.8 Loss to follow-up and other missing data

2.8.1 Missing demographic and questionnaire data

All efforts will be made to avoid missing data (i.e., sending reminders to complete questionnaires, incentives to return for follow-up). For each demographic and outcome measure at baseline and follow-up, the frequency and percentage of missing items will be reported for the total sample and by trial arm.

If there is missing baseline data, we will take an appropriate approach recommended by literature (e.g. mean imputation as described by White and Thompson [5]) before performing regression models as described above.

The number of completed twice-weekly activity diaries will be reported for the total sample and by trial arm. The proportion of completed diaries out of the total number of diaries sent will also be reported.

For the PASTI arm participants that have been asked to complete daily adherence questionnaires, the number of completed daily adherence questionnaires will be reported along with the proportion of completed questionnaires out of the total number of questionnaires sent.

2.8.2 Missing items in scales and subscales

Where available, we will use missing value guidance provided for scales. Where this is not available and prorating seems appropriate, we will prorate missing items only when there are no more than 30% missing items (i.e. for a ten item questionnaire, prorate only where one to three items are missing) by replacing the missing item values with the mean value of the complete items for each individual. We will not prorate missing items in calculating VABS subdomain scores because this would affect the basal/ceiling scoring rules.

2.8.3 Missing items in lab and actigraphy measures

During baseline and follow-up home assessments, parents will be asked to fit actigraphy watches to their infant's ankle over a 7-9 day period. If data from actigraphy watches is missing, we may prorate if data is available from 70% of the home assessment period (i.e. if data on total infant night-time sleep duration is available from 7 out of 9 days, replace missing item values with the average value of the complete items for each individual).

2.8.4 Withdrawals and losses to follow-up

The reasons for withdrawal from the trial will be summarised overall and by treatment arm if available.
In addition to describing the number of participants withdrawing and/or lost to follow-up (i.e., not returning for subsequent assessments) between pre-screening and baseline home assessments, withdrawals and/or losses to follow-up will also be described for the time periods:

- Between randomisation and weekday activity diary in week 4
- Between the weekday activity diary in week 4 and the follow-up Lab visit

2.9 Pre-processing of actigraphy and eye-tracking data

2.9.1 Actigraphy data

Pre-processing of Actigraphy data will be done in the CamNtech MotionWare Software. The following data will be excluded before analysis:

- Periods of non-wear will be excluded using the MotionWare algorithm and parent-reported Actigraph removal on the Sleep Diary.
- Periods of external movement (e.g. car/buggy) will be excluded using the parent-reported Sleep Diary.

Actigraphy sleep analysis will be conducted using the CamNtech MotionWare software to deduce our primary and secondary sleep metrics. Daytime nap data will be excluded if any of the following are observed:

- A period of non-wear was reported during the nap period
- A period of external movement was reported during the nap period
- The parent reported that the child's day was not typical (e.g. child illness, child sleeping in different location, disturbance) using the parent-reported Sleep Diary.

Night time sleep data will be excluded if any of the following are observed:

- A period of non-wear was reported during the sleep period
- A period of external movement was reported during the sleep period
- The parent reported that the child's night was not typical (e.g. child illness, child sleeping in different location, disturbance) using the parent-reported Sleep Diary.
- The sleep period occurred on Daylight Saving Time (e.g. 30/10/2022)

Actigraph data for each participant will be excluded from all analysis if there are < 3 days day or night data, respectively for nap or sleep analysis.

2.9.2 Eye-tracking data

<u>2.9.2.1 Visual Search Task</u>

A Trial in the Visual Search Task will be classed as valid if:

- A saccade towards the central fixation is made, followed by a saccade towards the target apple within 4000ms of the search array presentation.

A trial in the Visual Search Task will be classed as invalid if:

- No saccade towards the central fixation is made. Trials where no saccade towards the central fixation is made are recycled.
- No saccade towards the target is made.

The Visual Search Task per participant will be excluded if there are less than 5 valid trials. Trials will be classified into Single Search and Conjunction Search based on stimulus presentation. Reaction times for valid trials in the Single Search condition will be averaged to create our Single Search saccadic reaction time secondary outcome variable.

<u>2.9.2.2 Anti-Saccade Task</u>

A trial in the Anti-Saccade Task will be classed as valid if:

- A saccade towards the distractor was made within 400ms of its presentation and the target was not fixated. This trial is labelled as a Pro-Saccade.
- A saccade towards the distractor was made within 400ms of its presentation and an anticipatory saccade to the target before or within 100ms of it being presented. This trial is labelled as a Corrective-Saccade.
- No saccade towards the distractor was made and a saccade to the target was made before or within 100ms of it being presented. This trial is labelled as an Anti-Saccade.
- A saccade towards the distractor was made within 400ms of its presentation and then a saccade was made towards the target > 100ms after it was presented. This trial is labelled as Pro-Saccade Reactive.

A trial in the Anti-Saccade Task will be classed as invalid if:

- No saccade towards the distractor is made and a saccade towards the target is made after it was presented (>100ms after the target presentation).
- The distractor was fixated between 400 and 1000ms after it was presented and the target was not fixated.

- The distractor was fixated between 400 and 1000ms after it was presented and an anticipatory saccade to the target was made (≤ 100ms after the target presentation).
- The distractor was fixated between 400 and 1000ms after it was presented and the target was fixated after it was presented (> 100ms after the target presentation).
- No distractor saccade or target saccade is made. Trials where no saccade was made are recycled.

The Anti-Saccade Task per participant will be excluded if there are less than 5 valid trials. Reaction times for valid Pro-Saccade and Pro-Saccade Reactive trials will be averaged to create our Pro-Saccade saccadic reaction time secondary outcome variable. The number of valid Anti-Saccades will be divided by the total valid trials and multiplied by 100 to create our proportion of Anti-Saccades secondary outcome variable.

2.9.2.3 Gap-Overlap Task

A trial in the Gap-Overlap Task will be classed as valid if:

- A saccade towards the central fixation was made, followed by a saccade towards the target peripheral stimulus within 2500ms of the peripheral target being presented.

A trial in the Gap-Overlap Task will be classed as invalid if:

- The target peripheral stimulus is presented on the vertical axis.
- No saccade towards the central fixation is made. Trials where no saccade towards the central fixation is made are recycled.
- No saccade towards the target peripheral stimulus is made.
- A saccade toward the wrong side of the screen is made.

The Gap-Overlap Task per participant will be excluded if there are less than 5 valid trials. Trials will be classified into Baseline, Gap and Overlap Trials based on stimulus presentation. Reaction times for valid Baseline trials will be averaged to compute our Baseline saccadic reaction time secondary outcome variable. Reaction times for valid Overlap trials will be averaged to compute an Overlap saccadic reaction time variable. Baseline saccadic reaction times will be subtracted from Overlap saccadic reaction times to compute our Disengagement saccadic reaction time secondary outcome variable.

2.10 Adverse event reporting

Adverse events (AE), adverse reactions (AR), serious adverse events (SAE) and serious adverse reactions (SAR) will be summarised overall and by treatment arm as counts of events and counts of people who have had events. Adverse events will also be described by severity (mild/moderate/severe) and by relatedness (see definitions in protocol).

Adverse events will not be powered and only will be presented descriptively, unless directed by the oversight committee on reflection of findings.

2.11 Sensitivity and other analyses

An additional analysis may be carried out to those planned to test an additional assumption made, or as indicated by the trial oversight committees.

If baseline characteristics across trial arms appear to be unbalanced (i.e., through examination of means and standard deviations and if there is a difference of >0.5 SD), we may consider a sensitivity analysis where our regression models for secondary outcomes also include the unbalanced baseline characteristics as an additional sensitivity analysis.

For additional infant sleep outcomes and additional attention control outcomes (secondary outcomes #4 and #5), seemingly unrelated regression may be considered if appropriate.

In an exploratory analysis, the effect of Bedtime Box vs No Intervention will be estimated for each secondary outcome and may be reported in supplementary materials in the main trial results.

2.11.1 Interim and subgroup analyses

No formal interim analyses or subgroup analyses have been planned for this study.

2.12 Protocol adherence

2.12.1 Protocol violators

Protocol violators (PV) are defined as participants that breach the protocol in a way that may lead to a change in study findings. Potential examples include:

- Family or index child participating in another study during the trial
- Family reporting that index child was medicated for sleep apnoea

A per-protocol analysis excluding protocol violators may be performed as a sensitivity analysis. All differences in outcomes between arms will be identified as preliminary and underpowered when published.

2.12.2 Protocol deviators

Protocol deviations (PD) are defined as events where participants fail to adhere to the protocol in a way that may have a minor or no impact on study findings. Potential examples include:

- Participants complete the follow-up lab visit outside of the 2-week visit window
- Participant incorrectly enrolled (i.e. incorrect eligibility information provided)

A summary of protocol deviations may be reported using descriptive statistics (i.e. the number and percentage of participants with protocol deviations overall and by trial arm).

2.13 Data monitoring

Once the initial SAP has been approved by the TSC, the junior statistician will be able to review trial data by treatment arm. Two of the success metrics for study feasibility are based on responses to the daily screen time questionnaires in the PASTI arm (PASTI adherence to screen time removal and PASTI daily questionnaire completion). To monitor intervention adherence and adherence questionnaire completion in the PASTI arm, reports of intervention adherence and questionnaire completion will be provided to the TSC chair in a closed session at appropriate timepoints (i.e., when at least 20% of participants have been recruited, and then again when at least 50% of participants have been recruited).

As part of data processing, range and validity checks will be performed before entry into the research database. In advance of TSC meetings, the junior statistician will perform a database extract and issue any queries (i.e. confirming values with research team if greater/less than 3 SDs) to be resolved by the Birkbeck research team. The procedures and guidelines detailed in KCTU SOPs ST-01 (Data Monitoring Committees, V3.0), ST-04 (Statistical QA, V2.0), and ST-08 (Data manipulation following Data Extraction, V2.1) will be followed.

2.14 Data checking

During data processing, designated blinded study researchers will perform range and valid value checks on questionnaire, eye-tracking, and actigraphy data. In preparation for oversight committee meetings, the junior statistician will review the data extract and issue queries as needed. In addition to eligibility criteria, the list of items to be checked includes (but is not limited to): dates, calculated ages, missing data, potential outliers, and if all necessary forms have been completed.

2.15 Software

Questionnaires will be collected via a secure online survey platform (REDCap). Actigraphy and eye-tracking data will be collected and processed on a secure Microsoft laptop. Study data (questionnaire responses, actigraphy, summary eye-tracking data, etc.) will be entered by designated blinded researchers into a separate REDCap research database. Data collected from only PASTI arm participants (i.e. adherence questionnaire responses) will be stored in a separate database accessible only by unblinded researchers. Once all data has been entered and any queries have been resolved, the research database will be locked and an extract provided to the trial statistician for analysis. The debrief survey responses collected from PASTI and Bedtime Box arm participants will be provided at the end of the study to the trial statistician for analysis. The final randomisation extract will also be requested and provided to the trial statistician after database lock. Full details of data management can be found in the trial protocol.

Data analysis will be conducted on Stata version 17.0 (or later). R version 4.1.1 (or later) may be used for data visualisation purposes. The KCTU web-based randomisation system will be used.

2.16 Quality Control and assurance

The KCTU Statistics SOPs will be followed. Specific SOPs have been noted throughout this document and are listed here:

- ST-01: Data Monitoring Committees (V3)
- ST-02: Statistical Analysis Plan (V3)
- ST-03: Statistical Document Retention (V3)
- ST-04: Statistical QA (V2)
- ST-05: Sample size calculation (V3)
- ST-06: Protocol Review and Blinding (V3.1)
- ST-07: Statistical Reports (V3)
- ST-08: Data manipulation following data extraction (V2.1)

In addition, we will follow KCTU QS-04: Control of non-conformities (V3).

Reference list

(1) Arain, M. Campbell, M.J., Cooper, C.L., & Lancaster, G.A. (2010). What is a pilot or feasibility study? A review of current practice and editorial policy. *BMC Medical Research Methodology, 10*(1), 67.

(2) Teare, M.D., Dimairo, M. Shephard, N., Hayman, A., Whitehead, A., & Walters, S.J. (2014). Sample size requirements to estimate key design parameters from external pilot randomised controlled trials: a simulation study. *Trials, 15*(1), 264.

(3) Eldridge, S.M., Chan, C.L., Campbell, M.J., Bond, C.M., et al. (2016). CONSORT 2010 statement: extension to randomised pilot and feasibility trials. *Pilot Feasibility Stud, 2*(1), 64.

(4) Jakobsen, J.C., Gluud, C., Wetterslev, J., & Winkel, P. (2017). When and how should multiple imputation be used for handling missing data in randomised clinical trials – a practical guide with flowcharts. *BMC Medical Research Methodology, 17*(1), 162.

(5) White, I.R., Thompson, S.G. (2005). Adjusting for partially missing baseline measurements in randomized trials. *Stat Med, 24*(7), 993.

Appendix 1: Description of study measures

Pre-screening demographic measures

- Recruitment method
- Child age
- Child sex
- Child ethnicity
- Child medical conditions
- Completed weeks of pregnancy when child was born
- Days a week child is in nursery/childcare
- Number of younger and older siblings for target child
- Caregiver sex
- Caregiver age
- Caregiver ethnicity
- Caregiver educational level
- Family postcode and IMD quintile
- Screen time use in the hour before bed
    - If screen time use reported, frequency (days in a week) and duration (minutes)
- If caregiver respondent is sole parent/caregiver
- Child’s total daytime sleep duration (minutes) during past two weeks
- Usual sleep onset latency for child during past two weeks
- Usual frequency of night awakenings during past two weeks
- Child’s total night-time sleep duration (minutes) during past two weeks

Baseline and follow-up home assessment measures

- Presence and duration (minutes) of daily activities as reported in Daytime Activity Levels Questionnaire for daily active play, daily passive play, screen time
- Infant sleep practices (including sleep onset latency) as reported in Brief Infant Sleep Questionnaire – Revised (BISQ-R)
- Infant temperament subscale scores (effortful control and inhibitory control) as reported in a modified version of the Early Childhood Behaviour Questionnaire (ECBQ)
- Vineland Adaptive Behaviour Scale (VABS) subdomain raw and scaled scores for receptive language, expressive language, reading/writing, overall socialisation, overall motor skills
- Caregiver state anxiety (total score of the 20 items) from the State and Trait Anxiety Inventory (STAI)
- Infant actigraphy data: night-time sleep duration, night-time awakening frequency, day-time nap duration, sleep efficiency (total night-time sleep duration expressed as a percentage of time in bed, on a scale of 0 to 100%)
- Parent-reported sleep log: watch worn all night, watch worn all day, timing of watch removals

Baseline and follow-up lab assessment measures

Reaction times from lab eye-tracking activities will be reported in milliseconds.

- Visual Search Task:
    - Single search reaction time
    - Conjunction search reaction time
- Antisaccade Task:
    - Prosaccade reaction time
    - Proportion of pro-saccades
    - Proportion of antisaccades
    - Proportion of corrective saccades
- Gap-overlap Task:
    - Baseline reaction time
    - Overlap latency reaction time
    - Gap latency reaction time
    - Disengagement (overlap latency – baseline latency) reaction time
    - Facilitation (gap latency – baseline latency) reaction time
- Mullen Scales subscale scores

Additional measures

- Adverse events collected throughout study participation via web-form
- Whether or not child has been taking sleep medication, and if child takes sleep medication frequently (asked during lab visits)
- Twice-weekly (one weekday, one weekend) bedtime activity diaries capturing child activities in the hour before bed
- For PASTI and Bedtime Box arms: online debrief questionnaires at the end of the trial examining parent experiences and challenges encountered
- PASTI arm only: short daily screen time adherence questionnaire assessing screen time duration, device, and reason for use
