# Supplementary material for: Toddler Screen Use Before Bed and Its Effect on Sleep and Attention: A Randomized Clinical Trial
Source: JAMA Pediatr. 2024 Oct 21;178(12):1270–9. doi: 10.1001/jamapediatrics.2024.3997 (PMC11581737; doi:10.1001/jamapediatrics.2024.3997)
Supplement: Supplement 3. — eFigure 1. Participant Timeline eAppendix 1. Eye-Tracking Experiment Details eFigure 2. Stimulus Sequence for Experimental Trials in the Visual Search Task eFigure 3. Stimulus Sequence for Experimental Trials in the Antisaccade Task eFigure 4. Stimulus Sequence for Experimental Trials in the Gap-Overlap Task eAppendix 2. Intervention Delivery eAppendix 3. Family Bedtime Box eFigure 5. Family Bedtime Box Materials eAppendix 4. Parent-Administered Screen Time Intervention (PASTI) Development eAppendix 5. Screen Use Questions Used in the Screen Use Questionnaire and Bedtime Activity Diary eAppendix 6. Parent-Reported Screen Time Measure eTable 1. Descriptives for Baseline and Follow-Up Secondary Outcomes eTable 2. Descriptives for Alternative Activities in the Hour Before Bed From the Bedtime Activity Diary eFigure 6. Forest Plot of Effect Sizes for Bedtime Box vs No Intervention Comparison eReferences [file jamapediatr-e243997-s003.pdf]

Supplementary Online Content

Pickard H, Chu P, Essex C, et al. Toddler screen use before bed and its effect on sleep and attention: a randomized clinical trial. *JAMA Pediatr*. Published online October 21, 2024. doi:10.1001/jamapediatrics.2024.3997

eFigure 1. Participant Timeline

eAppendix 1. Eye-Tracking Experiment Details

eFigure 2. Stimulus Sequence for Experimental Trials in the Visual Search Task

eFigure 3. Stimulus Sequence for Experimental Trials in the Antisaccade Task

eFigure 4. Stimulus Sequence for Experimental Trials in the Gap-Overlap Task

eAppendix 2. Intervention Delivery

eAppendix 3. Family Bedtime Box

eFigure 5. Family Bedtime Box Materials

eAppendix 4. Parent-Administered Screen Time Intervention (PASTI) Development

eAppendix 5. Screen Use Questions Used in the Screen Use Questionnaire and Bedtime Activity Diary

eAppendix 6. Parent-Reported Screen Time Measure

eTable 1. Descriptives for Baseline and Follow-Up Secondary Outcomes

eTable 2. Descriptives for Alternative Activities in the Hour Before Bed From the Bedtime Activity Diary

eFigure 6. Forest Plot of Effect Sizes for Bedtime Box vs No Intervention Comparison

eReferences

This supplementary material has been provided by the authors to give readers additional information about their work.

© 2024 Pickard H et al. *JAMA Pediatrics*.

Timeline	Baseline (Pre-weeks 1 & 2)		Intervention (Weeks 1 to 5)					Follow up (Weeks 6 & 7)		
	Week 1	Week 2	Week 1	Week 2	Week 3	Week 4	Week 5	Week 6	Week 7	Week 7+
Interventions										
PASTI Intervention group			Δ	Δ	Δ	Δ	Δ	Δ	Δ	
Bedtime Box Comparison Group			X	X	X	X	X	X	X	
Control Group			X	X	X	X	X	X	X	
Home Assessments (all groups)										
Questionnaires (e.g., BISQ, ECBQ)	X							X		
Sleep Actigraphy Watch	X	X						X	X	
Sleep Dairy	X	X						X	X	
Lab Assessments (all groups)										
Visual Search Task		X							X	
Antisaccade Task		X							X	
Gap-Overlap Task		X							X	
Mullen Scales of Early Learning		X							X	
Intervention Assessments										
Daily Screen Time Questionnaire (PASTI group)			Δ	Δ	Δ	Δ	Δ	Δ	Δ	
Bedtime Activity Diary (all groups)	X	X	X	X	X	X	X	X	X	
Parent Debrief Questionnaire (PASTI and Bedtime Box groups)										X

eFigure 1. Participant timeline

Notes. PASTI = Parent-Administered Screen Time Intervention. Dotted lines refer to the PASTI group only.

eAppendix 1. Eye-tracking experiment details

During the baseline and follow-up lab assessment, caregivers brought their toddler to the Birkbeck Babylab where they took part in three gaze-contingent eye-tracking experiments using an EyeLink 1000 Plus on a Windows PC. Stimuli were presented on a 24” widescreen monitor with stereo speakers via custom scripts using Experiment Builder. The toddlers were sat on their parent’s lap approximately 50-60cm from the screen. All sessions were video-recorded using a web camera, for data quality considerations. The eye-tracking experiments were administered in the following order.

1. ***Visual Search Task.*** The task was adapted[1] and has been used to measure visual attention and perception in toddlers. In this gaze-contingent version[2] the subject is presented with a search array until they fixate on the target (a red apple) or until 4 seconds has elapsed. The array can be a single feature array (mix of red and blue apples; set sizes 5 and 9), or a conjunction array (mix of red and blue apples and cropped apples; set sizes 5, 9 and 13). Some arrays also contained a salient distractor stimulus (e.g. bright blue apple; not analysed here). To grab participants’ attention and guarantee fixation at the centre of the screen, each trial starts with the target ‘flying in’ (800ms). When the infant attends to the central target it fades (750ms) and the trial array is presented. At the end of the trial the target spins and an audio reward is played (clapping; 1300ms). Trials were presented continuously, grouped into four blocks: (1) 3 single feature arrays, fixed order; (2) 1 single feature array, 9 conjunction arrays, randomised; (3) 4 single feature arrays, 9 conjunction arrays, randomised; and (4) 8 single feature distractor arrays, randomised. Saccadic reaction times to look at the target stimulus (red apple) for each correct trial were summed and divided by the total number of correct trials, for each set size (5, 9 and 13), array type (single – one feature search, exogeneous attentional control; conjunction – two feature search, endogenous attentional control) and distractor type (salient distractor vs. no salient distractor).

© 2024 Pickard H et al. *JAMA Pediatrics*.

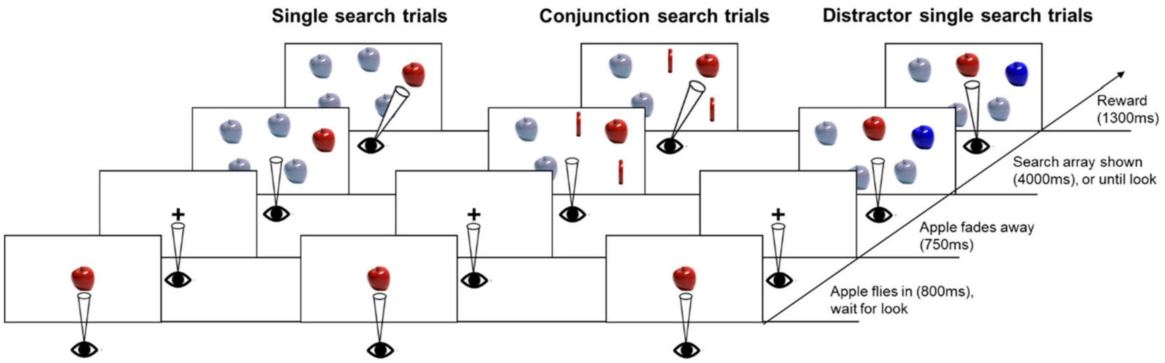

eFigure 2. Stimulus sequence for experimental trials in the Visual Search Task

2. ***Antisaccade Task.*** In this gaze-contingent anti-saccade paradigm[3] the infant is rewarded for looking in the opposite direction of the visual cue being displayed. Each trial starts with the presentation of a central animation (i.e. a star) to attract the infant's attention to the centre of the screen. Once the infant looks at the central stimulus (for 500ms), a distractor stimulus (black circle) appears on the left or right side of the screen for 200ms. 1000ms after the distractor disappears a target stimulus (red circle) appears on the opposite side of the screen. If the infant looks at the target stimulus, an attractive animal animation with sound replaces it (2500ms) and the trial ends. If the infant looks at the target-side before the target appears, then the animation automatically plays. If the infant doesn't look at the target/target-side no animation plays and the trial ends after 2000ms. The task is presented as a series of 2 consecutive blocks of 15 trials. Within participant, the distractor and target stimulus position (left/right) is fixed per block, and randomised across participants. In each trial, we determine (1) whether the infant looked at the distractor and (2) whether the infant looked at the target location before the target stimulus onset (anticipatory looking). The following metrics were calculated across all trials: (1) the proportion of looks towards the distractor not followed by an anticipatory look (i.e. pro-saccades); (2) the proportion of looks towards the distractor followed by an anticipatory look (i.e. corrective saccades); (3) the proportion of looks to the target in the absence of looks to the distractor (antisaccades, where inhibition of pro-saccades, as well as the production of contralateral saccades is required); and (4) average saccadic latency for prosaccades.

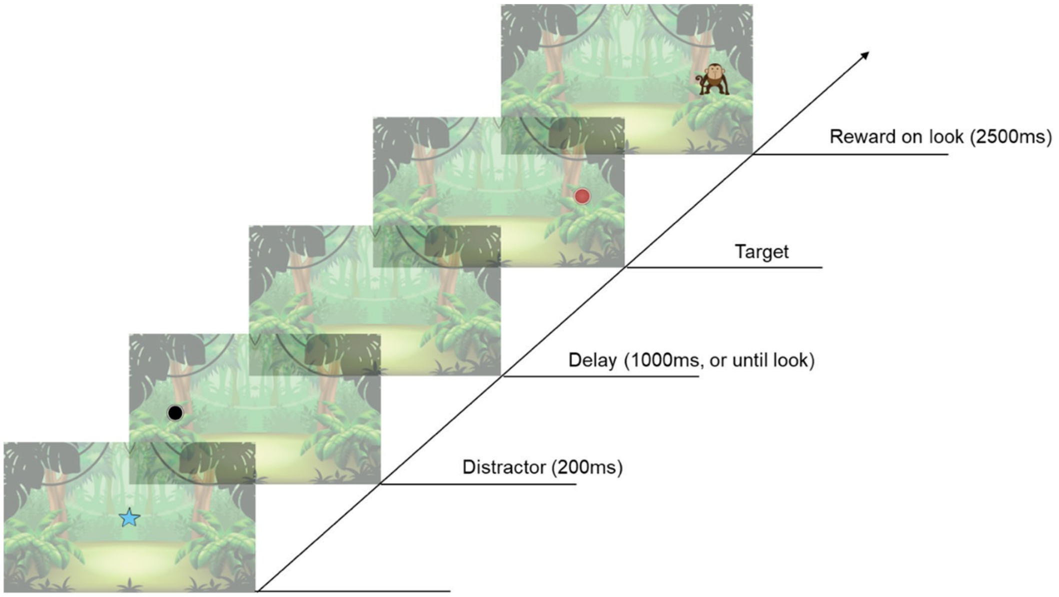

eFigure 3. Stimulus sequence for experimental trials in the Antisaccade Task.

3. ***Gap-overlap Task.*** The Gap-Overlap Task contains six blocks of 11 trials interleaved with free-viewing of static and dynamic scenes. In this task a central stimulus is presented

© 2024 Pickard H et al. *JAMA Pediatrics*.

followed by a peripheral target that appears either on the left or the right-hand side[3]. All trials begin with a central stimulus animation to attract the infant's attention to the centre of the screen. Once the infant has fixated on the central stimulus, a peripheral target is presented to the left- or right-hand side of the screen. There are three different trial conditions: (1) baseline trials, where the peripheral target appears immediately after the central stimulus disappears; (2) gap trials, where the central stimulus disappears and is followed by a gap (200ms) before the peripheral target appears; and (3) overlap trials, where the central stimulus remains on the screen when the peripheral target is also present. For 10% of trials the peripheral target is presented either on under or on top of the central stimulus to avoid anticipated looking (vertical trials), however, these are intended to break-up the sequence of lateral eye movements and are not used for analysis. When the infant looks at the peripheral target or after 4 seconds has elapsed, a novel animated stimulus plays (reward stimulus) and the trial ends. All trial conditions are presented pseudo-randomly within block: 30% of the trials are baseline trials, 30% of the trials are gap trials and 40% of the trials are overlap trials. A maximum of 60 trials (discounting the vertical trials) are presented. The central stimulus and background colour changed every block. Saccadic latencies are defined as the time from the presentation onset of the peripheral target to the first look to the peripheral target. The following outcome metrics are calculated: (1) Baseline RT, the average saccadic latency on baseline trials; (2) Overlap RT, the average saccadic latency on overlap trials; (3) Gap RT, the average saccadic latency on gap trials; and (4) Disengagement Effect, the subtraction of baseline RT from the overlap RT.

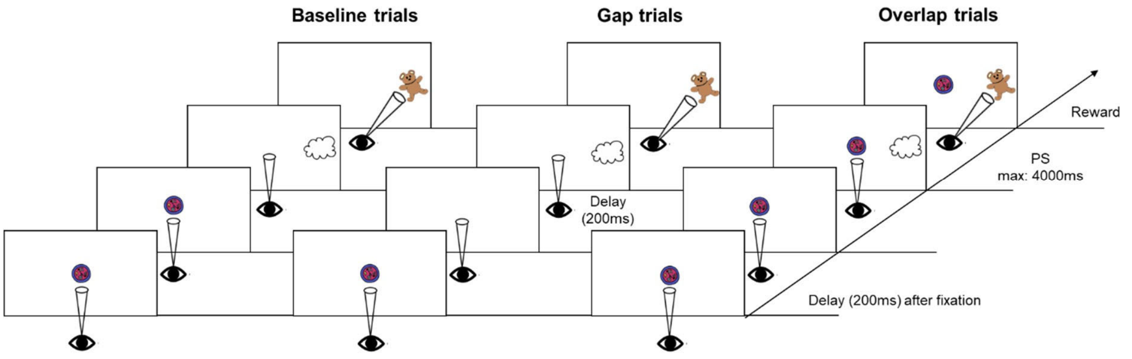

eFigure 4. Stimulus sequence for experimental trials in the Gap-Overlap Task.

eAppendix 2. Intervention delivery

Families were randomised into either PASTI, BB-only or No Intervention (NI) arm at the end of the baseline lab assessment. An unblinded researcher gave each family verbal instructions and a booklet outlining what they would be asked to do over the next seven weeks as part of the trial. The researcher script and the information in the booklet was largely matched across the PASTI, BB-only and NI arms, including details of the bi-weekly bedtime activity diary completed by all families. The PASTI and BB-only arms both received additional instructions about using the Family Bedtime Box in the hour before bed. The PASTI arm only received additional instructions to remove screen time in the hour before bed, along with details about completing the daily screen time questionnaire and the first week video/phone call. After families were given their verbal instructions and booklet, they all had the opportunity to ask the researcher questions. This process took approximately 10 to 15 minutes.

All families were asked to start the intervention on the following Monday to ensure a consistent start day across families. Throughout the trial, families were able to email the research team with questions (checked only by the unblinded researcher) or refer to an online Frequently Asked Questions (FAQs) resource provided in their booklets. Several FAQs were matched across all arms (e.g., visiting the Babylab, technical issues). The BB-only and PASTI arms had additional FAQs about the Family Bedtime Box and only the PASTI arm had FAQs relating to removing screen time.

© 2024 Pickard H et al. *JAMA Pediatrics*.

eAppendix 3. Family Bedtime Box

Families in the PASTI group received a Family Bedtime Box with tips on alternative pre-bed activities to help them displace screen time in the hour before bed (e.g., activity cards with activities centered around bath time, communication, crafts and gentle movement, as well as a selection of age-appropriate toys, such as crayons, a bath toy, puzzle; see e-Figure 5A). The activity cards for the PASTI arm contain a no screen time symbol, as well as suggested times for each activity card to aid with displacing screen time in the hour before bed (see e-Figure 5B for an example). Families in the BB-only group received identical materials (i.e. Family Bedtime Box) to the PASTI group, but ***without any screen time guidance.*** In addition, the selection of activity cards did not contain a screen time symbol or a time suggestion for each activity (see e-Figure 5C).

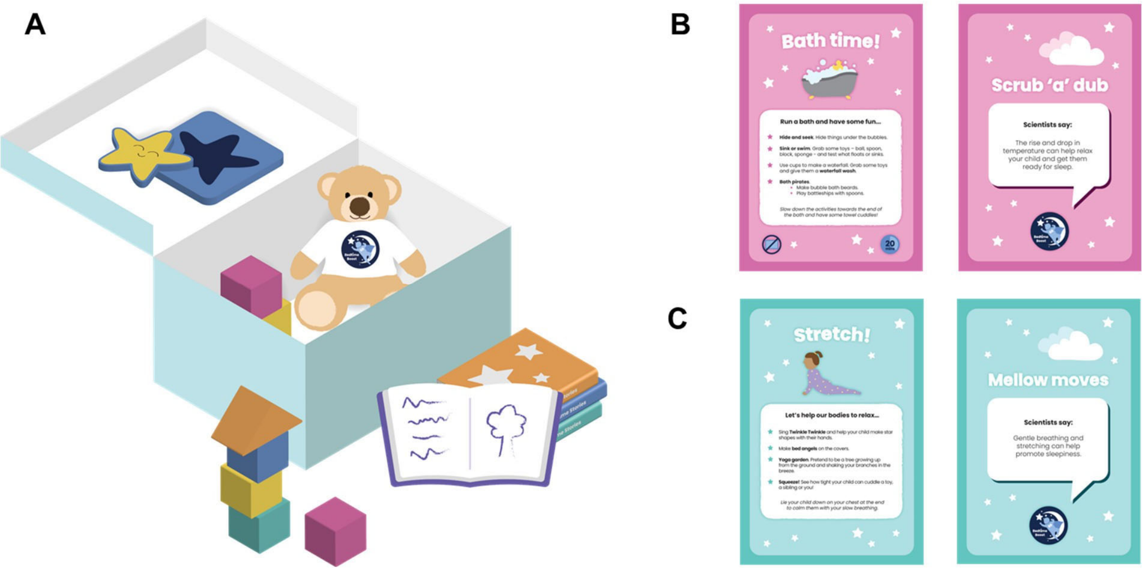

eFigure 5. Family Bedtime Box materials. A) An illustration of some toys/activities included in the Family Bedtime Box; B) An example of activity cards given to families in the PASTI group; and C) An example of activity cards given to families in the Bedtime Box group.

© 2024 Pickard H et al. *JAMA Pediatrics*.

eAppendix 4. Parent-Administered Screen Time Intervention (PASTI) development

The PASTI intervention, including intervention materials and support structures for families, was co-created with early years practitioners, a clinical psychologist, and parents through a series of iterative workshops and focus groups. Discussions with practitioners and parents included 1) how to help families remove screen time before bed, 2) how to deliver the intervention, 3) how to maximise parent engagement, and 4) how to support parents throughout the intervention. Together with practitioners, we developed the Family Bedtime Box, which included alternative pre-bed activities designed to help families in PASTI displace screen time in the hour before bed. This Family Bedtime Box was also delivered as an intervention to the BB-only group.

For the PASTI arm, early years practitioners suggested using a phone/video call during the first week of the intervention to support families with implementing PASTI, giving them the opportunity to reflect on their strategies for removing screen time and discuss any barriers/challenges they had experienced. Parents contributed ideas for the Family Bedtime Box and provided insights on how best to share and collect information throughout the trial (e.g. via text message). The insights gained through the co-creation process shaped the development and implementation of PASTI and were important for ensuring that the trial was feasible for families.

Feedback provided by families in the PASTI debrief questionnaire showed that 84% agreed (strongly-to-somewhat agreed) that the phone/video call was helpful and 97% agreed that they felt supported during the trial. Quotes from families supported this: “The phone call at the start was very informative and I knew I could always reach out to someone” and “I felt well supported and knew that I could easily contact the team if necessary but it was quite straight forward so I didn’t really need anything”. Out of the PASTI families, 100% agreed that it was easy to complete the daily screen time questionnaire, and 91% agreed that they were happy to receive questionnaire/support via text message. For example: “I found it very easy to complete the questionnaires as they were sent via text so it was really quick and simple” and “Everything was easy, I appreciated the contact being via text as I didn’t have to remember anything!”.

© 2024 Pickard H et al. *JAMA Pediatrics*.

eAppendix 5. Screen use questions used in the Screen Use Questionnaire and Bedtime Activity Diary.

How long did your child have any screen time for in the hour before bed today (e.g., watching, playing-with, or looking-at a screen including TV, tablets, phones, laptops, etc.)? The screen could be on for them or someone else in the room.	0-60 minutes
Which screen time device(s) did your child view in the hour before bed today?<br><br>Please tick all that apply.	□ TV – played for them<br>□ Background TV – played for somebody else<br>□ Tablet/iPad<br>□ Smartphone<br>□ Computer/laptop<br>□ Other
What did your child have screen time for in the hour before bed today?	□ Passively watch tv/videos<br>□ Scroll or touch the screen<br>□ Call someone (e.g. phone call)<br>□ Video call someone (e.g. Zoom, FaceTime)<br>□ Play games<br>□ Use educational apps<br>□ Use art/creative apps such as drawing<br>□ Listen to music<br>□ Look at photos<br>□ Take photos<br>□ Web search (e.g. Google)<br>□ Text message (e.g. WhatsApp)<br>□ Social media (e.g. Instagram)<br>□ Other:

© 2024 Pickard H et al. *JAMA Pediatrics*.

eAppendix 6. Parent-reported screen time measure

To reduce reporter-bias we constructed questions according to the principles laid out by Morsbach & Prinz (2006)[4]. For example, questions in PASTI were asked via text messaging, and caregivers were asked to report on immediately preceding activities (i.e. from that evening), to reduce recall bias. We emphasised to families in the intervention arm that one of our aims was to assess whether the intervention was feasible. Caregivers were asked "Please be as honest as possible and let us know if your child did or didn't have screen time in the hour before bed." We made it clear in the initial discussion of the intervention that if screen time was used it was important to let us know so that we could make changes to our design. In line with this, the average proportion of nights without screen time during the intervention period in the PASTI arm was 89% [95% CI 84% - 94%], suggesting that parents felt able to report when their child had screen time before bed (on average 11% of nights during the intervention period).

To put the parent-reported screen time (median of 13 minutes at baseline; see e-Table 1; Supplement 10) in context, the other pre-bed activities reported by parents are presented in e-Table 2 (Supplement 11).

© 2024 Pickard H et al. *JAMA Pediatrics*.

eTable 1. Descriptives for baseline and follow up secondary outcomes.

Secondary outcome	Summary [N avail]	Baseline				Follow-up			
		PASTI (N=35)	BB-only (N=36)	NI (N=34)	Overall (N=105)	PASTI (N=35)	BB-only (N=36)	NI (N=34)	Overall (N=105)
Screen time									
Average screentime in hour before bed	median (IQR) [N]	9 (4-24) [29]	18 (9-23) [28]	11 (1-20) [30]	13 (4-23) [87]	0 (0-0) [29]	7 (0-16) [27]	12 (1-21) [31]	4 (0-16) [87]
Sleep									
Average total night-time sleep duration (mins)	mean (sd) [N]	606 (49) [31]	606 (54) [32]	601 (39) [32]	604 (48) [95]	596 (55) [27]	595 (51) [27]	590 (37) [27]	594 (48) [81]
Average total day-time sleep duration (mins)	mean (sd) [N]	86 (49) [29]	74 (50) [24]	75 (34) [21]	79 (45) [74]	82 (54) [23]	75 (50) [20]	84 (33) [22]	80 (46) [65]
Average night awakenings	median (IQR) [N]	1 (0-2) [31]	0 (0-1) [32]	0 (0-1) [32]	0 (0-1) [95]	1 (0-1) [27]	1 (0-1) [27]	1 (0-1) [27]	1 (0-1) [81]
Average sleep efficiency	median (IQR) [N]	88 (84-90) [31]	88 (87-90) [32]	89 (87-90) [32]	89 (87-90) [95]	88 (86-89) [27]	87 (86-88) [27]	88 (86-89) [27]	87 (86-89) [81]
BISQ-R Sleep onset latency (minutes)	median (IQR) [N]	30 (15-60) [35]	30 (20-60) [36]	30 (15-40) [34]	30 (15-45) [105]	20 (10-45) [34]	25 (15-52) [36]	20 (15-32) [32]	20 (15-45) [102]
Attention									
VST single search reaction time (ms)	median (IQR) [N]	1027 (801-1221)	936 (847-1264)	998 (782-1194)	998 (801-1212)	998 (764-1165)	1043 (776-1179)	996 (696-1268)	1009 (746-1177)

© 2024 American Medical Association. All rights reserved.

		[34]	[36]	[34]	[104]	[33]	[35]	[32]	[100]
AT prosaccade saccadic reaction time (pre-switch)	mean (sd) [N]	317 (32) [31]	310 (35) [34]	318 (29) [33]	315 (32) [98]	304 (33) [31]	303 (44) [31]	305 (38) [26]	304 (38) [88]
AT proportion antisaccades (pre-switch)	median (IQR) [N]	20 (0-40) [33]	0 (0-27) [35]	17 (7-38) [33]	12 (0-33) [101]	23 (0-44) [31]	10 (0-40) [31]	20 (0-43) [28]	15 (0-40) [90]
GT baseline reaction time (ms)	median (IQR) [N]	340 (286-384) [30]	326 (296-376) [31]	338 (312-369) [32]	335 (299-374) [93]	336 (295-375) [31]	321 (289-353) [27]	332 (308-364) [25]	326 (295-364) [83]
GT disengagement reaction time (ms)	mean (sd) [N]	132 (96) [30]	142 (98) [31]	153 (87) [32]	143 (93) [93]	116 (93) [31]	100 (118) [27]	107 (83) [25]	108 (98) [83]
ECBQ SF effortful control	mean (sd) [N]	4.7 (0.6) [35]	4.6 (0.5) [36]	4.6 (0.5) [34]	4.6 (0.5) [105]	4.8 (0.7) [34]	4.9 (0.6) [36]	4.6 (0.4) [32]	4.8 (0.6) [102]
ECBQ SF inhibitory control	mean (sd) [N]	3.8 (1.2) [35]	3.7 (1.2) [36]	3.8 (1.1) [34]	3.8 (1.1) [105]	3.7 (1.2) [34]	4.2 (1.1) [36]	3.9 (0.7) [32]	3.9 (1.0) [102]

Notes. PASTI = Parent-Administered Screen Time Intervention. BB = Bedtime Box. NI = No Intervention. BISQ-R = Brief Infant Sleep Questionnaire – Revised. VST = Visual Search Task. AT = Antisaccade Task. GT = Gap-Overlap Task. ECBQ = Early Childhood Behaviour Questionnaire.

© 2024 American Medical Association. All rights reserved.

eTable 2. Descriptives for alternative activities in the hour before bed from the Bedtime Activity Diary

Outcome	Summary [N avail]	Baseline				Follow-up			
		PASTI (N=35)	BB-only (N=36)	NI (N=34)	Overall (N=105)	PASTI (N=35)	BB-only (N=36)	NI (N=34)	Overall (N=105)
Average playtime (e.g. crafts, puzzle) in hour before bed	median (IQR) [N]	16 (11-27) [29]	11 (5-33) [28]	13 (5-24) [30]	14 (6-26) [87]	20 (7-33) [29]	19 (11-31) [27]	10 (5-16) [31]	16 (6-30) [87]
Average eat-time in hour before bed	median (IQR) [N]	7 (0-14) [29]	6 (0-12) [28]	4 (0-13) [30]	5 (0-13) [87]	1 (0-10) [29]	4 (0-9) [27]	5 (1-10) [31]	4 (0-10) [87]
Average read time in hour before bed	median (IQR) [N]	10 (3-11) [29]	6 (2-10) [28]	10 (5-14) [30]	7 (4-11) [87]	10 (6-13) [29]	5 (3-11) [27]	7 (5-13) [31]	7 (4-13) [87]
Average wind-down (e.g. listening to music, massage) in hour before bed	median (IQR) [N]	7 (0-10) [29]	5 (1-13) [28]	6 (0-9) [30]	5 (0-10) [87]	6 (2-11) [29]	5 (1-10) [27]	6 (0-10) [31]	6 (1-10) [87]
Average bed-readying (e.g. bath, pyjamas) time in hour before bed	median (IQR) [N]	15 (9-22) [29]	10 (8-20) [28]	14 (10-19) [30]	13 (10-20) [87]	15 (9-20) [29]	13 (9-17) [27]	13 (6-28) [31]	13 (9-20) [87]

Notes. PASTI = Parent-Administered Screen Time Intervention. BB = Bedtime Box. NI = No Intervention.

© 2024 American Medical Association. All rights reserved.

eFigure 6. Forest plot of effect sizes for Bedtime Box vs No Intervention comparison.

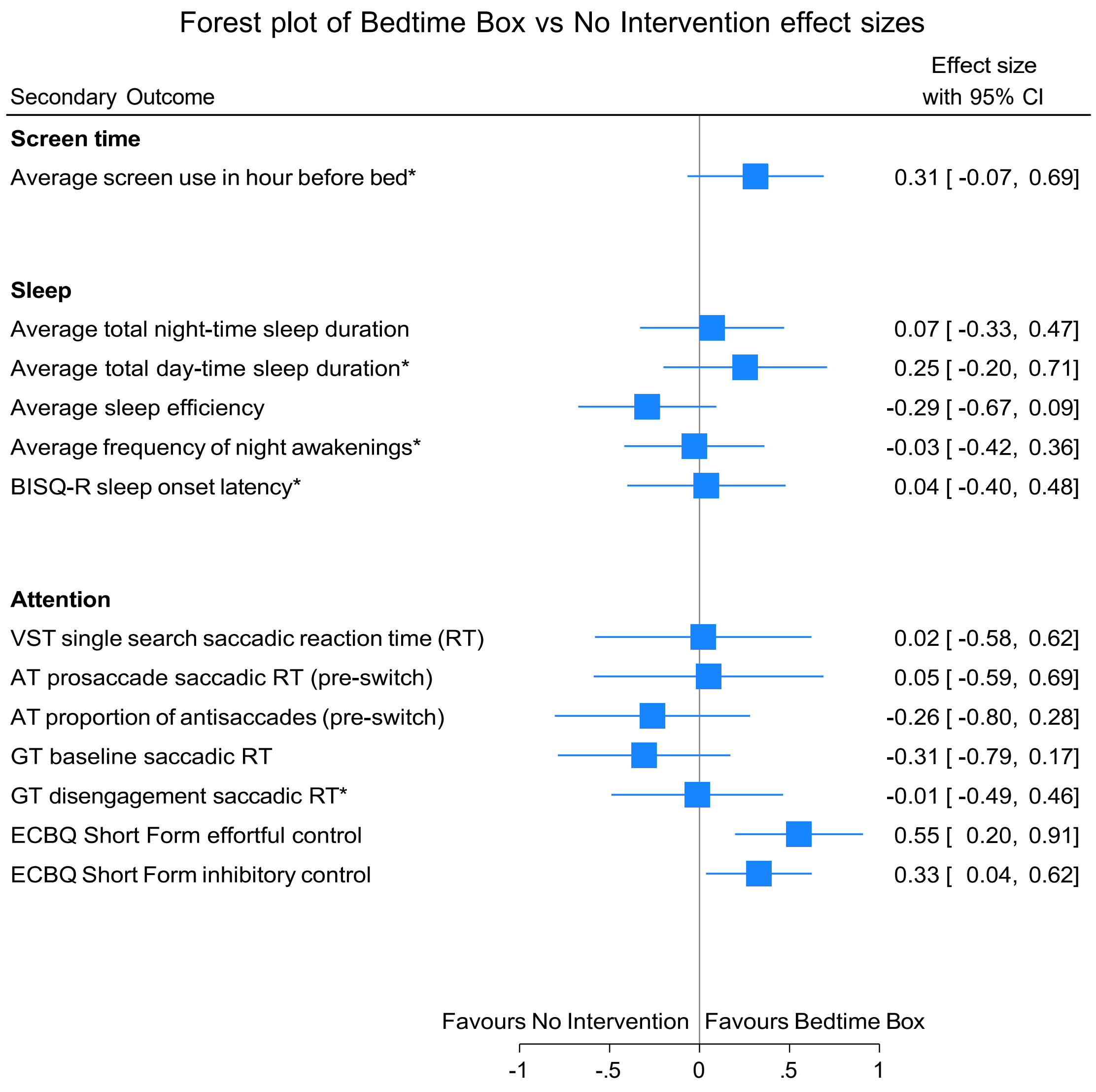

*Reversed direction

© 2024 American Medical Association. All rights reserved.

eReferences

1. Kaldy Z, Kraper C, Carter AS, Blaser E. Toddlers with autism spectrum disorder are more successful at visual search than typically developing toddlers. *Developmental science*. 2011(5):980-8. 10.1111/j.1467-7687.2011.01053.x
2. Portugal AM, Bedford R, Cheung CH, Gliga T, Smith TJ. Saliency-driven visual search performance in toddlers with low–vs high–touch screen use. *JAMA pediatrics.* 2021;175(1):96-7. 10.1001/jamapediatrics.2020.2344
3. Portugal AM, Bedford R, Cheung CH, Mason L, Smith TJ. Longitudinal touchscreen use across early development is associated with faster exogenous and reduced endogenousattention control. *Scientific Reports.* 2021;11(1):2205. 10.1038/s41598-021-81775-7
4. Morsbach, S. K., & Prinz, R. J. (2006). Understanding and improving the validity of self-report of parenting. *Clinical Child and Family Psychology Review*, *9*, 1-21.

© 2024 American Medical Association. All rights reserved.
