# Supplementary material for: Toddler Screen Use Before Bed and Its Effect on Sleep and Attention: A Randomized Clinical Trial
Source: JAMA Pediatr. 2024 Oct 21;178(12):1270–9. doi: 10.1001/jamapediatrics.2024.3997 (PMC11581737; doi:10.1001/jamapediatrics.2024.3997)
Supplement: Supplement 4. — Data Sharing Statement. [file jamapediatr-e243997-s004.pdf]

Data Sharing Statement

Pickard. Toddler Screen Use Before Bed and Its Effect on Sleep and Attention. *JAMA Pediatr*. Published October 21, 2024. doi:10.1001/jamapediatrics.2024.3997

Data
Data available: Yes
Data types: Participant data with identifiers, Data dictionary
How to access data: https://researchdata.bbk.ac.uk/
When available: With publication

Supporting Documents
Document types: Informed consent form
How to access documents: https://osf.io/vnt6k/?view_only=144a15ce466a4836a81983476b8d9fa0
When available: With publication

Additional Information
Who can access the data: Anyone requesting the data
Types of analyses: For any purpose
Mechanisms of data availability: Without investigator support
